# Identified risk factors for dry eye syndrome: A systematic review and meta-analysis

**DOI:** 10.1371/journal.pone.0271267

**Published:** 2022-08-19

**Authors:** Lijun Qian, Wei Wei

**Affiliations:** 1 Department of Ophthalmology, Jinhua Hospital of Traditional Chinese Medicine, Jinhua, China; 2 Nanjing University of Chinese Medicine, Nanjing, China; 3 Department of Ophthalmology, Hospital Affiliated to Nanjing University of Chinese Medicine, Nanjing, China; University of Toronto, CANADA

## Abstract

A meta-analytic approach was used to identify potential risk factors for dry eye syndrome. PubMed, Embase, and the Cochrane library were systematically searched for studies investigated the risk factors for dry eye syndrome from their inception until September 2021. The odds ratio (OR) with 95% confidence interval (CI) was calculated using the random-effects model. Forty-eight studies comprising 493,630 individuals were included. Older age (OR: 1.82; *P*<0.001), female sex (OR: 1.56; *P*<0.001), other race (OR: 1.27; *P*<0.001), visual display terminal use (OR: 1.32; *P*<0.001), cataract surgery (OR: 1.80; *P*<0.001), contact lens wear (OR: 1.74; *P*<0.001), pterygium (OR: 1.85; *P* = 0.014), glaucoma (OR: 1.77; *P* = 0.007), eye surgery (OR: 1.65; *P*<0.001), depression (OR: 1.83; *P*<0.001), post-traumatic stress disorder (OR: 1.65; *P*<0.001), sleep apnea (OR: 1.57; *P* = 0.003), asthma (OR: 1.43; *P*<0.001), allergy (OR: 1.38; *P*<0.001), hypertension (OR: 1.12; *P* = 0.004), diabetes mellitus (OR: 1.15; *P* = 0.019), cardiovascular disease (OR: 1.20; *P*<0.001), stroke (OR: 1.32; *P*<0.001), rosacea (OR: 1.99; *P* = 0.001), thyroid disease (OR: 1.60; *P*<0.001), gout (OR: 1.40; *P*<0.001), migraines (OR: 1.53; *P*<0.001), arthritis (OR: 1.76; *P*<0.001), osteoporosis (OR: 1.36; *P* = 0.030), tumor (OR: 1.46; *P*<0.001), eczema (OR: 1.30; *P*<0.001), and systemic disease (OR: 1.45; *P* = 0.007) were associated with an increased risk of dry eye syndrome. This study reported risk factors for dry eye syndrome, and identified patients at high risk for dry eye syndrome.

## Introduction

Dry eye syndrome (DES) is defined as a multifactorial disease of the tears and ocular surface that could cause discomfort and visual disturbance, with potential damage to the ocular surface. These symptoms could affect quality of life and activities of daily living [1, 2]. The prevalence of DES is increasing and is seen in nearly one in five adults. Thus, this needs more attention from ophthalmologists [3, 4]. The role of the tear film has already been demonstrated. It has been shown to provide lubrication to the eyes, as well as nutrition and oxygen, and eliminate debris from the ocular surface [5]. Moreover, individuals with dry eyes also suffer from systemic diseases [4]. However, the prevalence of dry eyes is often underestimated because of varying presentation and symptoms [6]. Studies have demonstrated that age and sex are significantly associated with increased risk of DES; however, the pathogenesis of DES is not fully understood [7, 8].

Several studies have already identified risk factors for DES. Major risk factors include older age, female sex, having undergone postmenopausal estrogen therapy or ocular surface surgery, and using antihistamine medications [9]. Moreover, the occupational risk factor of visual display terminal (VDT) use was related to the progression of DES, which could be explained by a decreased blink rate and increased proportion of incomplete blinks that could be caused by the increased exposure of the ocular surface to the environment. Outdoor environments, sunlight, and air pollution in tropical countries are also associated with an elevated risk of DES [10, 11]. Furthermore, other risk factors for DES include vitamin D deficiency and diabetes mellitus (DM) [12, 13]. However, whether the comorbidities of individuals could affect the risk of DES remained controversial. We, therefore, performed a systematic review and meta-analysis to independently identify risk factors for DES.

## Methods

### Data sources, search strategy, and selection criteria

The current study was performed and reported following the Preferred Reporting Items for Systematic Reviews and Meta-Analysis Statement [14]. Studies reporting the risk factors of DES were eligible in our study, and publication language was restricted to English. PubMed, Embase, and the Cochrane library were systematically searched for eligible studies from their inception until September 2021, and using the following text word or Medical Subject Heading terms: "dry eye syndrome", "dry eye disease", "Keratoconjunctivitis Sicca", "Xerophthalmia", and "Risk Factors". The details of search strategy in PubMed are listed in S1 File. The reference lists of relevant original and review articles were manually screened to identify further eligible studies.

Two reviewers (QL and WW) independently performed study assessment following a standardized approach. Any disagreement between reviewers was settled by discussion until a consensus was reached. A study was included if the following criteria were met: (1) it was a cross-sectional, retrospective, or prospective observational study; (2) risk factors were reported for ≥ 3 studies [15] and included such factors as age, sex, race, residence, education level, obesity, dyslipidemia, alcohol, smoking, VDT use, cataract surgery, contact lens wear, pterygium, glaucoma, age-related maculopathy, eye surgery, depression, post-traumatic stress disorder (PTSD), sleep apnea, asthma, allergy, hypertension, DM, cardiovascular disease (CVD), stroke, rosacea, thyroid disease, chronic obstructive pulmonary disease (COPD), gout, migraines, arthritis, osteoporosis, tumor, meibomian gland dysfunction (MGD), eczema, and systemic disease; and (3) it reported effect estimates (relative risk [RR], hazard ratio [HR], or odds ratio [OR]) and 95% confidence interval (CI) for risk factors of DES. Interventional study, animal study, review, and letter to editor was excluded.

### Data collection and quality assessment

Two reviewers (QL and WW) independently abstracted the following items, including study group or first author’s name, publication year, country, study design, sample size, age, % of males, population status, % of DES cases, definition of DES, risk factors, adjusted factors, and reported effect estimates. The effect estimate with maximal adjustment for potential confounders was selected if a study reported several multivariable-adjusted effect estimates. Study quality was assessed using the Newcastle-Ottawa Scale (NOS), which has already been validated for assessing the quality of observational studies in meta-analysis [16]. A total of 8 items in 3 subscales were included in NOS. The star system in each study ranged from 0–9. Inconsistent results for the data abstracted and quality assessment between the two reviewers were settled following mutually discussion referred to the original article.

### Statistical analysis

Identified risk factors for DES were analyzed based on the OR, RR, or HR, with its 95% CI, in individual studies. Then the pooled ORs with 95%CI were calculated using the random-effects model [17, 18]. *I*^*2*^ and Q statistic were applied to assess heterogeneity across included studies. Significant heterogeneity was defined as *I*^*2*^> 50.0% or *P* < 0.10 [19, 20]. Sensitivity analysis was performed for factors reported in ≥ 4 studies to assess the robustness of pooled conclusion through sequentially removing individual studies [21]. Subgroup analyses were performed for factors reported in ≥ 4 studies on the basis of the country. The difference between subgroups was assessed using the interaction *P* test [22]. Visual inspections of funnel plots for factors reported in ≥ 4 studies were performed to qualitatively assess publication bias. The Egger or Begg tests were used to quantitatively assess publication bias [23, 24]. The *P-*value for all pooled results was 2-sided, and the inspection level was 0.05. All of the statistical analysis in our study was performed using software STATA (version 12.0; Stata Corporation, College Station, TX, USA).

## Results

### Literature search

**Fig 1:**
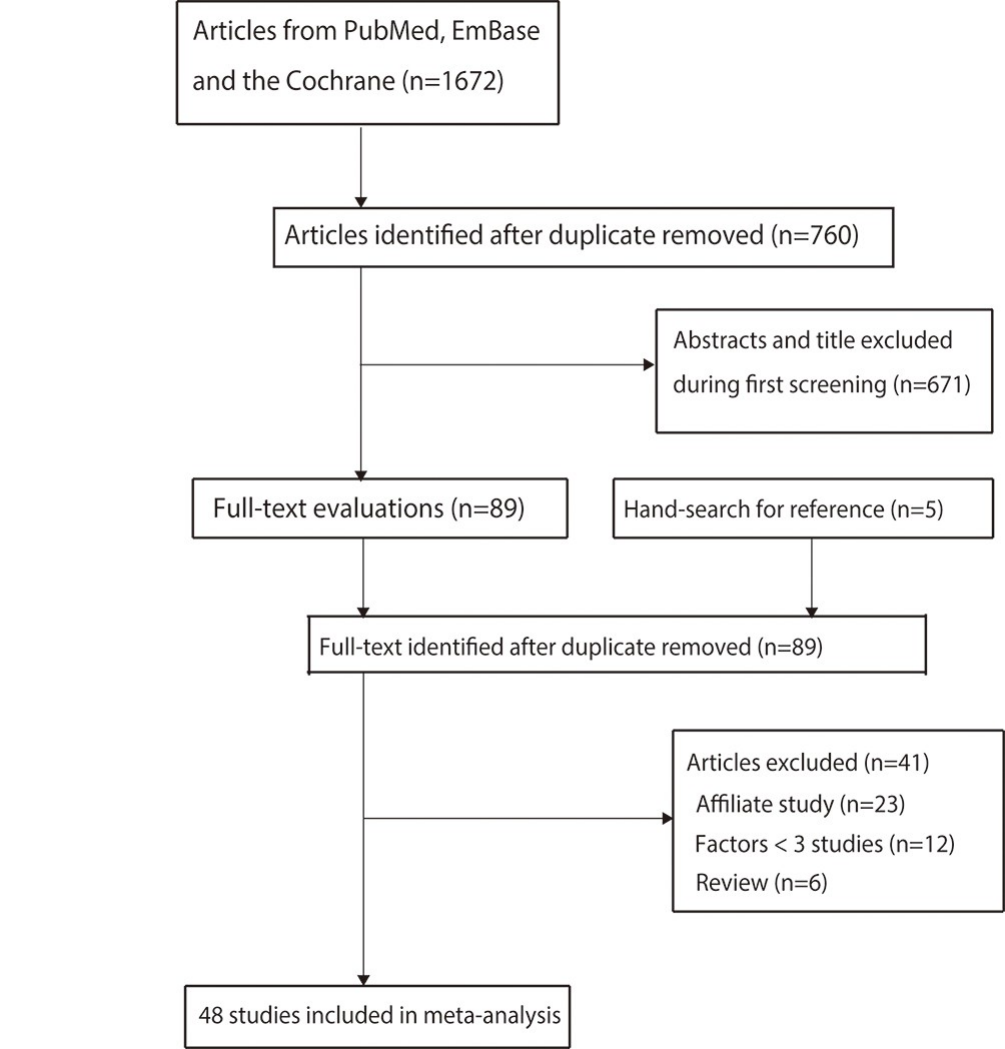
Details of the literature search and study selection processes.

A total of 1,672 studies were identified from initial electronic searches. Details of the study selection process are presented in Fig 1. Of these, 912 articles were removed because they were duplicates. A further 671 articles were excluded owing to irrelevant titles or abstracts. The remaining 89 studies were retrieved for full-text evaluations, with 41 studies removed because of: affiliate study (n = 23), evaluated factors < 3 studies (n = 12), and review-type articles (n = 6). A manual search of the reference lists of relevant articles did not yield any additional studies. Finally, 48 studies were selected for the final meta-analysis [25–71]. Characteristics of the included studies and involved individuals are summarized in Table 1.

**Table 1:** The baseline characteristics of included studies.

Study	Country	Study design	Sample size	Age (years)	Male (%)	Population	DES (%)	Definition of DES	Reported factors	Adjusted factors
BDES 2000 [25]	USA	C	3,722	65.0	43.0	PB	14.4	Questionnaire	DM, arthritis, TD, osteoporosis, gout, ES, CLW, alcohol, smoking	Age and sex
Lee 2002 [26]	Indonesia	C	1,058	37.0	47.7	PB	27.5	Questionnaire	Sex, smoking, pterygium	Sex, age, occupation, smoking, and pterygium
BMES 2003 [27]	Australia	C	1,174	60.8	44.2	PB	57.5	Questionnaire	Arthritis, asthma, DM, gout, smoking, alcohol	Age and sex
Sahai 2005 [28]	India	C	500	> 20.0	55.2	HB	18.4	Questionnaire	Smoking	Age and sex
Nichols 2006 [29]	USA	C	360	31.1	32.0	HB	55.3	Questionnaire	Sex	Nominal water content, PLTF
Uchino 2008 [30]	Japan	C	3,549	22.0–60.0	74.4	PB	10.1	Questionnaire	Age, sex, VDT, systemic disease, smoking, contact lens	Age, gender, VDT use, systemic disease systemic medication, smoking, contact lens use
Lu 2008 [31]	China	C	1,840	56.3	56.0	PB	52.4	TFBT, ST, FSS	Age, education level, smoking alcohol	Crude
PHS 2009 [32]	USA	C	25,444	64.4	100.0	PB	23.0	Questionnaire	Age, race, hypertension, tumor, DM	Crude
TSES 2009 [33]	Spain	C	654	63.6	37.2	PB	11.0	Questionnaire	Sex, VDT use, CLW, rosacea, allergy, DM, hypertension, COPD, education level, alcohol, smoking	Age and sex
BES 2009 [34]	China	C	1,957	56.5	43.1	PB	21.0	Questionnaire	Sex, residence, glaucoma, MD, DM, hypertension, smoking, alcohol	Age, sex, region, undercorrection of refractive error, and nuclear cataract
THES 2010 [35]	China	C	1,816	54.9	53.9	PB	50.1	TBUT, ST, FSS	Pterygium, age, sex, education level, smoking, alcohol	Crude
Kim 2011 [36]	Korea	C	650	71.9	48.3	PB	30.5	Questionnaire	Sex, residence, depression, MGD	Crude
Koumi Study 2011 [37]	Japan	C	2,791	> 40.0	43.7	PB	16.5	Questionnaire	Age, smoking, alcohol, BMI, education level, VDT use, CLW, stroke, CVD, hypertension, DM	Age, smoking, alcohol, BMI, education level, VDT use, CLW, stroke, CVD, hypertension, DM
USVAP 2011 [38]	USA	R	16,862	NA	NA	PB	12.2	ICD9 code	Sex, race, DM, hypertension, dyslipidemia, CVD, stroke, PTSD, depression, alcohol, arthritis, gout, TD, tumor, sleep apnea, rosacea, glaucoma	Age and sex
Zhang 2012 [39]	China	C	1,885	< 18.0	50.8	PB	23.7	Questionnaire	CLW, sleep apnea	CLW, sleep apnea, myopia, inadequate refractive correction, topical ophthalmic medication
TNHRI 2012 [40]	China	R	48,028	52.4	26.6	PB	25.0	ICD9 code	Hypertension,CVD, dyslipidemia, stroke, migraines, arthritis, COPD, asthma, DM, TD, depression, and tumor	Age, sex, region, and incomes
TOS 2013 [41]	Japan	C	561	43.3	66.7	PB	11.6	Questionnaire	Sex, age, smoking, VDT use, CLW, systemic disease, hypertension	Sex, age, smoking, VDT use, CLW, systemic disease, hypertension
TwinUK 2014 [42]	UK	C	3,824	57.1	0.0	PB	9.6	Questionnaire	CLW, CS, glaucoma, MD, osteoporosis, asthma, allergy, TD, arthritis, dyslipidemia, hypertension, DM, cancer, stroke, migraine, depression	Age
KNHNES 2014 [43]	Korea	C	11,666	49.9	42.8	PB	8.0	Questionnaire	Age, sex, education level, residence, hypertension, obesity, dyslipidemia, arthritis, TD, smoking, alcohol, sleep apnea, ES	Age, sex, education level, residence, hypertension, obesity, dyslipidemia, arthritis, TD, smoking, alcohol, sleep apnea, ES
Moon 2014 [44]	Korea	C	288	10.9	49.3	PB	9.7	Questionnaire	VDT use	Age, and sex
BDOS 2014 [45]	USA	C	3,275	49.0	45.4	PB	14.5	Questionnaire	Age, sex, CLW, arthritis, allergies, TD, migraine	Age, and sex
TNHI 2015 [46]	China	R	10,325	61.9	36.7	PB	20.0	ICD9 code	DM, hypertension, dyslipidemia, CVD	DM, hypertension, dyslipidemia, CVD
Yang 2015 [47]	China	R	1,908	56.2	41.4	HB	41.4	TFBT, ST, and FSS	DM, arthritis, tumor, acne rosacea, PTSD, VDT use	DM, arthritis, tumor, acne rosacea, PTSD, VDT use
Tan 2015 [48]	Singapore	C	1,004	38.2	44.1	PB	12.3	Questionnaire	Sex, age, CLW, alcohol	Crude
Shah 2015 [49]	India	C	400	58.6	48.0	HB	54.3	TBUT	DM, ES, MGD	Occupation, indoor table work, DM previous ocular surgery, MGD
Olaniyan 2016 [50]	Nigeria	C	363	59.1	48.2	PB	32.5	Questionnaire	Age, ES	Age, work place, medication use, ocular surgery, postmenopausal state
Alshamrani 2017 [51]	Saudi Arabia	C	1,858	39.3	48.0	PB	32.1	Questionnaire	Sex, age, residence, smoking, CLW, DM, hypertension, asthma, CVD, TD, arthritis, gout, osteoporosis	Sex, age, residence, work status, smoking, currently wearing, and history of trachoma
NHWS 2017 [52]	USA	C	73,211	> 18.0	48.4	PB	6.9	Questionnaire	Age, sex, race, education level	Age and sex
SMES 2017 [53]	Singapore	P	1,682	56.9	44.6	PB	5.1	Questionnaire	DM, hypertension, smoking, CLW, stroke, CVD, TD, glaucoma, MGD, pterygium	Sex, age, income, smoking, CLW, cataract surgery, thyroid disease
Gong 2017 [54]	China	C	1,015	54.6	29.7	PB	27.8	Questionnaire	VDT use, DM, hypertension, arthritis, smoking, alcohol	Sex, age, VDT use, DM, hypertension, arthritis, dry mouth, smoking, alcohol, and spicy diets
Asiedu 2017 [54]	Ghana	C	650	22.0	66.6	PB	44.3	Questionnaire	Age, sex, allergies, alcohol, VDT use	Age, sex, allergies, alcohol, VDT use
Graue-Hernandez 2018 [55]	Mexico	C	1,508	64.7	40.3	PB	41.1	Questionnaire	Sex, smoking, DM, alcohol, hypertension	Sex, smoking, DM, alcohol, hypertension
SES 2018 [56]	Spain	C	264	56.8	32.7	PB	25.4	TBUT, ST, FSS	Sex, education level, VDT use, alcohol, smoking, hypertension, DM, COPD, CVD, TD, rosacea	Age
Iglesias 2018 [57]	USA	R	86	71.0	95.0	HB	32.1	Questionnaire	Race, DM, depression, PTSD, sleep apnea, glaucoma	Crude
TMS 2018 [58]	France	C	1,045	82.2	71.8	PB	34.4	Questionnaire	Obesity, smoking, alcohol, education level, hypertension, DM, depression, CS, MD, glaucoma	Age, and sex
Shehadeh-Mashor 2019 [59]	Israel	R	25,317	27.0	55.0	PB	6.0	TBUT, and ST	Sex, CLW	Age and sex
Zhang 2019 [60]	China	C	31,124	NA	49.1	HB	57.6	ST, and FSS	Sex, age, DM, arthritis, TD, ES	Sex, age, refractive surgery
Yasir 2019 [61]	Saudi Arabia	C	890	> 40.0	55.5	PB	35.9	Questionnaire	Glaucoma, DM, and hypertension	Crude
HTS 2019 [62]	Japan	C	356	55.5	37.4	PB	33.4	Questionnaire	Sex, smoking, CLW, hypertension	Sex, eye makeup use, smoking CLW, hypertension, sleeping pills
Hyon 2019 [63]	Korea	C	232	> 20.0	15.1	PB	42.7	Questionnaire	Sex, VDT use	Sex, and VDT use
Ben-Eli 2019 [64]	Israel	R	331	53.6	24.8	HB	36.3	Clinician-diagnosed	Smoking, alcohol	Ethnicity, smoking, alcohol, hospitalization for infection
Yu 2019 [65]	China	C	23,922	NA	48.8	HB	61.6	TBUT, and FSS	Sex, age, ES, arthritis, TD	Humidity, air pressure, and air temperature
Rossi 2019 [66]	Italy	C	194	41.8	34.5	HB	16.5	TBUT, and FSS	Sex, VDT use	Age, sex, VDT use, visual acuity, and presbyopia
Wang 2020 [67]	New Zealand	C	372	39.0	40.3	PB	29.0	Clinician-diagnosed	Sex, CLW, anxiety, asthma, DM, depression, dyslipidemia, hypertension, cancer, migraine, TD, CS, ES	Age, CLW, ethnicity, migraine, menopause, systemic disease, thyroid disease, antidepressant medication, and oral contraceptive therapy
Shanti 2020 [68]	Palestine	C	769	43.6	47.3	PB	64.0	TBUT, ST, FSS	Sex, VDT use, smoking, DM, hypertension	Age, sex, VDT use, smoking, systemic disease
JPHC 2020 [69]	Japan	P	102,582	58.3	46.2	PB	24.6	Questionnaire	VDT use	Age, smoking, education status, income, and public health area
Alkabbani 2021 [70]	United Arab Emirates	C	452	> 17.0	36.3	PB	62.6	Questionnaire	Age, sex, CLW, ES, VDT use, smoking	Age, sex, CLW, ES, VDT use, smoking
LCS 2021 [71]	Netherlands	C	79,866	50.4	40.8	PB	9.1	Questionnaire	Sex, CLW, MD, glaucoma, ES, CS, arthritis, gout, CVD, stroke, migraine, depression, PTSD, COPD, asthma, sleep apnea, rosacea, allergy, DM, osteoporosis, TD, anemia	Age, and sex

*BMI: body mass index; C: cross-sectional; CLW: contact lens wear; COPD: chronic obstructive pulmonary disease; CS: cataract surgery; CVD: cardiovascular disease; DM: diabetes mellitus; ES: eye surgery; FSS: fluorescein staining score; HB: hospital-based; MD: macular degeneration; MDG: meibomian gland dysfunction; MI: myocardial infarction; NA: not available; P: prospective; PB: population-based; PLTF: prelens tear film; PTSD: post-traumatic stress disorder; R: retrospective; ST: Schirmer test; TBUT: tear film break-up time; TD: thyroid disease; TFBT: tear film breakup time; VDT: visual display terminal

### Study characteristics

Of 48 included studies, 39 studies were designed as cross-sectional, 7 studies were designed as retrospective, and 2 studies designed as prospective. A total of 493,630 individuals were included, and the sample size ranged from 86 to 102,582. The mean age of included individuals ranged from 10.9 to 82.2. Twenty-nine studies were performed in Eastern countries, with the remaining 19 studies conducted in Western countries. Thirty-nine studies were population based. The remaining 9 studies were hospital based. The DES definition based on questionnaire were reported in 33 studies, 10 studies used TBUT, ST, or FSS defined DES, 3 studies applied ICD9 code and the remaining 2 studies used clinician-diagnosed defined DES. Study quality was assessed using the NOS; 11 studies had 8 stars, 18 had 7 stars, and the remaining 19 had 6 stars (S1 Table). The quality of included studies mainly affect by the representativeness of the exposed cohort, and comparability on the basis of the design or analysis.

### Meta-analysis

#### Demographic factors

**Fig 2:**
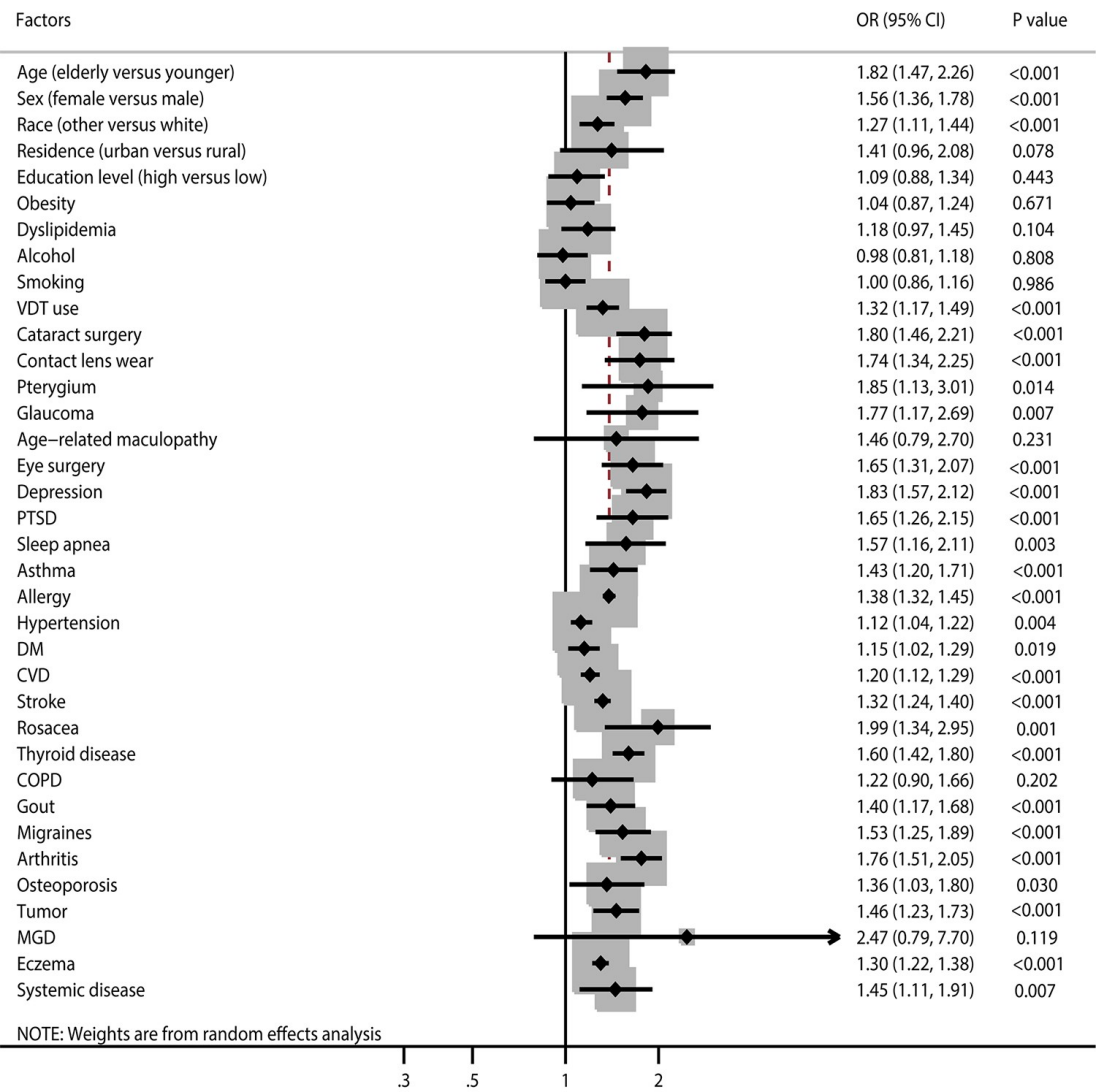
Summary results of risk factors for dry eye syndrome.

The number of studies that reported on the association of age, sex, and race as risk factors for DES was 15, 29, and 5, respectively (Fig 2 and S2 File). We noted that older adults (OR: 1.82; 95%CI: 1.47–2.26; *P*<0.001), females (OR: 1.56; 95%CI: 1.36–1.78; *P*<0.001), and those of other race (OR: 1.27; 95%CI: 1.11–1.44; *P*<0.001) had an increased risk of DES. There was significant heterogeneity for age (*I*^*2*^ = 96.0%; *P*<0.001), sex (*I*^*2*^ = 95.0%; *P*<0.001), and race (*I*^*2*^ = 52.1%; *P* = 0.080). Sensitivity analysis indicated these pooled conclusions were robust and not altered by sequentially excluding individual studies (S3 File). The results of subgroup analyses were consistent with overall analysis when stratified according to the region (Table 2). There were no significant publication biases for age (*P-*value for Egger: 0.175; *P*-value for Begg: 1.000), sex (*P*-value for Egger: 0.417; *P*-value for Begg: 0.253), and race (*P*-value for Egger: 0.174; *P*-value for Begg: 0.806) regarding risk for DES (S4 File).

**Table 2:** Subgroup analyses according to region.

Factors	Subgroup	OR and 95%CI	*P* value	*I*^*2*^ (%)	*P*_*heterogeneity*_	*P* value between subgroups
Age (elderly versus younger)	Eastern countries	1.78 (1.46–2.19)	< 0.001	89.4	< 0.001	< 0.001
	Western countries	2.04 (1.05–3.97)	0.036	98.7	< 0.001	
Sex (female versus male)	Eastern countries	1.53 (1.36–1.72)	< 0.001	84.0	< 0.001	< 0.001
	Western countries	1.52 (1.20–1.92)	< 0.001	95.8	< 0.001	
Race (other versus white)	Eastern countries	-	-	-	-	-
	Western countries	1.27 (1.11–1.44)	< 0.001	52.1	0.080	
Residence (urban versus rural)	Eastern countries	1.41 (0.96–2.08)	0.078	87.8	< 0.001	-
	Western countries	-	-	-	-	
Education level (high versus low)	Eastern countries	1.28 (1.01–1.63)	0.041	60.2	0.057	0.007
	Western countries	0.86 (0.56–1.33)	0.505	80.7	0.001	
Obesity	Eastern countries	1.02 (0.83–1.25)	0.866	2.1	0.360	0.685
	Western countries	1.11 (0.77–1.60)	0.576	-	-	
Dyslipidemia	Eastern countries	1.35 (1.01–1.80)	0.046	94.7	< 0.001	< 0.001
	Western countries	1.05 (0.82–1.35)	0.676	77.2	0.004	
Alcohol	Eastern countries	1.04 (0.90–1.20)	0.589	0.0	0.429	0.177
	Western countries	0.92 (0.64–1.32)	0.641	76.1	<0.001	
Smoking	Eastern countries	0.96 (0.81–1.15)	0.668	65.2	< 0.001	0.046
	Western countries	1.09 (0.82–1.45)	0.554	60.0	0.020	
VDT use	Eastern countries	1.33 (1.17–1.53)	< 0.001	85.5	< 0.001	0.436
	Western countries	1.33 (1.06–1.68)	0.015	0.0	0.457	
Cataract surgery	Eastern countries	2.16 (1.62–2.89)	< 0.001	0.0	0.792	0.561
	Western countries	1.69 (1.28–2.21)	< 0.001	76.0	0.002	
Contact lens wear	Eastern countries	2.01 (1.48–2.71)	<0.001	72.8	<0.001	0.003
	Western countries	1.41 (0.93–2.14)	0.105	97.1	<0.001	
Pterygium	Eastern countries	1.85 (1.13–3.01)	0.014	89.0	< 0.001	-
	Western countries	-	-	-	-	
Glaucoma	Eastern countries	2.15 (1.29–3.58)	0.003	26.1	0.255	0.516
	Western countries	1.57 (0.92–2.68)	0.098	96.5	< 0.001	
Age-related maculopathy	Eastern countries	0.31 (0.07–1.35)	0.118	-	-	0.007
	Western countries	1.91 (1.21–3.01)	0.005	62.9	0.067	
Eye surgery	Eastern countries	1.62 (1.23–2.14)	0.001	93.6	<0.001	<0.001
	Western countries	1.82 (1.39–2.37)	< 0.001	32.1	0.229	
Depression	Eastern countries	2.12 (1.95–2.32)	< 0.001	0.0	0.876	< 0.001
	Western countries	1.66 (1.43–1.93)	< 0.001	67.0	0.010	
PTSD	Eastern countries	1.45 (1.04–2.01)	0.027	-	-	0.121
	Western countries	1.71 (1.19–2.46)	0.004	53.0	0.119	
Sleep apnea	Eastern countries	1.22 (1.11–1.35)	< 0.001	4.5	0.370	< 0.001
	Western countries	2.17 (1.95–2.41)	< 0.001	0.0	0.749	
Asthma	Eastern countries	1.19 (0.98–1.45)	0.076	29.0	0.235	< 0.001
	Western countries	1.62 (1.49–1.77)	< 0.001	0.0	0.869	
Allergy	Eastern countries	-	-	-	-	-
	Western countries	1.38 (1.32–1.45)	< 0.001	0.0	0.418	
Hypertension	Eastern countries	1.06 (0.95–1.17)	0.306	63.7	0.001	0.005
	Western countries	1.27 (1.14–1.41)	< 0.001	24.8	0.231	
DM	Eastern countries	1.20 (1.06–1.37)	0.005	79.0	< 0.001	< 0.001
	Western countries	1.08 (0.87–1.34)	0.460	88.5	< 0.001	
CVD	Eastern countries	1.26 (1.15–1.39)	< 0.001	0.0	0.753	0.084
	Western countries	1.15 (1.00–1.32)	0.049	18.5	0.293	
Stroke	Eastern countries	1.31 (1.22–1.41)	< 0.001	0.0	0.978	0.667
	Western countries	1.35 (1.20–1.51)	< 0.001	0.0	0.589	
Rosacea	Eastern countries	3.75 (1.97–7.12)	< 0.001	-	-	0.032
	Western countries	1.74 (1.20–2.52)	0.004	53.1	0.094	
Thyroid disease	Eastern countries	1.57 (1.29–1.91)	< 0.001	86.0	<0.001	0.752
	Western countries	1.64 (1.45–1.84)	< 0.001	26.9	0.223	
COPD	Eastern countries	1.06 (0.84–1.34)	0.625	-	-	0.006
	Western countries	1.37 (1.00–1.89)	0.051	23.2	0.272	
Gout	Eastern countries	1.56 (0.70–3.49)	0.275	83.3	0.014	0.175
	Western countries	1.34 (1.17–1.53)	< 0.001	0.0	0.860	
Migraines	Eastern countries	1.76 (1.57–1.98)	< 0.001	-	-	< 0.001
	Western countries	1.41 (1.19–1.68)	< 0.001	54.2	0.088	
Arthritis	Eastern countries	1.74 (1.31–2.29)	< 0.001	95.6	< 0.001	0.776
	Western countries	1.80 (1.57–2.07)	< 0.001	74.7	0.001	
Osteoporosis	Eastern countries	0.81 (0.51–1.29)	0.377	-	-	0.004
	Western countries	1.53 (1.21–1.93)	< 0.001	75.8	0.016	
Tumor	Eastern countries	2.27 (0.83–6.22)	0.111	94.7	< 0.001	0.339
	Western countries	1.33 (1.17–1.50)	<0.001	39.5	0.175	

The number of studies reporting an association of residence, education level, obesity, and dyslipidemia regarding the risk of DES were 4, 8, 4, and 7, respectively (Fig 2 and S2 File). We noted that residence (urban versus rural) (OR: 1.41; 95%CI: 0.96–2.08; *P* = 0.078), education level (high versus low) (OR: 1.09; 95%CI: 0.88–1.34; *P* = 0.443), obesity (OR: 1.04; 95%CI: 0.87–1.24; *P* = 0.671), and dyslipidemia (OR: 1.18; 95%CI: 0.97–1.45; *P* = 0.104) were not associated with increased risk for DES. There was significant heterogeneity for residence (*I*^*2*^ = 87.8%; *P*<0.001), education level (*I*^*2*^ = 76.9%; *P*<0.001), and dyslipidemia (*I*^*2*^ = 92.9%; *P*<0.001), while there was no evidence of heterogeneity for obesity (*I*^*2*^ = 0.0%; *P* = 0.530). Sensitivity analyses indicated that residence, education level, and dyslipidemia might be associated with an elevated risk of DES, while the association between obesity and DES persisted (S3 File). Subgroup analyses demonstrated that education level and dyslipidemia were associated with an increased risk of DES when pooling studies conducted in Eastern countries (Table 2). No significant publication bias for residence (*P*-value for Egger: 0.875; *P*-value for Begg: 0.734), education level (*P*-value for Egger: 0.985; *P*-value for Begg: 0.902), and obesity (*P*-value for Egger: 0.638; *P*-value for Begg: 0.308) with the risk of DES was noted, whereas potential significant publication bias for dyslipidemia (*P*-value for Egger: 0.037; *P*-value for Begg: 1.000) with the risk of DES was seen (S4 File).

The number of studies reporting an association of alcohol, smoking, and VDT use with the risk of DES was 15, 22, and 14, respectively (Fig 2 and S2 File). We noted that alcohol intake (OR: 0.98; 95%CI: 0.81–1.18; *P* = 0.808) and current smoking (OR: 1.00; 95%CI: 0.86–1.16; *P* = 0.986) were not associated with risk for DES, while VDT use was associated with an increased risk of DES (OR: 1.32; 95%CI: 1.17–1.49; *P*<0.001). There was significant heterogeneity for alcohol (*I*^*2*^ = 62.2%; *P* = 0.001), smoking (*I*^*2*^ = 64.6%; *P*<0.001), and VDT use (*I*^*2*^ = 80.1%; *P*<0.001). Sensitivity analysis indicated that alcohol intake might play an important role in the risk of DES, while the pooled results for the associations of smoking and VDT use with the risk of DES were robust (S3 File). The results of subgroup analyses were consistent with the overall analysis (Table 2). No significant publication bias for smoking (*P*-value for Egger: 0.569; *P*-value for Begg: 0.822) and VDT use (*P*-value for Egger: 0.370; *P* value for Begg: 0.827) with the risk of DES was found, whereas potential significant publication bias for alcohol (*P*-value for Egger: 0.032; *P*-value for Begg: 0.921) with the risk of DES was noted (S4 File).

#### Clinical characteristics

The number of studies that reported on the association of cataract surgery, contact lens wear, pterygium, glaucoma, age-related maculopathy, and eye surgery with the risk of DES were 7, 17, 4, 9, 3, and 8, respectively (Fig 2 and S2 File). We noted that cataract surgery (OR: 1.80; 95%CI: 1.46–2.21; *P*<0.001), contact lens wear (OR: 1.74; 95%CI: 1.34–2.25; *P*<0.001), pterygium (OR: 1.85; 95%CI: 1.13–3.01; *P* = 0.014), glaucoma (OR: 1.77; 95%CI: 1.17–2.69; *P* = 0.007), and eye surgery (OR: 1.65; 95%CI: 1.31–2.07; *P*<0.001) were associated with an increased risk of DES, while age-related maculopathy was not associated with risk of DES (OR: 1.46; 95%CI: 0.79–2.70; *P* = 0.231). Significant heterogeneity was noted for cataract surgery (*I*^*2*^ = 64.8%; *P*<0.001), contact lens wear (*I*^*2*^ = 93.5%; *P*<0.001), pterygium (*I*^*2*^ = 89.0%; *P*<0.001), glaucoma (*I*^*2*^ = 93.4%; *P*<0.001), age-related maculopathy (*I*^*2*^ = 76.5%; *P* = 0.005), and eye surgery (*I*^*2*^ = 94.0%; *P*<0.001) with the risk of DES. Sensitivity analyses indicated that the pooled results for the association of cataract surgery, contact lens wear, pterygium, glaucoma, and eye surgery with the risk of DES persisted, whereas age-related maculopathy might be associated with the risk of DES (S3 File). Although most results in the subgroup analyses were consistent with the overall analysis, we noted that contact lens wear and glaucoma were not associated with the risk of DES when pooling studies performed in Western countries. Moreover, age-related maculopathy was associated with an increased risk of DES when pooling studies conducted in Western countries (Table 2). There was no significant publication bias for the association of cataract surgery (*P*-value for Egger: 0.194; *P*-value for Begg: 0.548), contact lens wear (*P*-value for Egger: 0.791; *P*-value for Begg: 0.387), pterygium (*P-*value for Egger: 0.681; *P*-value for Begg: 0.734), glaucoma (*P*-value for Egger: 0.950; *P*-value for Begg: 0.917), and eye surgery (*P*-value for Egger: 0.760; *P*-value for Begg: 0.266) with the risk of DES, while potential significant publication bias was noted for age-related maculopathy (*P*-value for Egger: 0.017; *P*-value for Begg: 0.308) with the risk of DES (S4 File).

#### Comorbidities

The number of studies that reported on the association of depression, PTSD, sleep apnea, asthma, and allergy with the risk of DES were 9, 4, 7, 6, and 6, respectively (Fig 2 and S2 File). We noted that depression (OR: 1.83; 95%CI: 1.57–2.12; *P*<0.001), PTSD (OR: 1.65; 95%CI: 1.26–2.15; *P*<0.001), sleep apnea (OR: 1.57; 95%CI: 1.16–2.11; *P* = 0.003), asthma (OR: 1.43; 95%CI: 1.20–1.71; *P*<0.001), and allergy (OR: 1.38; 95%CI: 1.32–1.45; *P*<0.001) were associated with an increased risk of DES. There was significant heterogeneity for depression (*I*^*2*^ = 80.7%; *P*<0.001), PTSD (*I*^*2*^ = 55.0%; *P* = 0.083), sleep apnea (*I*^*2*^ = 91.5%; *P*<0.001), and asthma (*I*^*2*^ = 76.5%; *P* = 0.001), while no evidence of heterogeneity for allergy was observed (*I*^*2*^ = 0.0%; *P* = 0.418). Sensitivity analyses indicated that pooled conclusions for the association of depression, PTSD, sleep apnea, asthma, and allergy with the risk of DES were stable after sequentially removing individual studies (S3 File). The results of subgroup analyses were consistent with overall analysis, except that asthma was not associated with the risk of DES if pooled studies were performed in Eastern countries (Table 2). No significant publication bias for the role of depression (*P*-value for Egger: 0.679; *P*-value for Begg: 0.348), PTSD (*P*-value for Egger: 0.415; *P*-value for Begg: 0.734), sleep apnea (*P*-value for Egger: 0.959; *P*-value for Begg: 0.764), asthma (*P*-value for Egger: 0.949; *P*-value for Begg: 1.000), and allergy (*P*-value for Egger: 0.189; *P*-value for Begg: 0.707) with DES were observed (S4 File).

The number of studies reporting on the association of hypertension, DM, CVD, stroke, rosacea, thyroid disease, and COPD with the risk of DES were 21, 24, 8, 7, 5, 14, and 4, respectively (Fig 2 and S2 File). We noted that hypertension (OR: 1.12; 95%CI: 1.04–1.22; *P* = 0.004), DM (OR: 1.15; 95%CI: 1.02–1.29; *P* = 0.019), CVD (OR: 1.20; 95%CI: 1.12–1.29; *P*<0.001), stroke (OR: 1.32; 95%CI: 1.24–1.40; *P*<0.001), rosacea (OR: 1.99; 95%CI: 1.34–2.95; *P* = 0.001), and thyroid disease (OR: 1.60; 95%CI: 1.42–1.80; *P*<0.001) were associated with an increased risk of DES, while COPD was not associated with risk of DES (OR: 1.22; 95%CI: 10.90–1.66; *P* = 0.202). There was significant heterogeneity for hypertension (*I*^*2*^ = 60.2%; *P*<0.001), DM (*I*^*2*^ = 86.7%; *P*<0.001), rosacea (*I*^*2*^ = 63.6%; *P* = 0.027), thyroid disease (*I*^*2*^ = 74.6%; *P*<0.001), and COPD (*I*^*2*^ = 70.6%; *P* = 0.017), while no significant heterogeneity was observed for CVD (*I*^*2*^ = 4.8%; *P* = 0.393) and stroke (*I*^*2*^ = 0.0%; *P* = 0.964). The pooled conclusions for the association of hypertension, CVD, stroke, rosacea, and thyroid disease with the risk of DES were stable, while the conclusions for DM and COPD with DES were variable (S3 File). Although the results of subgroup analyses were consistent with the overall analysis in most subsets, we noted that hypertension was not related to DES if pooling in Eastern country studies, while DM was not associated with the risk of DES if pooled studies were performed in Western countries (Table 2). There was no significant publication bias for hypertension (*P*-value for Egger: 0.331; *P*-value for Begg: 0.928), DM (*P*-value for Egger: 0.765; *P*-value for Begg: 0.862), CVD (*P*-value for Egger: 0.357; *P*-value for Begg: 0.711), stroke (*P*-value for Egger: 0.485; *P*-value for Begg: 0.368), rosacea (*P*-value for Egger: 0.759; *P*-value for Begg: 0.806), thyroid disease (*P*-value for Egger: 0.996; *P*-value for Begg: 0.228), and COPD (*P*-value for Egger: 0.267; *P*-value for Begg: 1.000) (S4 File).

The number of studies reporting on the association of gout, migraines, arthritis, osteoporosis, tumor, MGD, eczema, and systemic disease with the risk of DES was 6, 5, 13, 4, 6, 3, 3, and 3, respectively (Fig 2 and S2 File). We noted that gout (OR: 1.40; 95%CI: 1.17–1.68; *P*<0.001), migraines (OR: 1.53; 95%CI: 1.25–1.89; *P*<0.001), arthritis (OR: 1.76; 95%CI: 1.51–2.05; *P*<0.001), osteoporosis (OR: 1.36; 95%CI: 1.03–1.80; *P* = 0.030), tumor (OR: 1.46; 95%CI: 1.23–1.76; *P*<0.001), eczema (OR: 1.30; 95%CI: 1.22–1.38; *P*<0.001), and systemic disease (OR: 1.45; 95%CI: 1.11–1.91; *P* = 0.007) were associated with an increased risk of DES, while MGD was not associated with risk of DES (OR: 2.47; 95%CI: 0.79–7.70; *P* = 0.119). There was significant heterogeneity for migraines (*I*^*2*^ = 86.4%; *P*<0.001), arthritis (*I*^*2*^ = 92.4%; *P*<0.001), osteoporosis (*I*^*2*^ = 82.1%; *P* = 0.001), tumor (*I*^*2*^ = 79.9%; *P*<0.001), and MGD (*I*^*2*^ = 85.2%; *P* = 0.001), while no significant heterogeneity for gout (*I*^*2*^ = 41.8%; *P* = 0.126), eczema (*I*^*2*^ = 0.0%; *P* = 0.609), and systemic disease (*I*^*2*^ = 0.0%; *P* = 0.007) was observed. The pooled conclusions for the association of gout, migraines, arthritis, osteoporosis, and tumor with the risk of DES were robust after sequentially removing individual studies (S3 File). Although the results of subgroup analyses were consistent with the overall analysis in most subsets, gout, osteoporosis, and tumor were not associated with risk of DES if pooled studies were performed in Eastern countries. There was no significant publication bias for gout (*P*-value for Egger: 0.902; *P*-value for Begg: 0.707), migraines (*P*-value for Egger: 0.249; *P*-value for Begg: 0.806), arthritis (*P*-value for Egger: 0.169; *P*-value for Begg: 0.360), osteoporosis (*P*-value for Egger: 0.137; *P*-value for Begg: 0.308), and tumor (*P*-value for Egger: 0.721; *P*-value for Begg: 1.000) (S4 File).

## Discussion

This systematic review and meta-analysis was based on published observational studies explored potential risk factors for DES and included 493,630 individuals from 48 studies. We found that risk factors for DES included older age, female sex, other race, VDT use, cataract surgery, contact lens wear, pterygium, glaucoma, eye surgery, depression, PTSD, sleep apnea, asthma, allergy, hypertension, DM, CVD, stroke, rosacea, thyroid disease, gout, migraines, arthritis, osteoporosis, tumor, eczema, and systemic disease. Moreover, country of origin could affect association for age, sex, education level, dyslipidemia, smoking, contact lens wear, age-related maculopathy, eye surgery, depression, sleep apnea, asthma, hypertension, DM, rosacea, COPD, migraines, and osteoporosis regarding the risk of DES.

This current study primarily identified potential risk factors for DES, although several factors have already been demonstrated in individual studies. Prior studies have demonstrated that a 5-year incidence of dry eye rises from 10.7% to 17.9% alongside increasing age [72]. A potential reason could be the reduction of tear secretion with biological aging [2, 73]. Moreover, the sex difference in DES could be explained by various hormonal effects on the ocular surface and lacrimal gland [8]. The potential impact for VDT use could be due to increasing rates of incomplete blinks and accelerated evaporation of the tear film [74]. The increased risk of DES after cataract surgery could be explained by cataract surgery inducing tear film dysfunction [75]. The role of contact lens wear on DES could be explained in that placing a lens on the eye could cause disturbance of the tear film [76]. DES could be considered as a precipitating factor of primary pterygium [77]. The treatment of glaucoma could alter the surface of the eye through disturbing tear secretion, which could affect the progression of DES [78]. Studies have already found that open eye surgery could affect altered tear secretion in nearly 91% of patients, thus playing an important role in the risk of DES [79]. The potential role of depression and PTSD could be explained by the dysregulation of neuropeptides coupled with serotonin in human tears and serotonin receptors in human conjunctivae [80]. Sleep apnea is significantly associated with neuropathic pain, which could induce the progression of dry eye syndrome [81]. The role of asthma and allergy on the risk of DES could be explained by antihistaminic and anti-inflammatory agents used for asthma and allergy treatment, which could potentially cause an elevated risk of DES [82].

This study found that hypertension and DM were associated with an increased risk of DES, which was consistent with the results of a prior meta-analysis [83]. A potential reason for this could be hypertension was not direct affect the risk of DES, while the use of anti-hypertensive medication could increase the risk of DES [33]. In addition, the risk of DES were not increased in hypertensive patients treated with anti-hypertensive medications, such as Angiotension Converting Enzyme inhibitors might play a protective role on the risk of DES [34]. Moreover, DM could induce a decrease in corneal sensation and tear production, impaired metabolic activity, and loss of cytoskeletal structure, all of which could affect the progression of DES [84]. The underlying therapies for CVD, stroke, and tumor could be regarded as disposing of factors for DES [25]. Rosacea is a well known risk factor for DES due to is pro-inflammatory effects that induce meibomian gland dysfunction and evaporative DES [85]. Studies have already found that thyroid disease is significantly related to ocular surface damage, eyelid retraction/impaired Bell’s phenomenon, and reduced tear production [86]. Gout was associated with the tophaceous deposits in different locations of the eye, including eyelids, conjunctiva, cornea, iris, sclera, and orbit, a similar reason could explain the role of arthritis on DES [87]. The role of migraines on DES could be explained by an inflammatory status in migraine patients potentially activating inflammation in the eyes [88]. The inflammation and hormone imbalance caused by osteoporosis could explain an elevated risk of DES [89]. The treatment for eczema and systemic disease could cause an elevated risk of DES [90].

Our study found that potential associations for age, sex, education level, dyslipidemia, smoking, contact lens wear, age-related maculopathy, eye surgery, depression, sleep apnea, asthma, hypertension, DM, rosacea, COPD, migraines, and osteoporosis with the risk of DES could be affected by country of origin. The disease distribution for DES is different in Eastern and Western countries, and the health policy in various countries could further affect the progression of DES. Moreover, environmental, dietary, and lifestyle factors among various countries differ, which could affect the progression of DES [91, 92].

Several shortcomings of this study should be acknowledged. First, this study contained cross-sectional, retrospective, and prospective observational studies, and the causality relationships between risk factors and DES could not available. Second, the heterogeneity for most risk factors was substantial, which was not fully explained by sensitivity and subgroup analyses. Third, the comorbidity and underlying therapies for individuals were not fully adjusted, which could affect the progression of DES. Fourth, the cutoff value for age, and definition for systemic disease, eye surgery, and DES are different across included studies, which could induce potential uncontrolled biases. Fifth, the climate type could affect the progression of DES, and nearly all of included studies did not address the climate type. Sixth, the analysis based on published articles, the gray literature and unpublished data were not available, and the publication bias was inevitable. Seventh, the analysis using the pooled data, and the detailed analyses were restricted. Finally, this study was not registered in PROSPERO, and the transparency was restricted.

## Conclusions

This study identified comprehensive risk factors for DES, including older age, female sex, other race, VDT use, cataract surgery, contact lens wear, pterygium, glaucoma, eye surgery, depression, PTSD, sleep apnea, asthma, allergy, hypertension, DM, CVD, stroke, rosacea, thyroid disease, gout, migraines, arthritis, osteoporosis, tumor, eczema, and systemic disease. Further large-scale prospective cohort studies should be performed to verify the results of this study.

## Supporting information

S1 Checklist PRISMA 2020 checklist. (PDF)

S1 Table Quality scores of prospective cohort studies using Newcastle-Ottawa Scale. (DOCX)

S1 File Search strategy in PubMed. (DOCX)

S2 File Forest plots for the risk factors of dry eye syndrome. (DOCX)

S3 File Sensitivity analyses for the risk factors of dry eye syndrome. (DOCX)

S4 File Funnel plots for the risk factors of dry eye syndrome. (DOCX)

## Abbreviations

CI: confidence interval
COPD: chronic obstructive pulmonary disease
CVD: cardiovascular disease
DES: dry eye syndrome
DM: diabetes mellitus
HR: hazard ratio
MGD: meibomian gland dysfunction
NOS: Newcastle-Ottawa Scale
OR: odds ratio
PTSD: post-traumatic stress disorder
RR: relative risk
VDT: visual display terminal

## References

[1] Craig JP, Nichols KK, Akpek EK, Caffery B, Dua HS, Joo CK, et al. TFOS DEWS II Definition and Classification Report. Ocul Surf. 2017;15:276–283. doi: 10.1016/j.jtos.2017.05.008 28736335

[2] Gayton JL. Etiology, prevalence, and treatment of dry eye disease. Clin Ophthalmol. 2009;3:405–412. doi: 10.2147/opth.s5555 19688028 PMC2720680

[3] Uchino M, Schaumberg D. Dry Eye Disease: Impact on Quality of Life and Vision. Curr Ophthalmol Rep 2013;1:51–7. doi: 10.1007/s40135-013-0009-1 23710423 PMC3660735

[4] Findlay Q, Reid K. Dry eye disease: when to treat and when to refer. Aust Prescr 2018; 41:160–3. doi: 10.18773/austprescr.2018.048 30410213 PMC6202299

[5] Javadi MA, Feizi S. Dry eye syndrome. J Ophthalmic Vis Res 2011;6:192–8. 22454735 PMC3306104

[6] Jain S, Bhavsar A, Bhavsar S. A review on recent advances in dry eye: Pathogenesis and management. Oman J Opthalmol 2011;4:50. doi: 10.4103/0974-620X.83653 21897618 PMC3160069

[7] Dana R, Bradley JL, Guerin A, Pivneva I, Stillman IÖ, Evans AM, et al. Estimated Prevalence and Incidence of Dry Eye Disease Based on Coding Analysis of a Large, All-age United States Health Care System. Am J Ophthalmol. 2019;202:47–54. doi: 10.1016/j.ajo.2019.01.026 30721689

[8] Song P, Xia W, Wang M, Chang X, Wang J, Jin S, et al. Variations of dry eye disease prevalence by age, sex and geographic characteristics in China: a systematic review and meta-analysis. J Glob Health. 2018;8:020503. doi: 10.7189/jogh.08.020503 30206477 PMC6122008

[9] The epidemiology of dry eye disease: report of the Epidemiology Subcommittee of the International Dry Eye WorkShop (2007). Ocul Surf 2007;5:93–107. doi: 10.1016/s1542-0124(12)70082-4 17508117

[10] Courtin R, Pereira B, Naughton G, Chamoux A, Chiambaretta F, Lanhers C, et al. Prevalence of dry eye disease in visual display terminal workers: a systematic review and meta-analysis. BMJ Open. 2016;6:e009675. doi: 10.1136/bmjopen-2015-009675 26769784 PMC4735196

[11] Ahn J, Ryu SJ, Song J, Kim HR. Shift Work and Dry Eye Disease in the Korean Working Population: A Population-Based Cross-Sectional Study. Int J Environ Res Public Health. 2021;18:5492. doi: 10.3390/ijerph18105492 34065509 PMC8161339

[12] Liu J, Dong Y, Wang Y. Vitamin D deficiency is associated with dry eye syndrome: a systematic review and meta-analysis. Acta Ophthalmol. 2020;98:749–754. doi: 10.1111/aos.14470 32421222

[13] Yoo TK, Oh E. Diabetes mellitus is associated with dry eye syndrome: a meta-analysis. Int Ophthalmol. 2019;39:2611–2620. doi: 10.1007/s10792-019-01110-y 31065905

[14] Moher D, Liberati A, Tetzlaff J, Altman DG; PRISMA Group. Preferred reporting items for systematic reviews and meta-analyses: the PRISMA statement. PLoS Med. 2009;6: e1000097. doi: 10.1371/journal.pmed.1000097 19621072 PMC2707599

[15] Song P, Rudan D, Zhu Y, Fowkes FJI, Rahimi K, Fowkes FGR, et al. Global, regional, and national prevalence and risk factors for peripheral artery disease in 2015: an updated systematic review and analysis. Lancet Glob Health. 2019;7:e1020–e1030 doi: 10.1016/S2214-109X(19)30255-4 31303293

[16] Wells G, Shea B, O’Connell D. The Newcastle-Ottawa Scale (NOS) for assessing the quality of nonrandomised studies in meta-analyses. Ottawa (ON): Ottawa Hospital Research Institute 2009. Available: http://www.ohri.ca/programs/clinical_epidemiology /oxford.htm.

[17] DerSimonian R, Laird N. Meta-analysis in clinical trials. Control Clin Trials. 1986; 7: 177–88. doi: 10.1016/0197-2456(86)90046-2 3802833

[18] Ades AE, Lu G, Higgins JP. The interpretation of random-effects metaanalysis in decision models. Med Decis Making. 2005; 25: 646–54. doi: 10.1177/0272989X05282643 16282215

[19] Deeks JJ, Higgins JPT, Altman DG. Analyzing data and undertaking meta-analyses. In: Higgins J, Green S, eds. Cochrane Handbook for Systematic Reviews of Interventions 5.0.1. Oxford, UK: The Cochrane Collaboration: 2008; chap 9.

[20] Higgins JP, Thompson SG, Deeks JJ, Altman DG. Measuring inconsistency in meta-analyses. BMJ. 2003;327:557–60. doi: 10.1136/bmj.327.7414.557 12958120 PMC192859

[21] Tobias A. Assessing the influence of a single study in meta-analysis. Stata Tech Bull. 1999;47:15–7.

[22] Altman DG, Bland JM. Interaction revisited: the difference between two estimates. BMJ. 2003; 326: 219. doi: 10.1136/bmj.326.7382.219 12543843 PMC1125071

[23] Egger M, Davey Smith G, Schneider M, Minder C. Bias in meta-analysis detected by a simple, graphical test. BMJ. 1997;315:629–34. doi: 10.1136/bmj.315.7109.629 9310563 PMC2127453

[24] Begg CB, Mazumdar M. Operating characteristics of a rank correlation test for publication bias. Biometrics. 1994; 50: 1088–1101. 7786990

[25] Moss SE, Klein R, Klein BE. Prevalence of and risk factors for dry eye syndrome. Arch Ophthalmol. 2000;118:1264–8. doi: 10.1001/archopht.118.9.1264 10980773

[26] Lee AJ, Lee J, Saw SM, Gazzard G, Koh D, Widjaja D, et al. Prevalence and risk factors associated with dry eye symptoms: a population based study in Indonesia. Br J Ophthalmol. 2002;86:1347–51. doi: 10.1136/bjo.86.12.1347 12446361 PMC1771386

[27] Chia EM, Mitchell P, Rochtchina E, Lee AJ, Maroun R, Wang JJ. Prevalence and associations of dry eye syndrome in an older population: the Blue Mountains Eye Study. Clin Exp Ophthalmol. 2003;31:229–32. doi: 10.1046/j.1442-9071.2003.00634.x 12786773

[28] Sahai A, Malik P. Dry eye: prevalence and attributable risk factors in a hospital-based population. Indian J Ophthalmol. 2005;53:87–91. doi: 10.4103/0301-4738.16170 15976462

[29] Nichols JJ, Sinnott LT. Tear film, contact lens, and patient-related factors associated with contact lens-related dry eye. Invest Ophthalmol Vis Sci. 2006;47:1319–28. doi: 10.1167/iovs.05-1392 16565363

[30] Uchino M, Schaumberg DA, Dogru M, Uchino Y, Fukagawa K, Shimmura S, et al. Prevalence of dry eye disease among Japanese visual display terminal users. Ophthalmology. 2008;115:1982–8. doi: 10.1016/j.ophtha.2008.06.022 18708259

[31] Lu P, Chen X, Liu X, Yu L, Kang Y, Xie Q, et al. Dry eye syndrome in elderly Tibetans at high altitude: a population-based study in China. Cornea. 2008;27:545–51. doi: 10.1097/ICO.0b013e318165b1b7 18520503

[32] Schaumberg DA, Dana R, Buring JE, Sullivan DA. Prevalence of dry eye disease among US men: estimates from the Physicians’ Health Studies. Arch Ophthalmol. 2009;127:763–8. doi: 10.1001/archophthalmol.2009.103 19506195 PMC2836718

[33] Viso E, Rodriguez-Ares MT, Gude F. Prevalence of and associated factors for dry eye in a Spanish adult population (the Salnes Eye Study). Ophthalmic Epidemiol. 2009;16: 15–21. doi: 10.1080/09286580802228509 19191177

[34] Jie Y, Xu L, Wu YY, Jonas JB. Prevalence of dry eye among adult Chinese in the Beijing Eye Study. Eye (Lond). 2009;23:688–93. doi: 10.1038/sj.eye.6703101 18309341

[35] Guo B, Lu P, Chen X, Zhang W, Chen R. Prevalence of dry eye disease in Mongolians at high altitude in China: the Henan eye study. Ophthalmic Epidemiol. 2010;17:234–41. doi: 10.3109/09286586.2010.498659 20642346

[36] Kim KW, Han SB, Han ER, Woo SJ, Lee JJ, Yoon JC, et al. Association between depression and dry eye disease in an elderly population. Invest Ophthalmol Vis Sci. 2011;52: 7954–8. doi: 10.1167/iovs.11-8050 21896858

[37] Uchino M, Nishiwaki Y, Michikawa T, Shirakawa K, Kuwahara E, Yamada M, et al. Prevalence and risk factors of dry eye disease in Japan: Koumi study. Ophthalmology. 2011; 118:2361–7. doi: 10.1016/j.ophtha.2011.05.029 21889799

[38] Galor A, Feuer W, Lee DJ, Florez H, Carter D, Pouyeh B, et al. Prevalence and risk factors of dry eye syndrome in a United States veterans affairs population. Am J Ophthalmol. 2011;152:377–384.e2. doi: 10.1016/j.ajo.2011.02.026 21684522 PMC4113967

[39] Zhang Y, Chen H, Wu X. Prevalence and risk factors associated with dry eye syndrome among senior high school students in a county of Shandong Province, China. Ophthalmic Epidemiol. 2012;19:226–30. doi: 10.3109/09286586.2012.670742 22650150

[40] Wang TJ, Wang IJ, Hu CC, Lin HC. Comorbidities of dry eye disease: a nationwide population-based study. Acta Ophthalmol. 2012;90:663–8. doi: 10.1111/j.1755-3768.2010.01993.x 20809911

[41] Uchino M, Yokoi N, Uchino Y, Dogru M, Kawashima M, Komuro A, et al. Prevalence of dry eye disease and its risk factors in visual display terminal users: the Osaka study. Am J Ophthalmol. 2013;156:759–66. doi: 10.1016/j.ajo.2013.05.040 23891330

[42] Vehof J, Kozareva D, Hysi PG, Hammond CJ. Prevalence and risk factors of dry eye disease in a British female cohort. Br J Ophthalmol. 2014;98:1712–7. doi: 10.1136/bjophthalmol-2014-305201 25185440

[43] Ahn JM, Lee SH, Rim TH, Park RJ, Yang HS, Kim TI, et al. Prevalence of and risk factors associated with dry eye: the Korea National Health and Nutrition Examination Survey 2010–2011. Am J Ophthalmol. 2014;158:1205–1214.e7. doi: 10.1016/j.ajo.2014.08.021 25149910

[44] Moon JH, Lee MY, Moon NJ. Association between video display terminal use and dry eye disease in school children. J Pediatr Ophthalmol Strabismus. 2014;51:87–92. doi: 10.3928/01913913-20140128-01 24495620

[45] Paulsen AJ, Cruickshanks KJ, Fischer ME, Huang GH, Klein BE, Klein R, et al. Dry eye in the beaver dam offspring study: prevalence, risk factors, and health-related quality of life. Am J Ophthalmol. 2014;157:799–806. doi: 10.1016/j.ajo.2013.12.023 24388838 PMC3995164

[46] Chen HY, Lin CL, Tsai YY, Kao CH. Association between Glaucoma Medication Usage and Dry Eye in Taiwan. Optom Vis Sci. 2015;92:e227–32. doi: 10.1097/OPX.0000000000000667 26192153

[47] Yang WJ, Yang YN, Cao J, Man ZH, Yuan J, Xiao X, et al. Risk Factors for Dry Eye Syndrome: A Retrospective Case-Control Study. Optom Vis Sci. 2015;92:e199–205. doi: 10.1097/OPX.0000000000000541 25756335

[48] Tan LL, Morgan P, Cai ZQ, Straughan RA. Prevalence of and risk factors for symptomatic dry eye disease in Singapore. Clin Exp Optom. 2015;98:45–53. doi: 10.1111/cxo.12210 25269444

[49] Shah S, Jani H. Prevalence and associated factors of dry eye: Our experience in patients above 40 years of age at a Tertiary Care Center. Oman J Ophthalmol. 2015;8: 151–6. doi: 10.4103/0974-620X.169910 26903719 PMC4738658

[50] Olaniyan SI, Fasina O, Bekibele CO, Ogundipe AO. Dry eye disease in an adult population in South-West Nigeria. Cont Lens Anterior Eye. 2016;39:359–64. doi: 10.1016/j.clae.2016.06.008 27396514

[51] Alshamrani AA, Almousa AS, Almulhim AA, Alafaleq AA, Alosaimi MB, Alqahtani AM, et al. Prevalence and Risk Factors of Dry Eye Symptoms in a Saudi Arabian Population. Middle East Afr J Ophthalmol. 2017;24:67–73. doi: 10.4103/meajo.MEAJO_281_16 28936049 PMC5598305

[52] Farrand KF, Fridman M, Stillman IÖ, Schaumberg DA. Prevalence of Diagnosed Dry Eye Disease in the United States Among Adults Aged 18 Years and Older. Am J Ophthalmol. 2017;182:90–98. doi: 10.1016/j.ajo.2017.06.033 28705660

[53] Man REK, Veerappan AR, Tan SP, Fenwick EK, Sabanayagam C, Chua J, et al. Incidence and risk factors of symptomatic dry eye disease in Asian Malays from the Singapore Malay Eye Study. Ocul Surf. 2017;15:742–748. doi: 10.1016/j.jtos.2017.04.004 28442380

[54] Gong YY, Zhang F, Zhou J, Li J, Zhang GH, Wang JL, et al. Prevalence of Dry Eye in Uyghur and Han Ethnic Groups in Western China. Ophthalmic Epidemiol. 2017;24:181–187. doi: 10.1080/09286586.2016.1263996 28276756

[55] Graue-Hernández EO, Serna-Ojeda JC, Estrada-Reyes C, Navas A, Arrieta-Camacho J, Jiménez-Corona A. Dry eye symptoms and associated risk factors among adults aged 50 or more years in Central Mexico. Salud Publica Mex. 2018;60:520–527. doi: 10.21149/9024 30550113

[56] Millán A, Viso E, Gude F, Parafita-Fernández A, Moraña N, Rodríguez-Ares MT. Incidence and Risk Factors of Dry Eye in a Spanish Adult Population: 11-Year Follow-Up From the Salnés Eye Study. Cornea. 2018;37:1527–1534. doi: 10.1097/ICO.0000000000001713 30067536

[57] Iglesias E, Sajnani R, Levitt RC, Sarantopoulos CD, Galor A. Epidemiology of Persistent Dry Eye-Like Symptoms After Cataract Surgery. Cornea. 2018;37:893–898. doi: 10.1097/ICO.0000000000001491 29504953 PMC5991988

[58] Ferrero A, Alassane S, Binquet C, Bretillon L, Acar N, Arnould L, et al. Dry eye disease in the elderly in a French population-based study (the Montrachet study: Maculopathy, Optic Nerve, nuTRition, neurovAsCular and HEarT diseases): Prevalence and associated factors. Ocul Surf. 2018;16:112–119. doi: 10.1016/j.jtos.2017.09.008 28939118

[59] Shehadeh-Mashor R, Mimouni M, Shapira Y, Sela T, Munzer G, Kaiserman I. Risk Factors for Dry Eye After Refractive Surgery. Cornea. 2019;38:1495–1499. doi: 10.1097/ICO.0000000000002152 31567630

[60] Zhang S, Hong J. Risk Factors for Dry Eye in Mainland China: A Multi-Center Cross-Sectional Hospital-Based Study. Ophthalmic Epidemiol. 2019;26:393–399. doi: 10.1080/09286586.2019.1632905 31218906

[61] Yasir ZH, Chauhan D, Khandekar R, Souru C, Varghese S. Prevalence and Determinants of Dry Eye Disease among 40 Years and Older Population of Riyadh (Except Capital), Saudi Arabia. Middle East Afr J Ophthalmol. 2019;26:27–32. doi: 10.4103/meajo.MEAJO_194_18 31114121 PMC6507383

[62] Arita R, Mizoguchi T, Kawashima M, Fukuoka S, Koh S, Shirakawa R, et al. Meibomian Gland Dysfunction and Dry Eye Are Similar but Different Based on a Population-Based Study: The Hirado-Takushima Study in Japan. Am J Ophthalmol. 2019;207:410–418. doi: 10.1016/j.ajo.2019.02.024 30851269

[63] Hyon JY, Yang HK, Han SB. Association between Dry Eye Disease and Psychological Stress among Paramedical Workers in Korea. Sci Rep. 2019;9:3783. doi: 10.1038/s41598-019-40539-0 30846779 PMC6405835

[64] Ben-Eli H, Aframian DJ, Ben-Chetrit E, Mevorach D, Kleinstern G, Paltiel O, et al. Shared Medical and Environmental Risk Factors in Dry Eye Syndrome, Sjogren’s Syndrome, and B-Cell Non-Hodgkin Lymphoma: A Case-Control Study. J Immunol Res. 2019;2019: 9060842. doi: 10.1155/2019/9060842 30805374 PMC6360537

[65] Yu D, Deng Q, Wang J, Chang X, Wang S, Yang R, et al. Air Pollutants are associated with Dry Eye Disease in Urban Ophthalmic Outpatients: a Prevalence Study in China. J Transl Med. 2019;17:46. doi: 10.1186/s12967-019-1794-6 30767763 PMC6376760

[66] Rossi GCM, Scudeller L, Bettio F, Pasinetti GM, Bianchi PE. Prevalence of dry eye in video display terminal users: a cross-sectional Caucasian study in Italy. Int Ophthalmol. 2019; 39:1315–1322. doi: 10.1007/s10792-018-0947-6 29881936

[67] Wang MTM, Vidal-Rohr M, Muntz A, Diprose WK, Ormonde SE, Wolffsohn JS, et al. Systemic risk factors of dry eye disease subtypes: A New Zealand cross-sectional study. Ocul Surf. 2020;18:374–380. doi: 10.1016/j.jtos.2020.04.003 32311433

[68] Shanti Y, Shehada R, Bakkar MM, Qaddumi J. Prevalence and associated risk factors of dry eye disease in 16 northern West bank towns in Palestine: a cross-sectional study. BMC Ophthalmol. 2020;20:26. doi: 10.1186/s12886-019-1290-z 31931756 PMC6958733

[69] Hanyuda A, Sawada N, Uchino M, Kawashima M, Yuki K, Tsubota K, et al. Physical inactivity, prolonged sedentary behaviors, and use of visual display terminals as potential risk factors for dry eye disease: JPHC-NEXT study. Ocul Surf. 2020;18:56–63. doi: 10.1016/j.jtos.2019.09.007 31563549

[70] Alkabbani S, Jeyaseelan L, Rao AP, Thakur SP, Warhekar PT. The prevalence, severity, and risk factors for dry eye disease in Dubai—a cross sectional study. BMC Ophthalmol. 2021;21:219. doi: 10.1186/s12886-021-01978-4 34001029 PMC8127306

[71] Vehof J, Snieder H, Jansonius N, Hammond CJ. Prevalence and risk factors of dry eye in 79,866 participants of the population-based Lifelines cohort study in the Netherlands. Ocul Surf. 2021;19:83–93. doi: 10.1016/j.jtos.2020.04.005 32376389

[72] Moss SE, Klein R, Klein BE. Incidence of dry eye in an older population. Arch Ophthalmol. 2004;122:369–73. doi: 10.1001/archopht.122.3.369 15006852

[73] Gilbard JP. Human tear film electrolyte concentrations in health and dry-eye disease. Int Ophthalmol Clin. 1994;34:27–36. doi: 10.1097/00004397-199403410-00005 8169071

[74] Cardona G, García C, Serés C, Vilaseca M, Gispets J. Blink rate, blink amplitude, and tear film integrity during dynamic visual display terminal tasks. Curr Eye Res. 2011;36: 190–7. doi: 10.3109/02713683.2010.544442 21275516

[75] Li XM, Hu L, Hu J, Wang W. Investigation of dry eye disease and analysis of the pathogenic factors in patients after cataract surgery. Cornea. 2007;26:S16–20. doi: 10.1097/ICO.0b013e31812f67ca 17881910

[76] Begley CG, Caffery B, Nichols KK, Chalmers R. Responses of contact lens wearers to a dry eye survey. Optom Vis Sci. 2000;77:40–6. doi: 10.1097/00006324-200001000-00012 10654857

[77] Petraevskiĭ AV, Trishkin KS. [Pathogenetic relationship between pterygium and dry eye syndrome (clinical and cytological study)]. Vestn Oftalmol. 2014;130:52–6. Russian. 24684067

[78] Bulat N, Cuşnir VV, Procopciuc V, Cușnir V, Cuşnir NV. Diagnosing the Dry Eye Syndrome in modern society and among patients with glaucoma: a prospective study. Rom J Ophthalmol. 2020;64:35–42. 32292856 PMC7141921

[79] Samoilă O, Stan C, Vişan O, Crăciun A, Dican L, Mera M. Influenţa intervenţiilor chirurgicale pe gldb deschis asupra secreţiei lacrimale [The influence of ocular surgery for lacrimal secretion]. Oftalmologia. 2007;51:81–5. Romanian. 18064960

[80] Galor A, Feuer W, Lee DJ, Florez H, Faler AL, Zann KL, et al. Depression, post-traumatic stress disorder, and dry eye syndrome: a study utilizing the national United States Veterans Affairs administrative database. Am J Ophthalmol. 2012;154:340–346.e2. doi: 10.1016/j.ajo.2012.02.009 22541654

[81] Ong ES, Alghamdi YA, Levitt RC, McClellan AL, Lewis G, Sarantopoulos CD, et al. Longitudinal Examination of Frequency of and Risk Factors for Severe Dry Eye Symptoms in US Veterans. JAMA Ophthalmol. 2017;135:116–123. doi: 10.1001/jamaophthalmol.2016.4925 28006039 PMC13531036

[82] Bielory L. Ocular toxicity of systemic asthma and allergy treatments. Curr Allergy Asthma Rep. 2006;6:299–305. doi: 10.1007/s11882-006-0063-y 16822382

[83] Tang YL, Cheng YL, Ren YP, Yu XN, Shentu XC. Metabolic syndrome risk factors and dry eye syndrome: a Meta-analysis. Int J Ophthalmol. 2016;9:1038–45. doi: 10.18240/ijo.2016.07.17 27500114 PMC4951660

[84] Xu L, You QS, Jonas JB. Prevalence of alcohol consumption and risk of ocular diseases in a general population: the Beijing Eye Study. Ophthalmology. 2009;116:1872–9. doi: 10.1016/j.ophtha.2009.04.014 19712977

[85] Woo YR, Cho M, Ju HJ, Bae JM, Cho SH, Lee JD, et al. Ocular Comorbidities in Rosacea: A Case-Control Study Based on Seven Institutions. J Clin Med. 2021;10:2897. doi: 10.3390/jcm10132897 34209731 PMC8267744

[86] Eckstein AK, Finkenrath A, Heiligenhaus A, Renzing-Köhler K, Esser J, Krüger C, et al. Dry eye syndrome in thyroid-associated ophthalmopathy: lacrimal expression of TSH receptor suggests involvement of TSHR-specific autoantibodies. Acta Ophthalmol Scand. 2004;82:291–7. doi: 10.1111/j.1395-3907.2004.00268.x 15115450

[87] Sharon Y, Schlesinger N. Beyond Joints: a Review of Ocular Abnormalities in Gout and Hyperuricemia. Curr Rheumatol Rep. 2016;18:37. doi: 10.1007/s11926-016-0586-8 27138165

[88] Koktekir BE, Celik G, Karalezli A, Kal A. Dry eyes and migraines: is there really a correlation? Cornea. 2012;31:1414–6. doi: 10.1097/ICO.0b013e318247ec2a 22710496

[89] Jeng YT, Lin SY, Hu HY, Lee OK, Kuo LL. Osteoporosis and dry eye syndrome: A previously unappreciated association that may alert active prevention of fall. PLoS One. 2018; 13:e0207008. doi: 10.1371/journal.pone.0207008 30395639 PMC6218084

[90] Goulden V, Layton AM, Cunliffe WJ. Long-term safety of isotretinoin as a treatment for acne vulgaris. Br J Dermatol. 1994;131:360–3. doi: 10.1111/j.1365-2133.1994.tb08524.x 7918010

[91] Klocek P, Klocek M, Jeśman C. [Assessment of the incidence of dry eye syndrome symptoms in Military Police soldiers serving outside the country]. Pol Merkur Lekarski. 2020; 48:82–86. 32352936

[92] Youn JS, Seo JW, Park W, Park S, Jeon KJ. Prediction Model for Dry Eye Syndrome Incidence Rate Using Air Pollutants and Meteorological Factors in South Korea: Analysis of Sub-Region Deviations. Int J Environ Res Public Health. 2020;17:4969. doi: 10.3390/ijerph17144969 32664192 PMC7399894

