# Supplementary material for: Identified risk factors for dry eye syndrome: A systematic review and meta-analysis
Source: PLoS One. 2022 Aug 19;17(8):e0271267. doi: 10.1371/journal.pone.0271267 (PMC9390932; doi:10.1371/journal.pone.0271267)
Supplement: S1 Table — (DOCX) [file pone.0271267.s002.docx]

Table S1. Quality scores of prospective cohort studies using Newcastle-Ottawa Scale.

| Study | Selection | | | | Comparability | Outcome | | | NOS |
| --- | --- | --- | --- | --- | --- | --- | --- | --- | --- |
|  | Representativeness of the exposed cohort | Selection of the non exposed cohort | Ascertainment  of exposure | Demonstration that outcomes was not present at start of study | Comparability on the basis of the design or analysis | Assessment of outcome | Adequate follow-up duration | Adequate follow-up rate | Overall score |
| BDES 2000 [25] | 1 | 1 | 1 | 1 | 2 | 1 | 1 | 0 | 8 |
| Lee 2002 [26] | 1 | 1 | 1 | 1 | 1 | 1 | 1 | 0 | 7 |
| BMES 2003 [27] | 1 | 1 | 1 | 1 | 2 | 1 | 1 | 0 | 8 |
| Sahai 2005 [28] | 0 | 1 | 1 | 1 | 1 | 1 | 1 | 0 | 6 |
| Nichols 2006 [29] | 0 | 1 | 1 | 1 | 1 | 1 | 1 | 0 | 6 |
| Uchino 2008 [30] | 1 | 1 | 1 | 1 | 1 | 1 | 1 | 0 | 7 |
| Lu 2008 [31] | 0 | 1 | 1 | 1 | 1 | 1 | 1 | 0 | 6 |
| PHS 2009 [32] | 1 | 1 | 1 | 1 | 2 | 1 | 1 | 0 | 8 |
| TSES 2009 [33] | 1 | 1 | 1 | 1 | 1 | 1 | 1 | 0 | 7 |
| BES 2009 [34] | 1 | 1 | 1 | 1 | 1 | 1 | 1 | 0 | 7 |
| THES 2010 [35] | 1 | 1 | 1 | 1 | 1 | 1 | 1 | 0 | 7 |
| Kim 2011 [36] | 0 | 1 | 1 | 1 | 1 | 1 | 1 | 0 | 6 |
| Koumi Study 2011 [37] | 1 | 1 | 1 | 1 | 2 | 1 | 1 | 0 | 8 |
| USVAP 2011 [38] | 1 | 1 | 1 | 1 | 1 | 1 | 1 | 0 | 7 |
| Zhang 2012 [39] | 0 | 1 | 1 | 1 | 1 | 1 | 1 | 0 | 6 |
| TNHRI 2012 [40] | 1 | 1 | 1 | 1 | 1 | 1 | 1 | 0 | 7 |
| TOS 2013 [41] | 1 | 1 | 1 | 1 | 2 | 1 | 1 | 0 | 8 |
| TwinUK 2014 [42] | 1 | 1 | 1 | 1 | 2 | 1 | 1 | 0 | 8 |
| KNHNES 2014 [43] | 1 | 1 | 1 | 1 | 1 | 1 | 1 | 0 | 7 |
| Moon 2014 [44] | 0 | 1 | 1 | 1 | 1 | 1 | 1 | 0 | 6 |
| BDOS 2014 [45] | 1 | 1 | 1 | 1 | 2 | 1 | 1 | 0 | 8 |
| TNHI 2015 [46] | 1 | 1 | 1 | 1 | 1 | 1 | 1 | 0 | 7 |
| Yang 2015 [47] | 0 | 1 | 1 | 1 | 1 | 1 | 1 | 0 | 6 |
| Tan 2015 [48] | 0 | 1 | 1 | 1 | 1 | 1 | 1 | 0 | 6 |
| Shah 2015 [49] | 0 | 1 | 1 | 1 | 1 | 1 | 1 | 0 | 6 |
| Olaniyan 2016 [50] | 0 | 1 | 1 | 1 | 1 | 1 | 1 | 0 | 6 |
| Alshamrani 2017 [51] | 1 | 1 | 1 | 1 | 1 | 1 | 1 | 0 | 7 |
| NHWS 2017 [52] | 1 | 1 | 1 | 1 | 1 | 1 | 1 | 0 | 7 |
| SMES 2017 [53] | 1 | 1 | 1 | 1 | 1 | 1 | 1 | 0 | 7 |
| Gong 2017 [54] | 1 | 1 | 1 | 1 | 1 | 1 | 1 | 0 | 7 |
| Asiedu 2017 [55] | 0 | 1 | 1 | 1 | 1 | 1 | 1 | 0 | 6 |
| Graue-Hernandez 2018 [56] | 1 | 1 | 1 | 1 | 1 | 1 | 1 | 0 | 7 |
| SES 2018 [57] | 0 | 1 | 1 | 1 | 1 | 1 | 1 | 0 | 6 |
| Iglesias 2018 [58] | 0 | 1 | 1 | 1 | 1 | 1 | 1 | 0 | 6 |
| TMS 2018 [59] | 1 | 1 | 1 | 1 | 2 | 1 | 1 | 0 | 8 |
| Shehadeh-Mashor 2019 [60] | 1 | 1 | 1 | 1 | 1 | 1 | 1 | 0 | 7 |
| Zhang 2019 [61] | 1 | 1 | 1 | 1 | 2 | 1 | 1 | 0 | 8 |
| Yasir 2019 [62] | 0 | 1 | 1 | 1 | 1 | 1 | 1 | 0 | 6 |
| HTS 2019 [63] | 1 | 1 | 1 | 1 | 1 | 1 | 1 | 0 | 7 |
| Hyon 2019 [64] | 0 | 1 | 1 | 1 | 1 | 1 | 1 | 0 | 6 |
| Ben-Eli 2019 [65] | 0 | 1 | 1 | 1 | 1 | 1 | 1 | 0 | 6 |
| Yu 2019 [66] | 1 | 1 | 1 | 1 | 1 | 1 | 1 | 0 | 7 |
| Rossi 2019 [67] | 0 | 1 | 1 | 1 | 1 | 1 | 1 | 0 | 6 |
| Wang 2020 [68] | 1 | 1 | 1 | 1 | 1 | 1 | 1 | 0 | 7 |
| Shanti 2020 [69] | 0 | 1 | 1 | 1 | 1 | 1 | 1 | 0 | 6 |
| JPHC 2020 [70] | 1 | 1 | 1 | 1 | 2 | 1 | 1 | 0 | 8 |
| Alkabbani 2021 [71] | 0 | 1 | 1 | 1 | 1 | 1 | 1 | 0 | 6 |
| LCS 2021 [72] | 1 | 1 | 1 | 1 | 2 | 1 | 1 | 0 | 8 |
