# Supplementary material for: Identified risk factors for dry eye syndrome: A systematic review and meta-analysis
Source: PLoS One. 2022 Aug 19;17(8):e0271267. doi: 10.1371/journal.pone.0271267 (PMC9390932; doi:10.1371/journal.pone.0271267)
Supplement: S1 File — (DOCX) [file pone.0271267.s003.docx]

Search strategy in PubMed:

1. Dry eye/MeSH
2. Dry eye/ti,ab
3. Xerophthalmia/MeSH
4. Xerophthalmia/ti,ab
5. Keratoconjunctivitis Sicca/ti,ab
6. OR/1-5
7. Risk Factors/MeSH
8. Risk Factors/ti,ab
9. OR/7-8
10. AND/6, 9
