# Supplementary material for: Identified risk factors for dry eye syndrome: A systematic review and meta-analysis
Source: PLoS One. 2022 Aug 19;17(8):e0271267. doi: 10.1371/journal.pone.0271267 (PMC9390932; doi:10.1371/journal.pone.0271267)
Supplement: S2 File — (DOCX) [file pone.0271267.s004.docx]

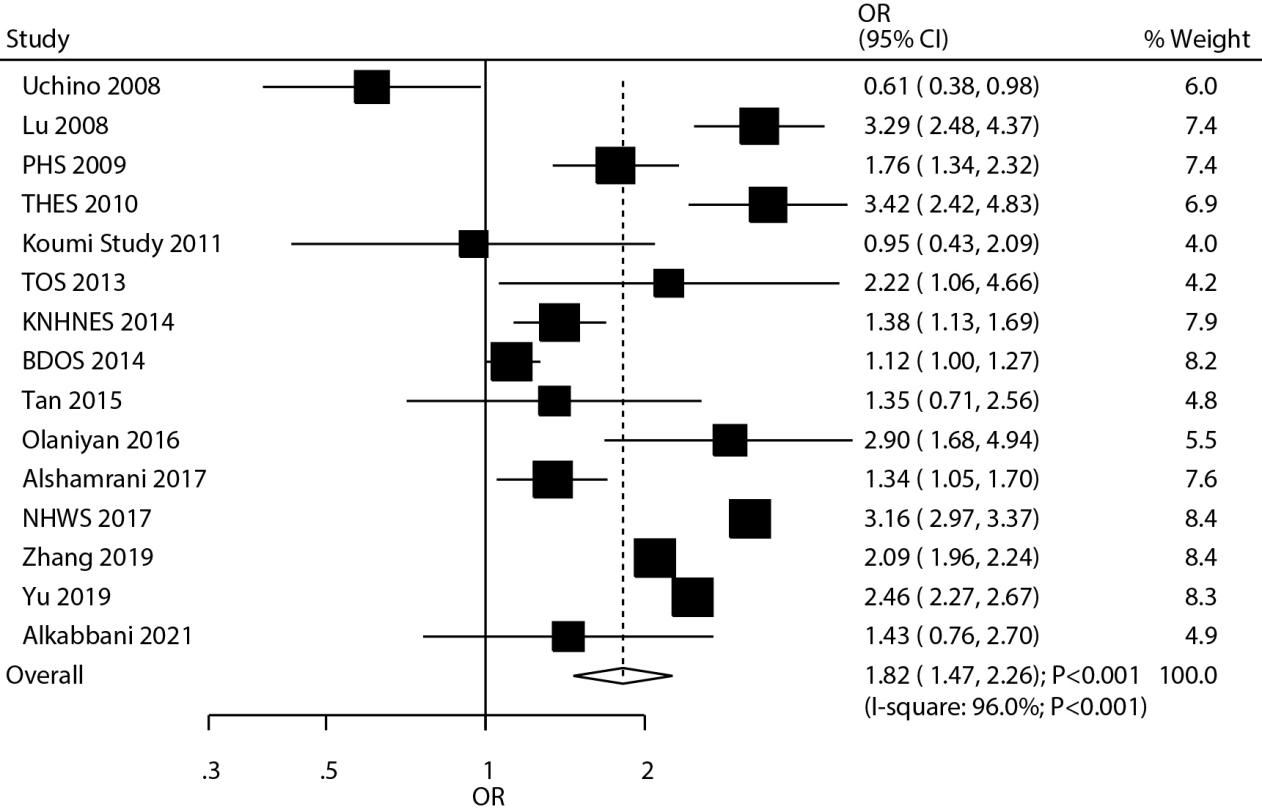


Figure S1. Elderly versus younger on subsequent dry eye syndrome risk


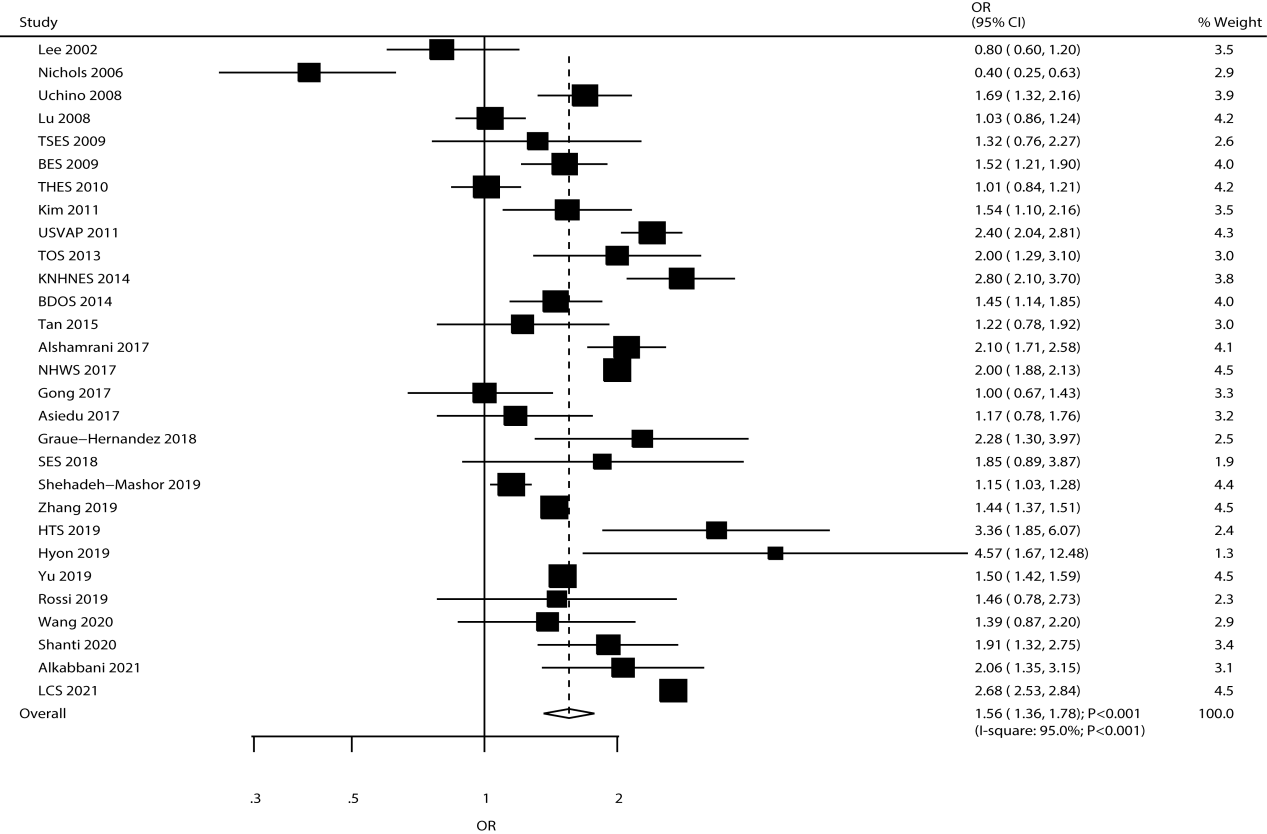


Figure S2. Female versus male on subsequent dry eye syndrome risk


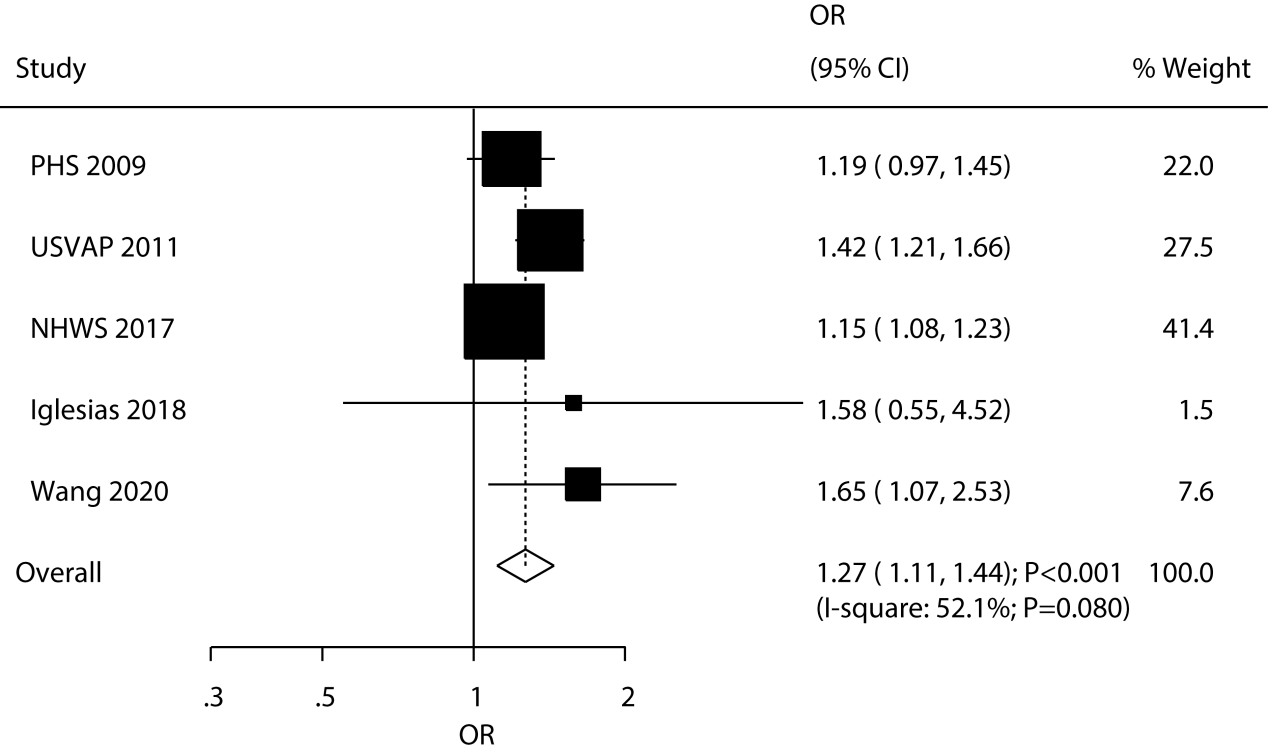


Figure S3. Other race versus white on subsequent dry eye syndrome risk


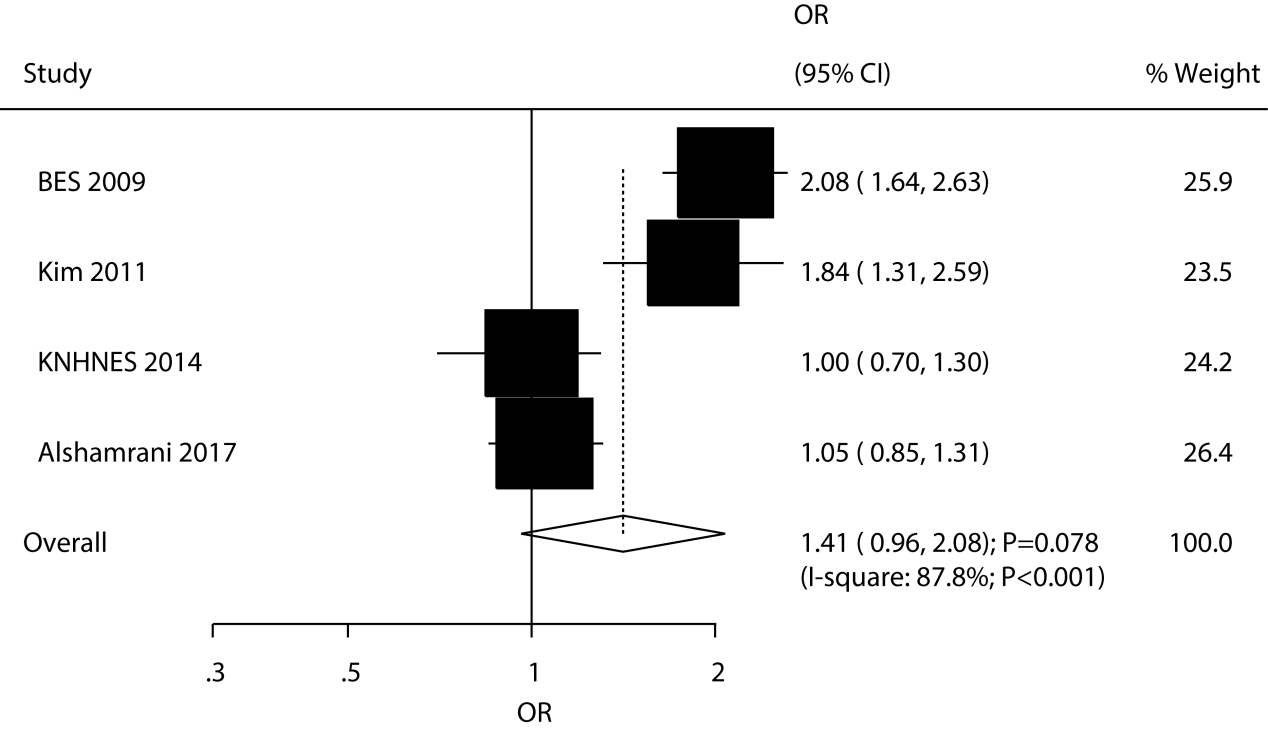


Figure S4. Urban versus rural residence on subsequent dry eye syndrome risk


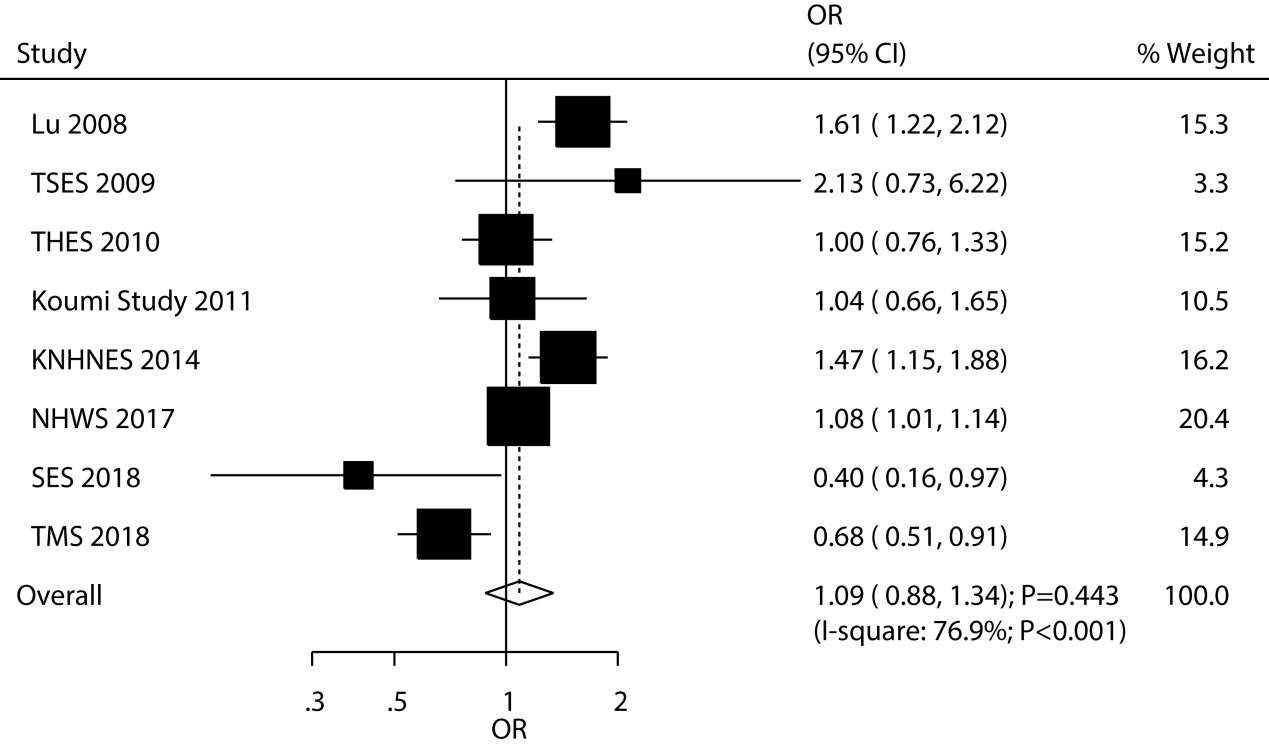


Figure S5. High versus low education level on subsequent dry eye syndrome risk


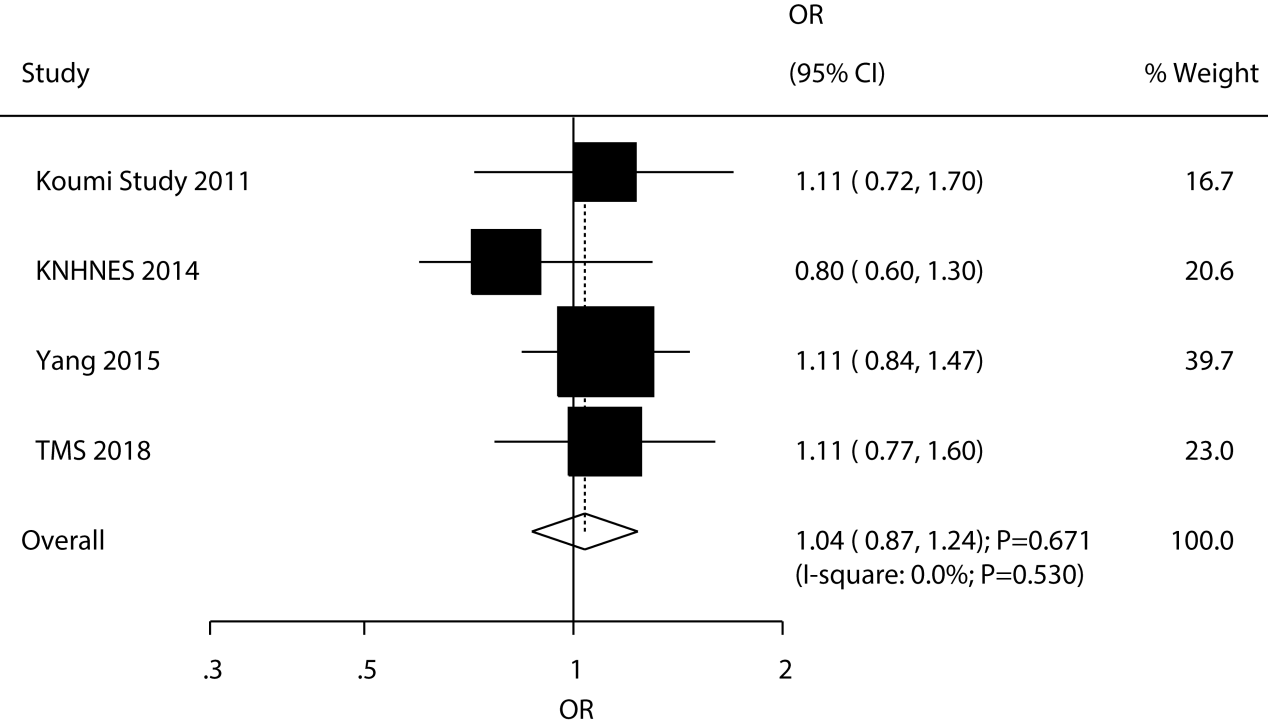


Figure S6. Association of obesity with the risk of dry eye syndrome


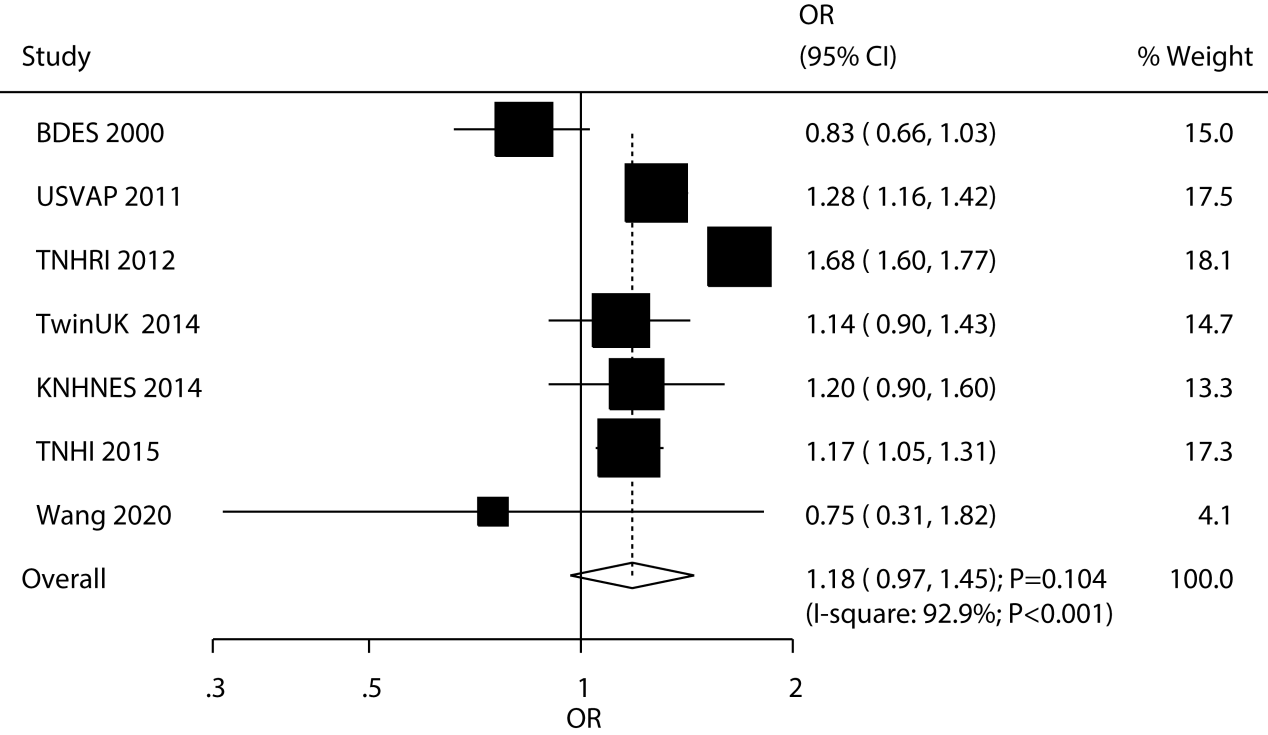


Figure S7. Association of dyslipidemia with the risk of dry eye syndrome


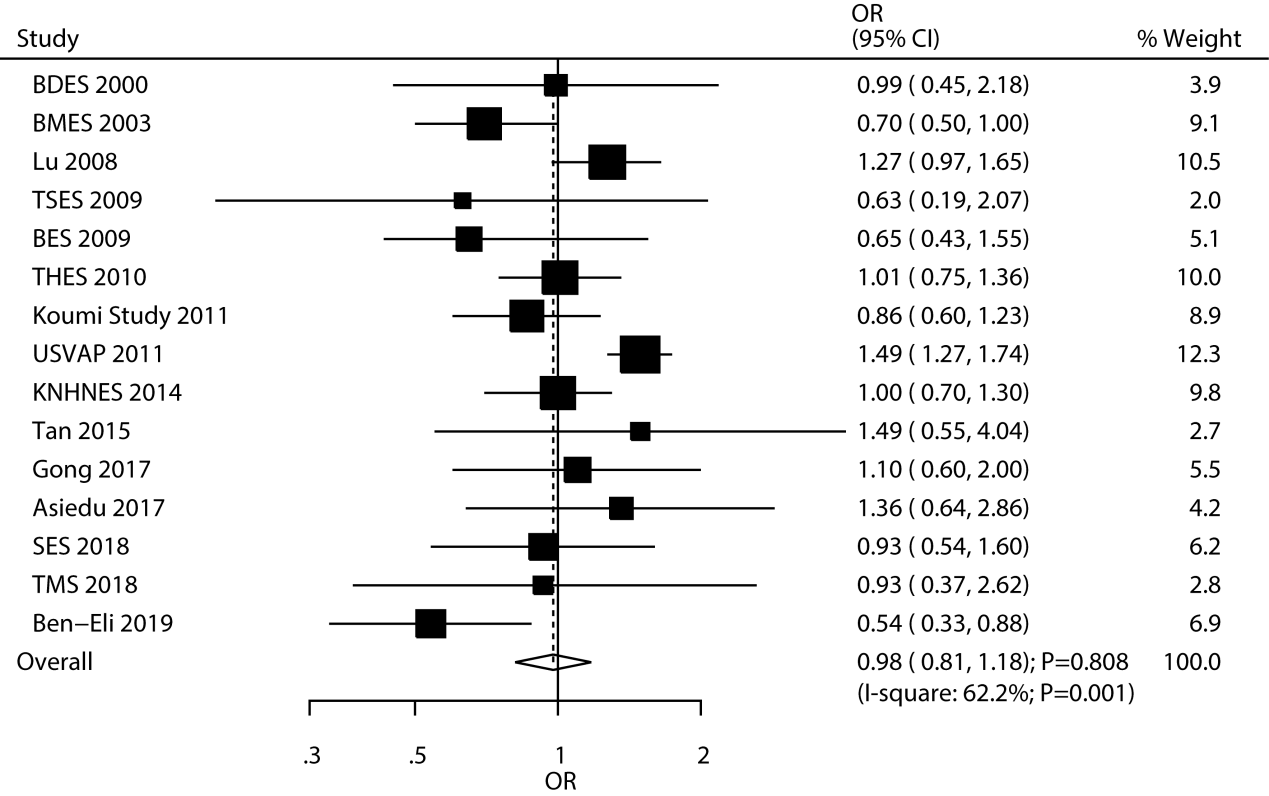


Figure S8. Alcohol intake on subsequent risk of dry eye syndrome


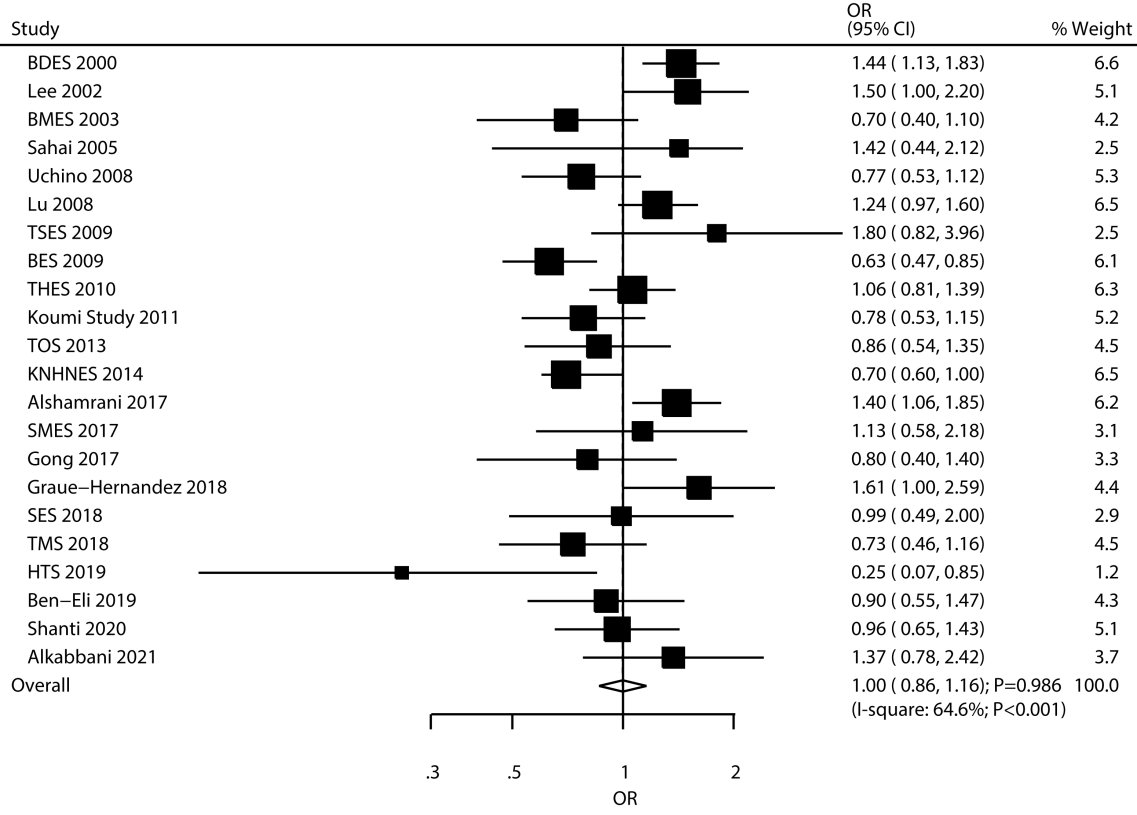


Figure S9. Association of smoking with the risk of dry eye syndrome


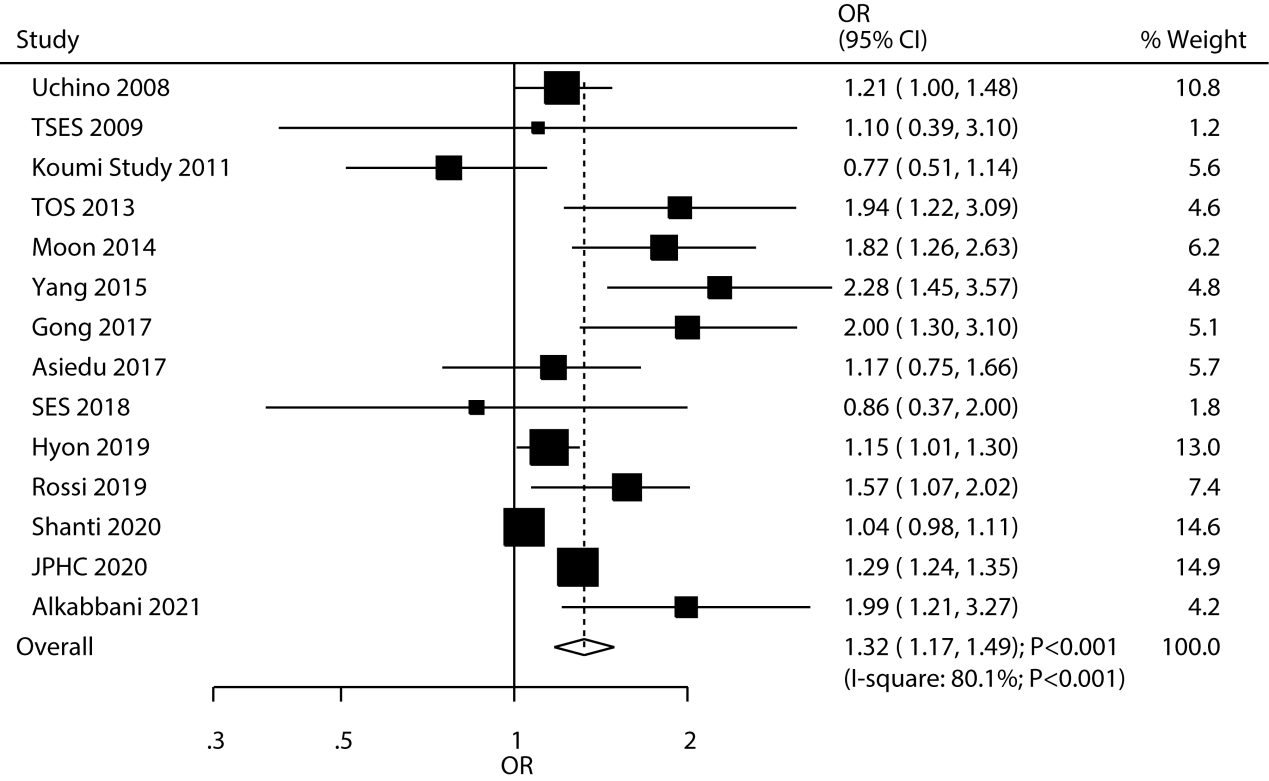


Figure S10. Association of VDT use with the risk of dry eye syndrome


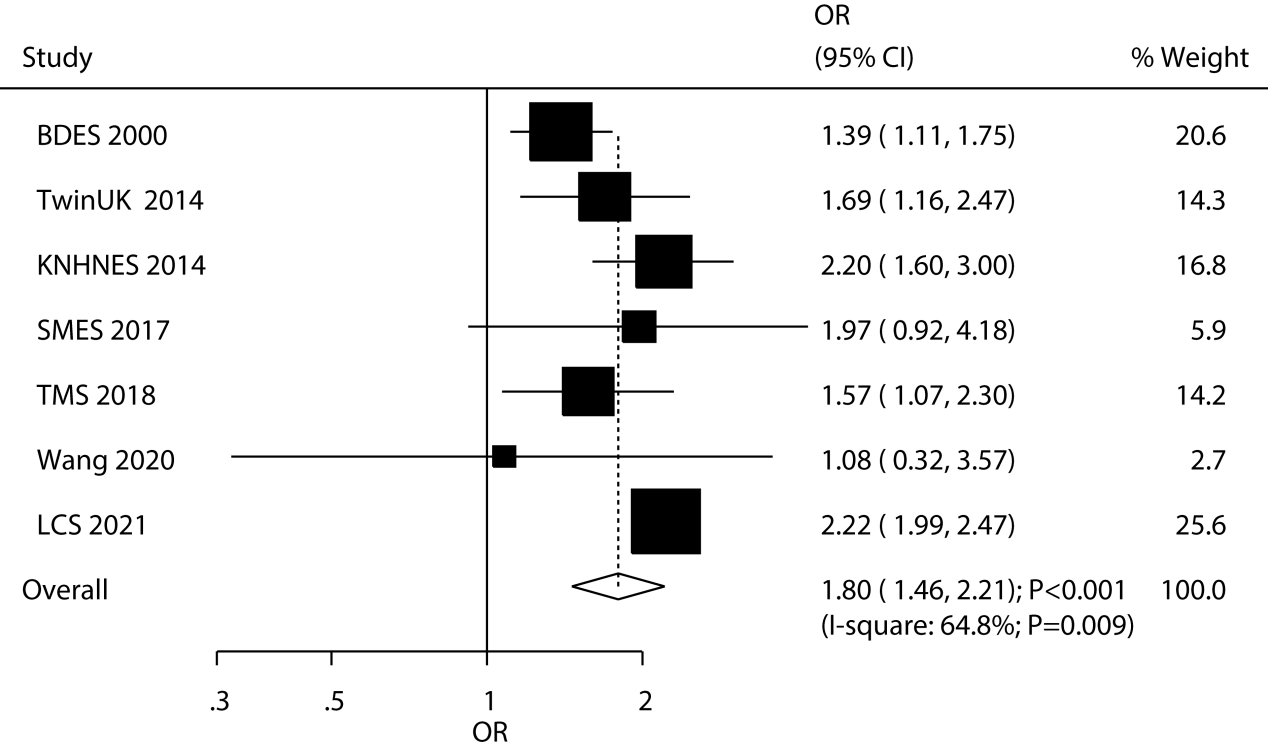


Figure S11. Association of cataract surgery with the risk of dry eye syndrome


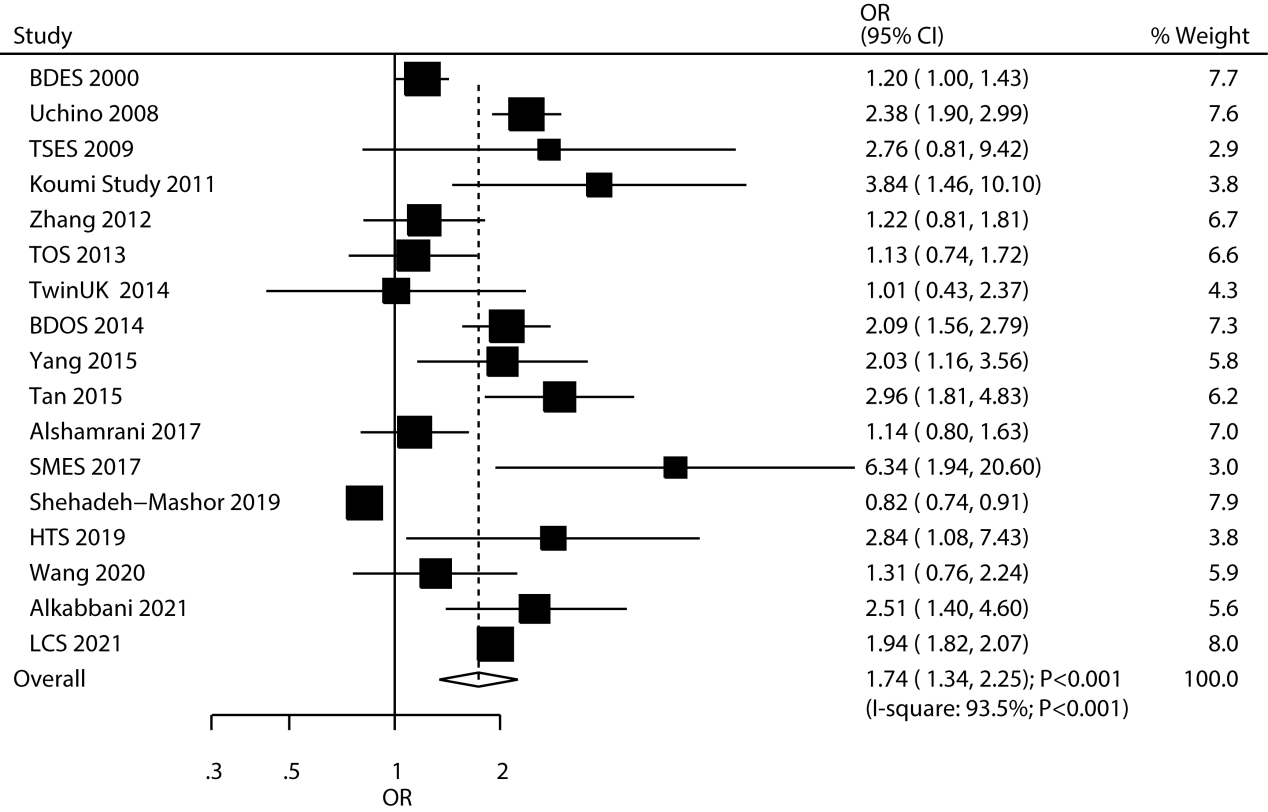


Figure S12. Association of contact lens wear with the risk of dry eye syndrome


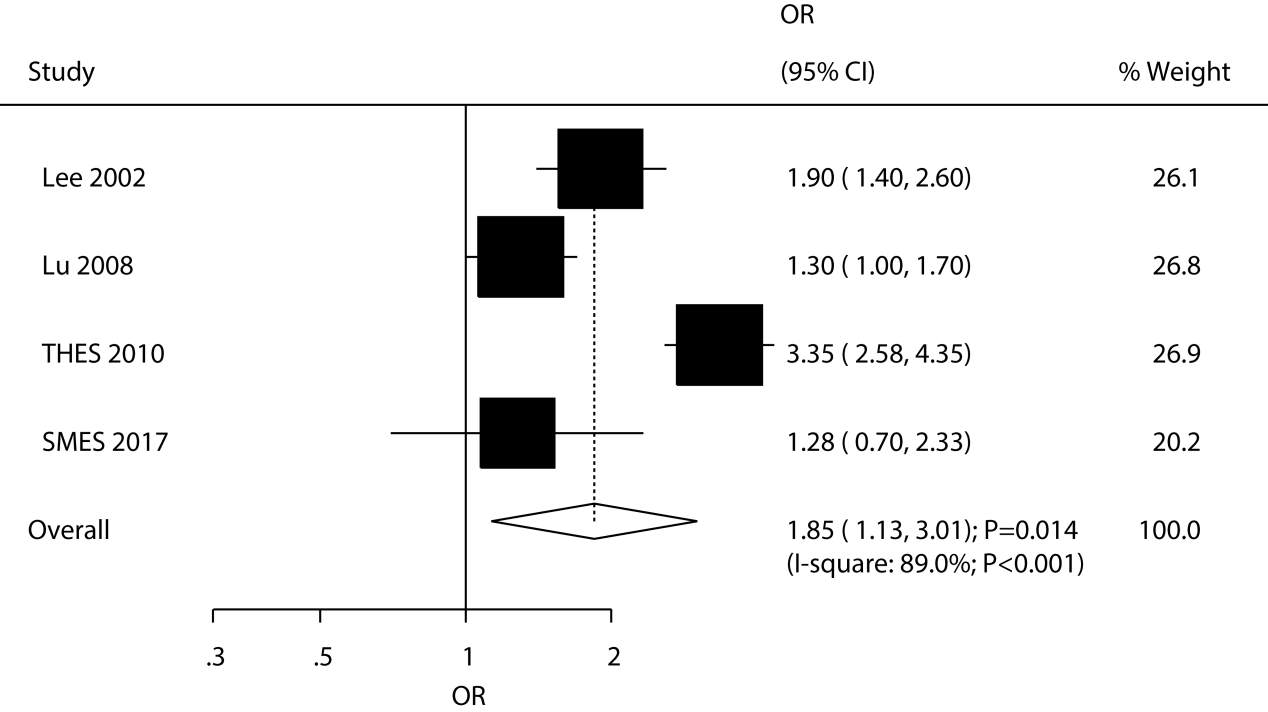


Figure S13. Association of pterygium with the risk of dry eye syndrome


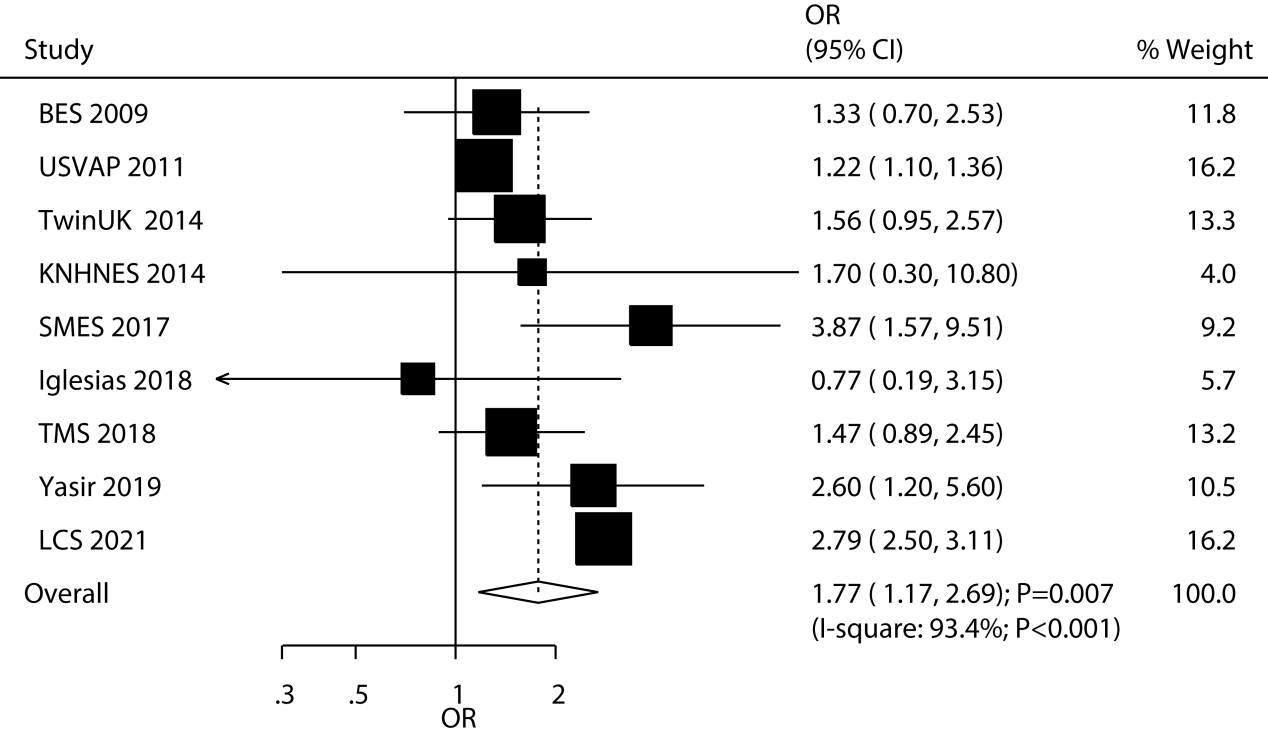


Figure S14. Association of glaucoma with the risk of dry eye syndrome


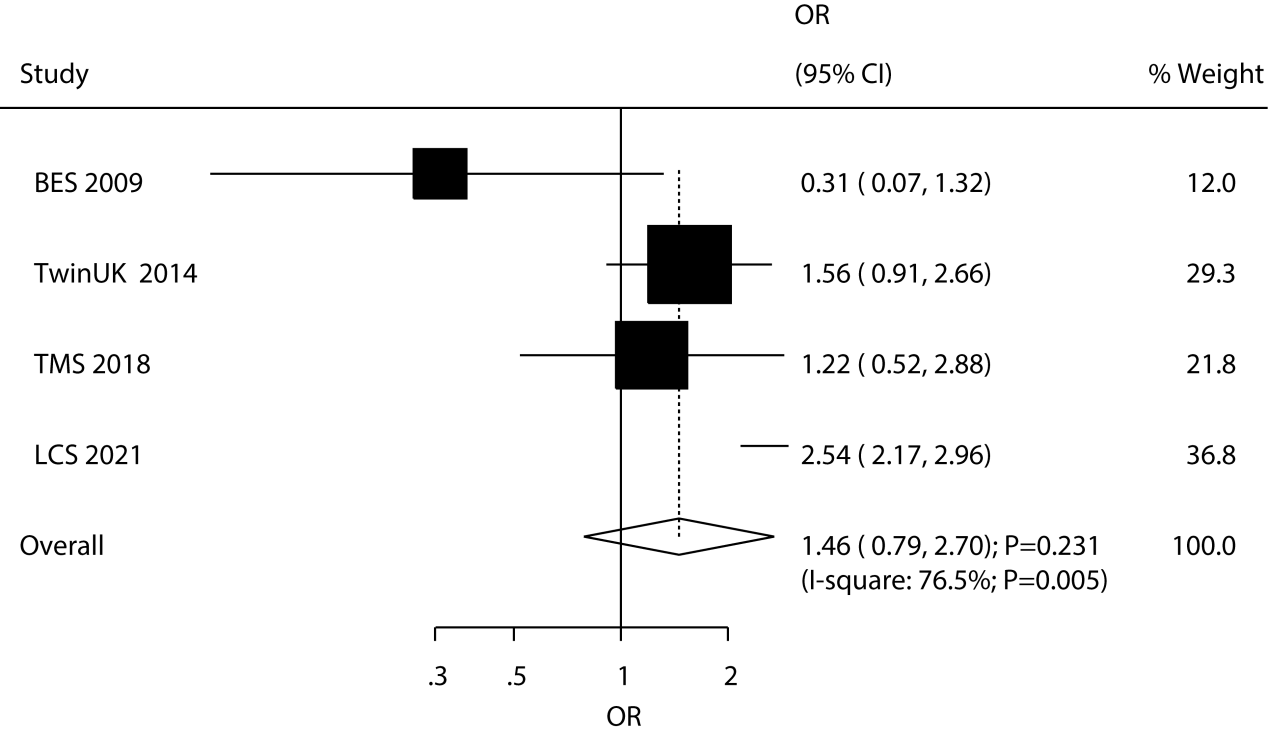


Figure S15. Association of age-related maculopathy with the risk of dry eye syndrome


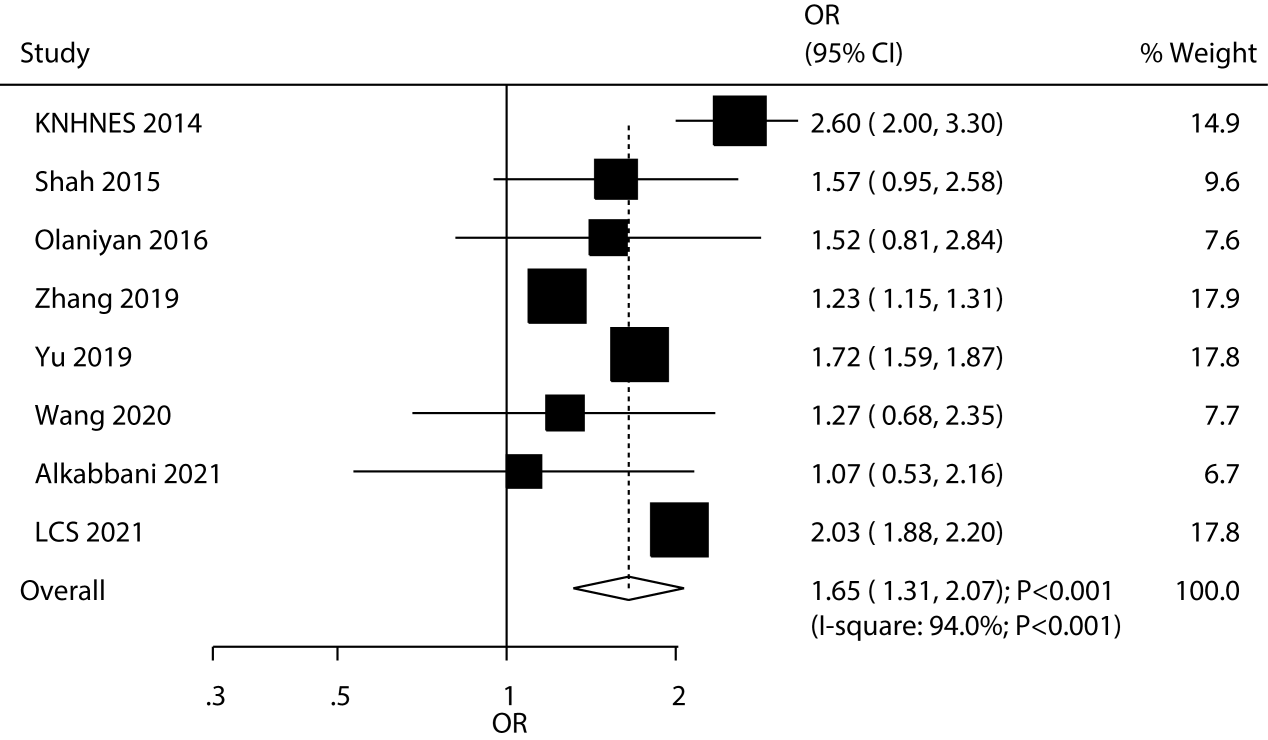


Figure S16. Association of eye surgery with the risk of dry eye syndrome


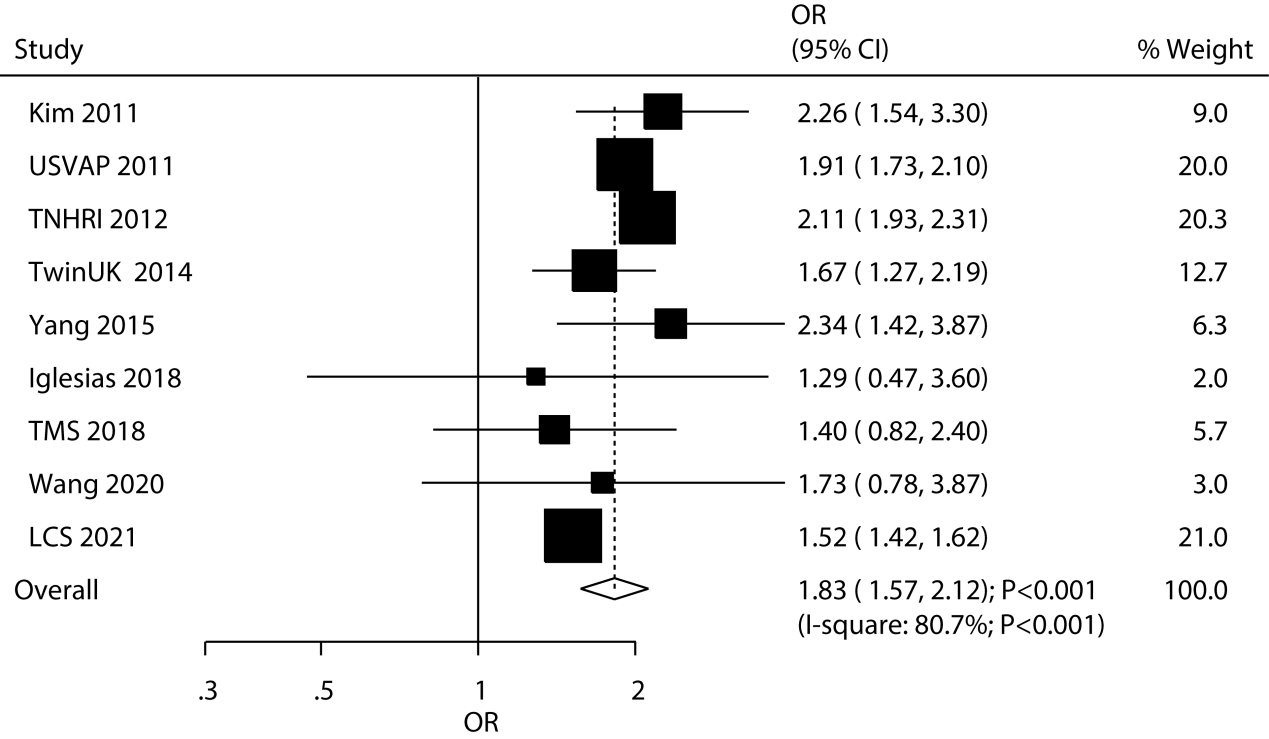


Figure S17. Association of depression with the risk of dry eye syndrome


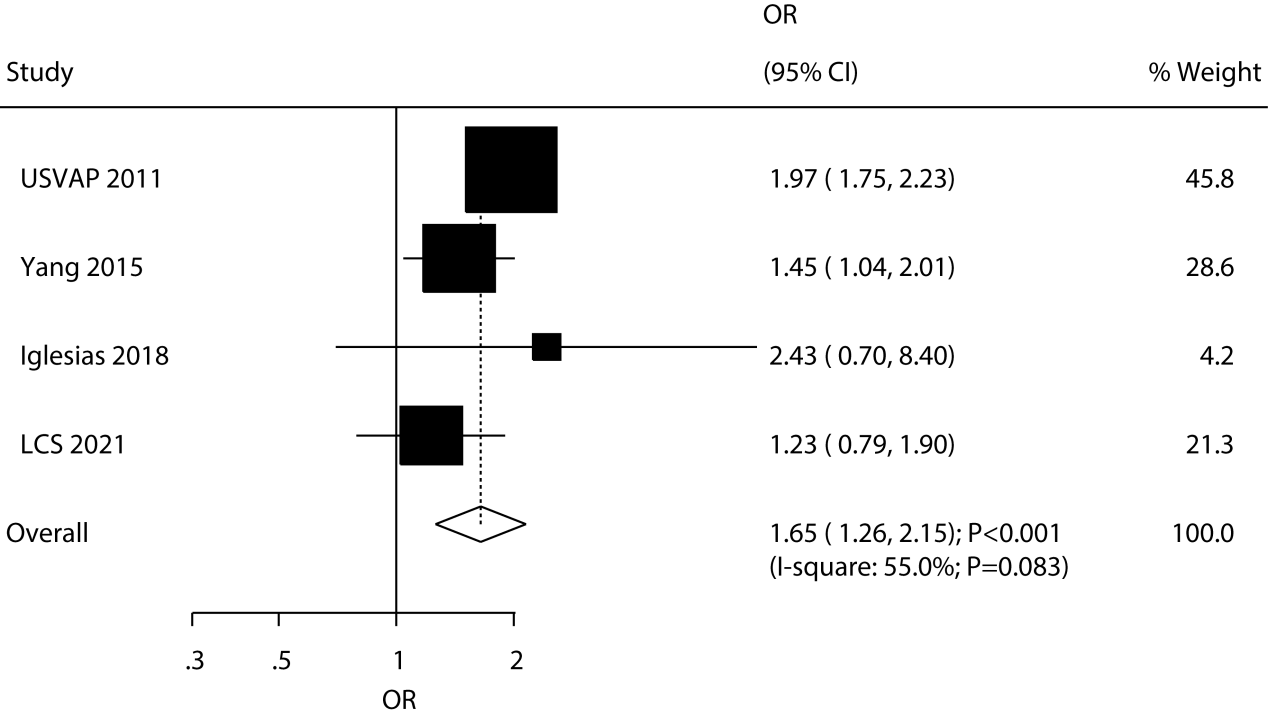


Figure S18. Association of PTSD with the risk of dry eye syndrome


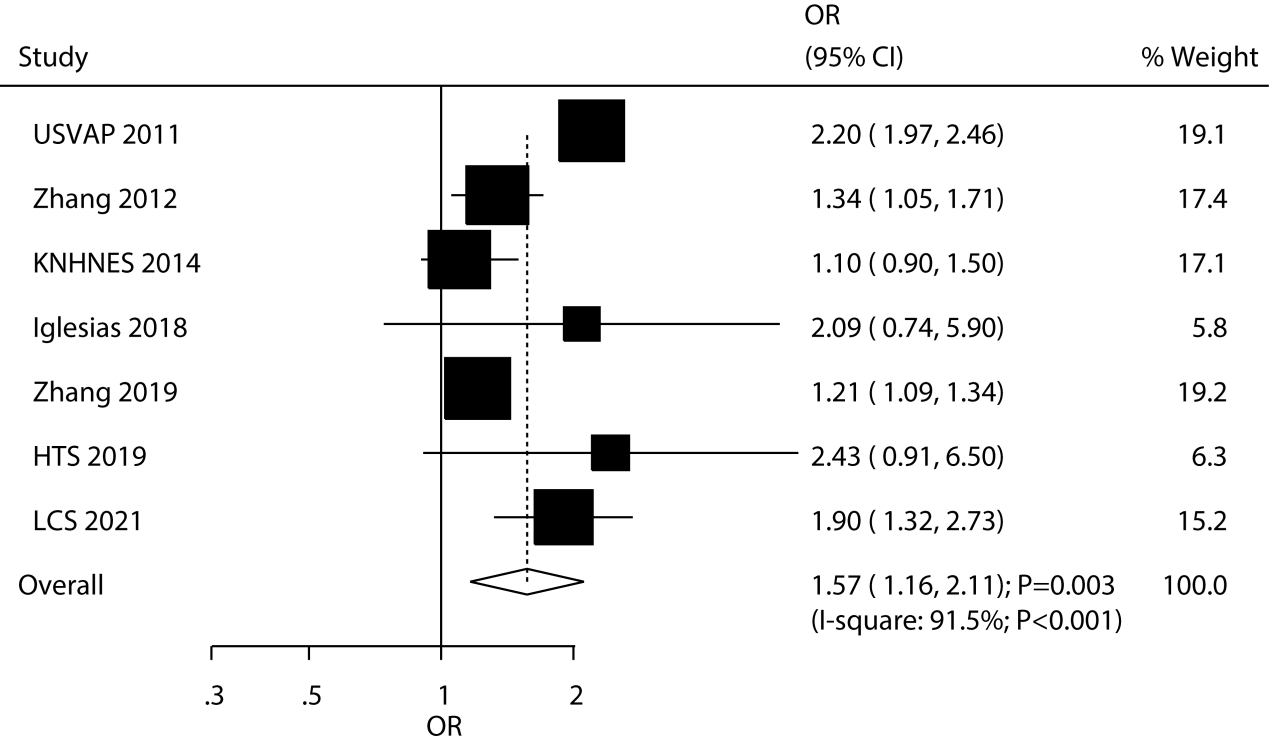


Figure S19. Association of sleep apnea with the risk of dry eye syndrome


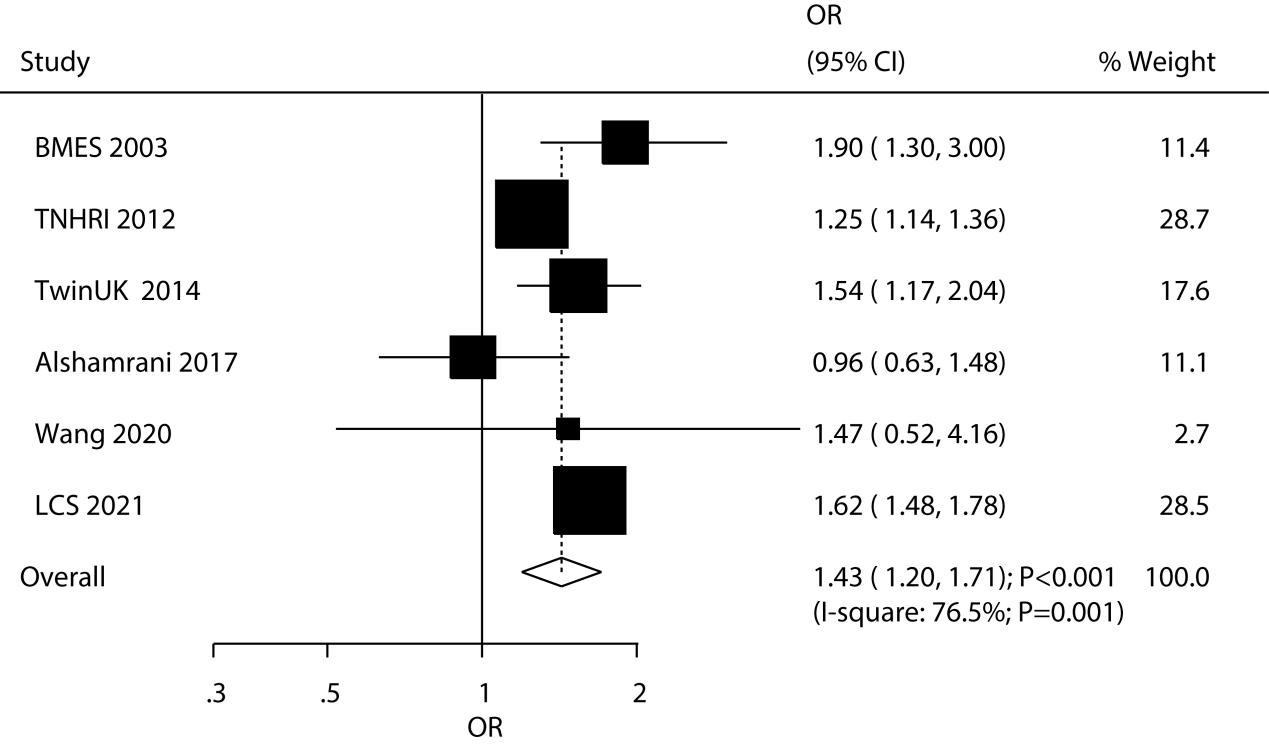


Figure S20. Association of asthma with the risk of dry eye syndrome


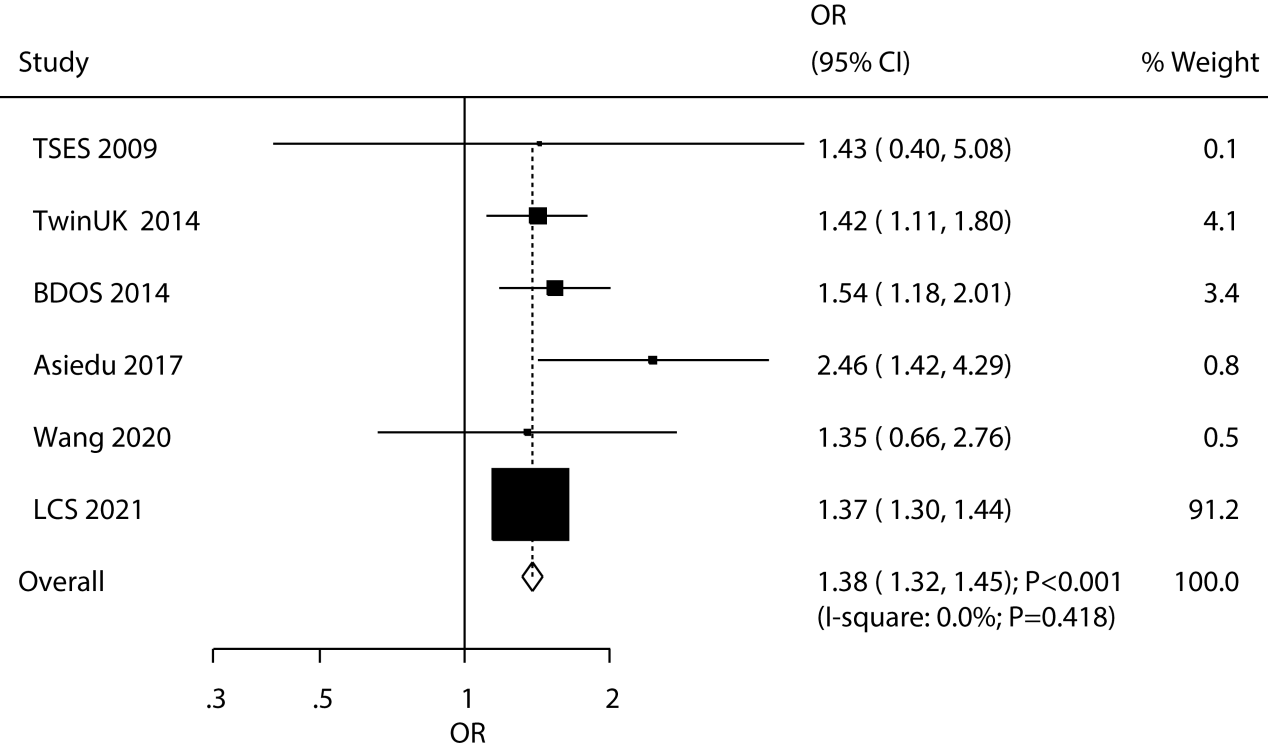


Figure S21. Association of allergy with the risk of dry eye syndrome


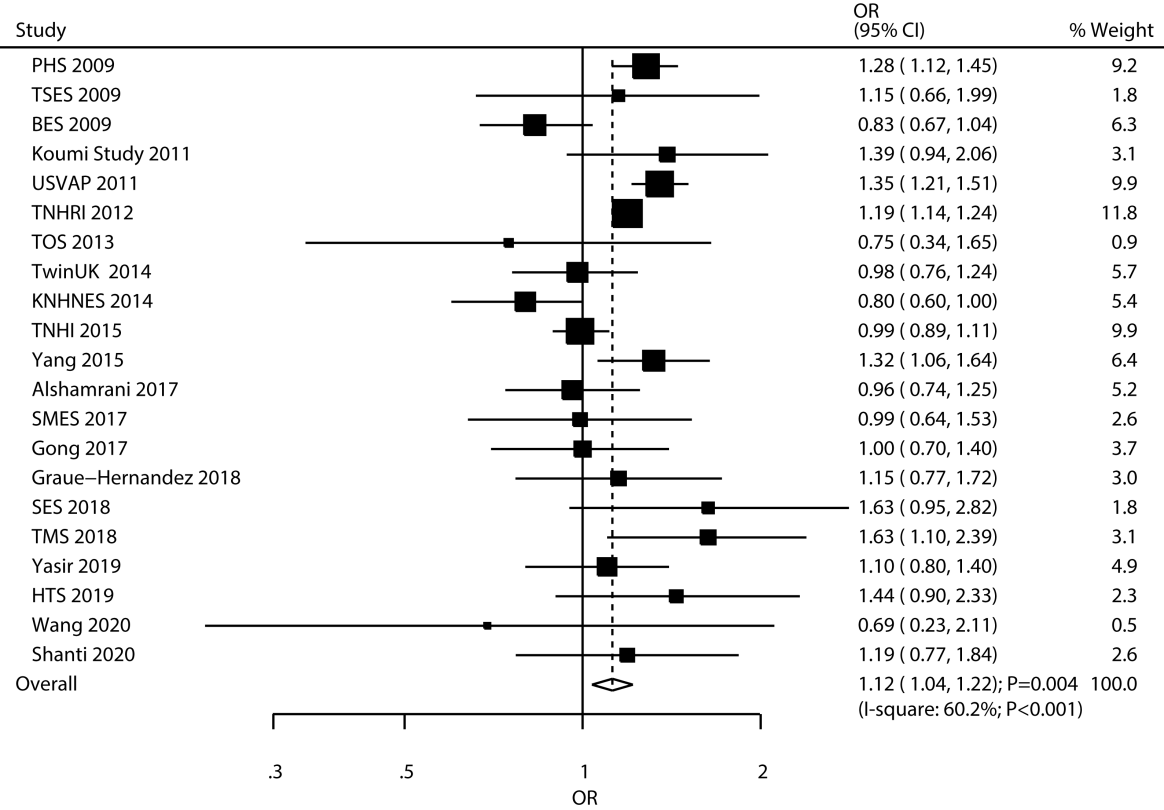


Figure S22. Association of hypertension with the risk of dry eye syndrome


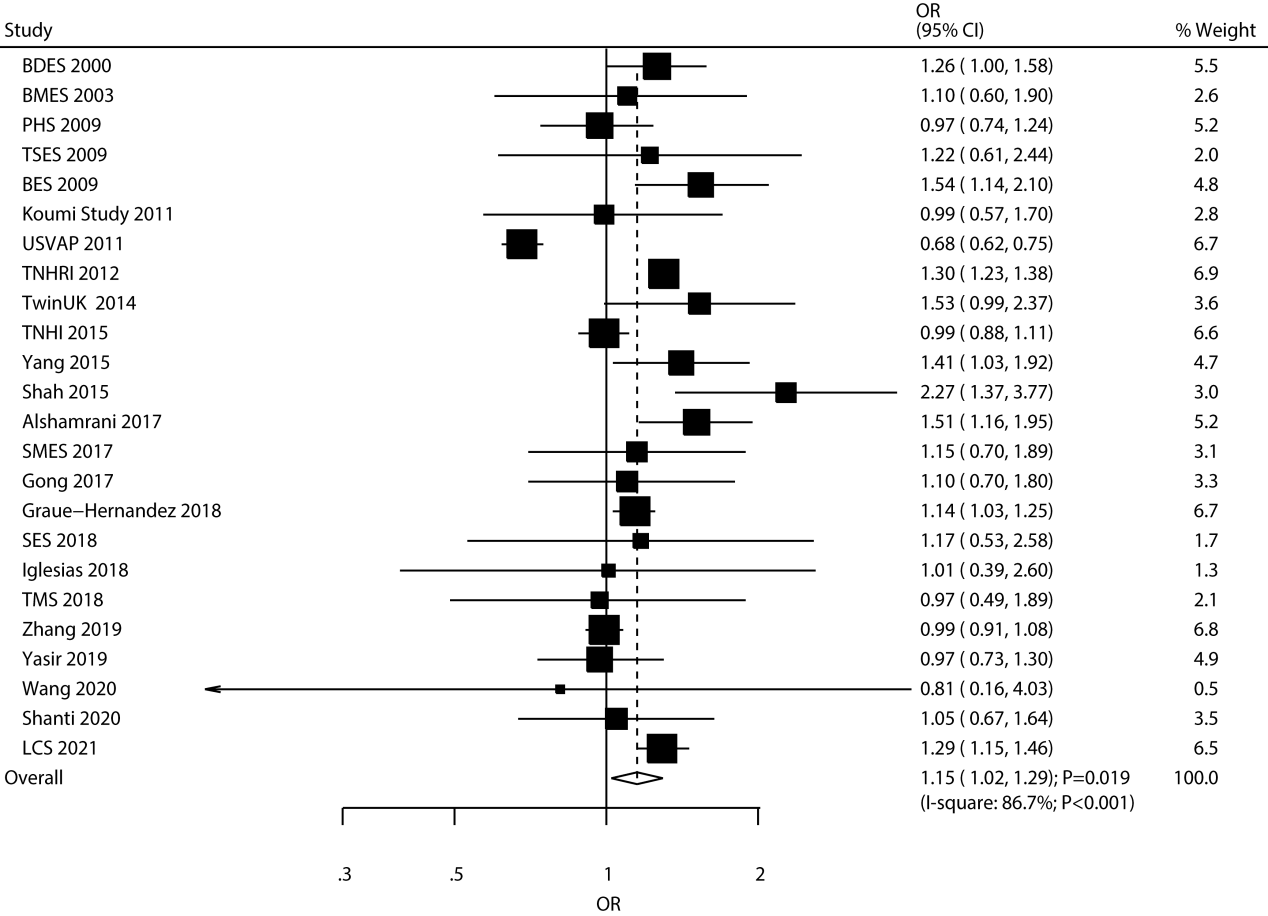


Figure S23. Association of DM with the risk of dry eye syndrome


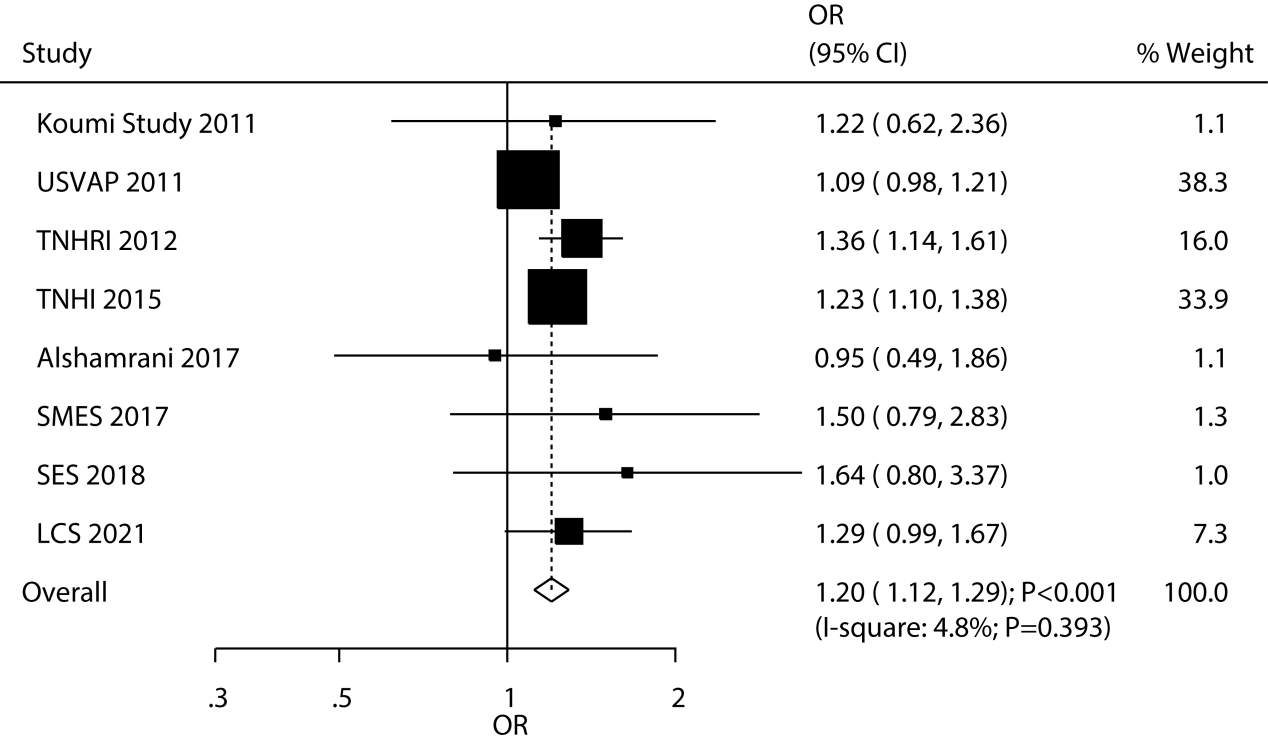


Figure S24. Association of CVD with the risk of dry eye syndrome


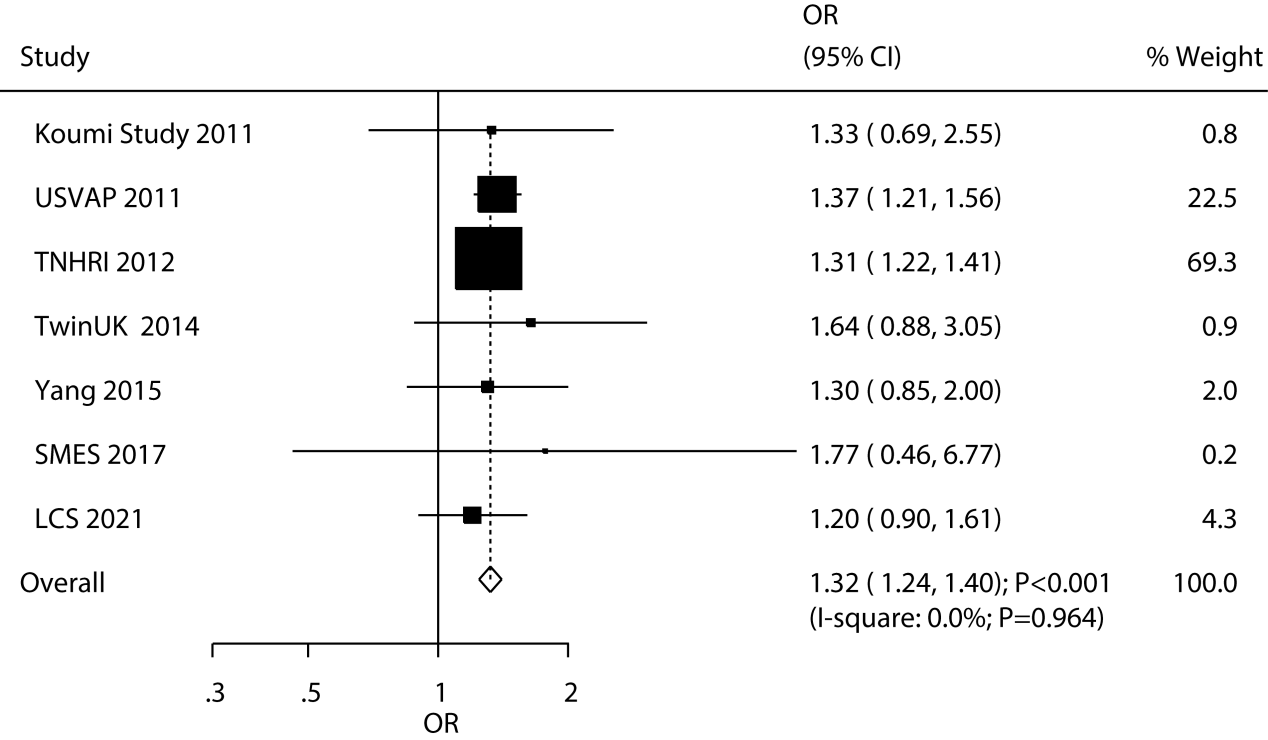


Figure S25. Association of stroke with the risk of dry eye syndrome


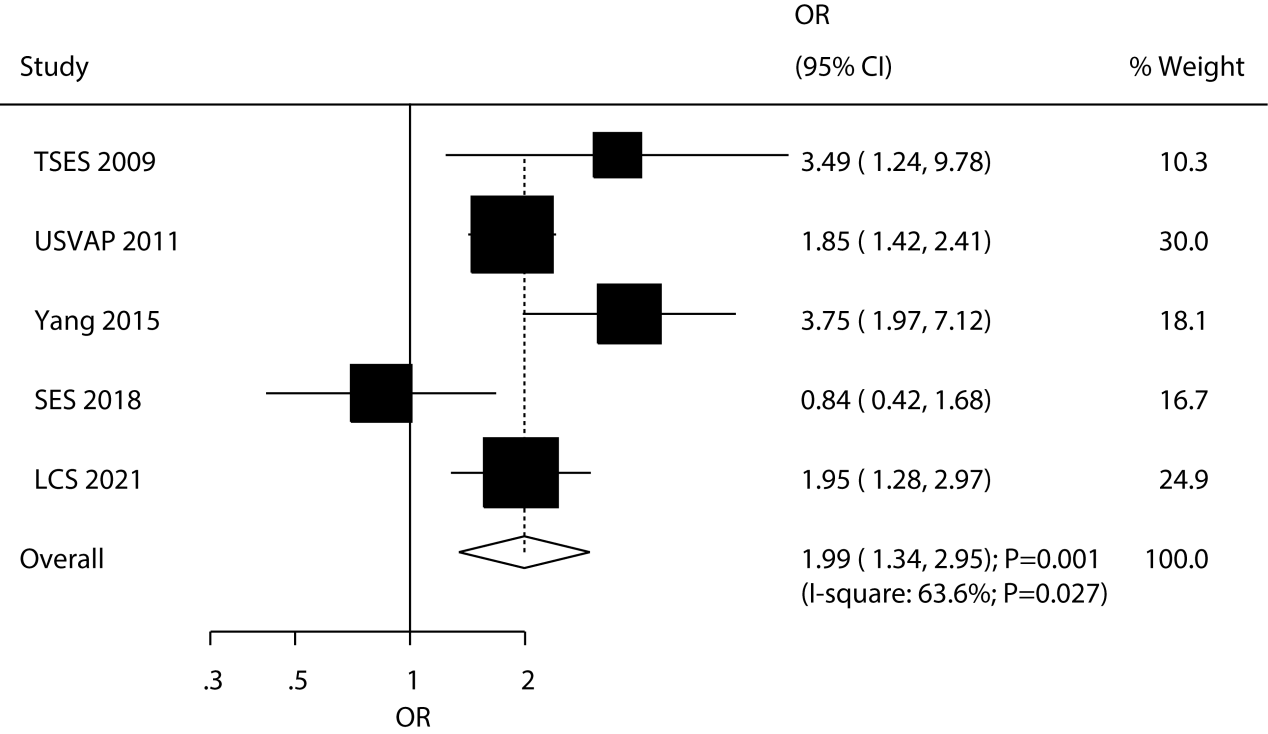


Figure S26. Association of rosacea with the risk of dry eye syndrome


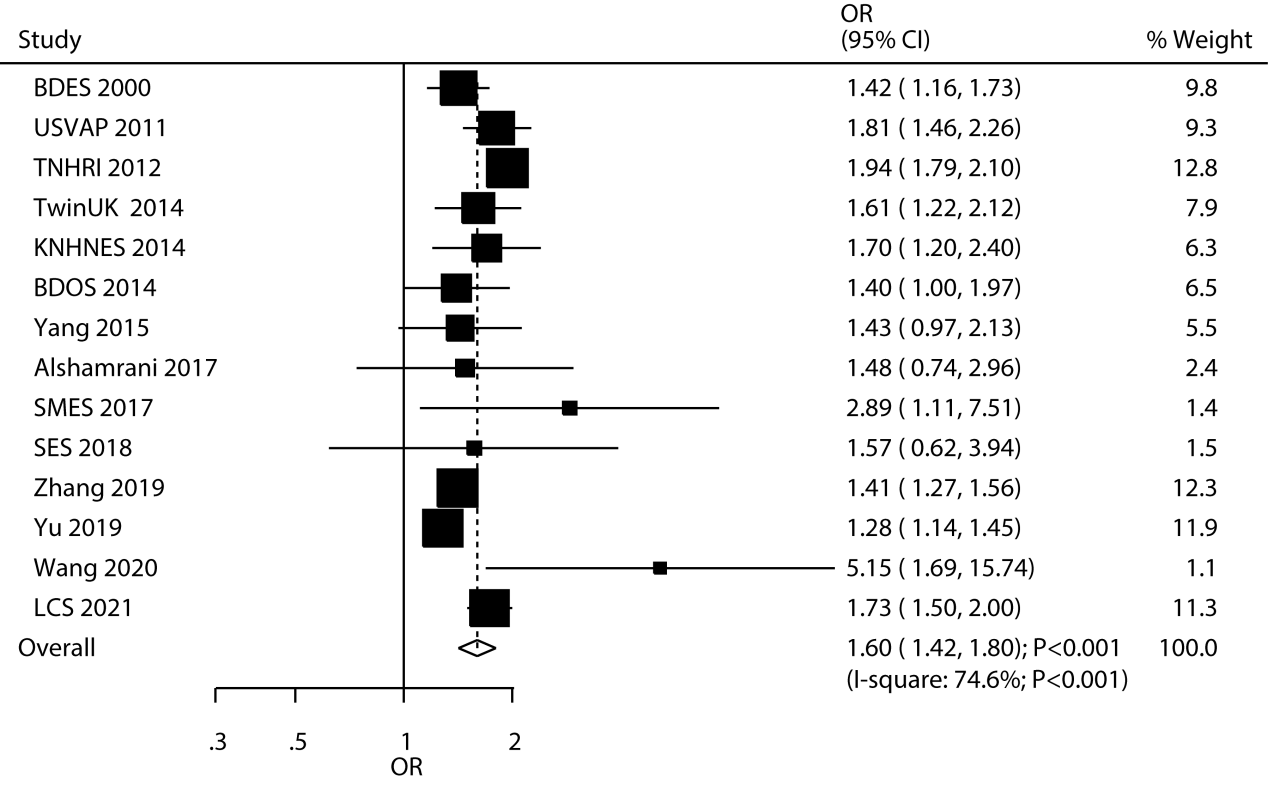


Figure S27. Association of thyroid disease with the risk of dry eye syndrome


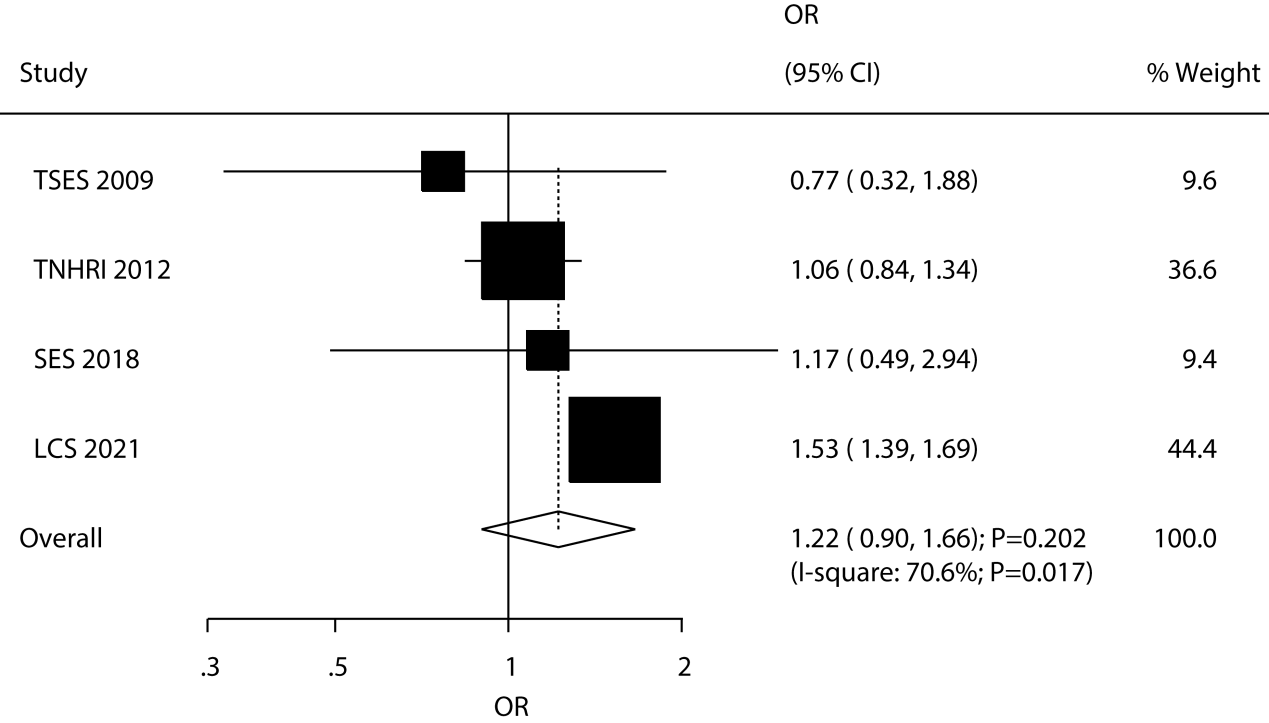


Figure S28. Association of COPD with the risk of dry eye syndrome


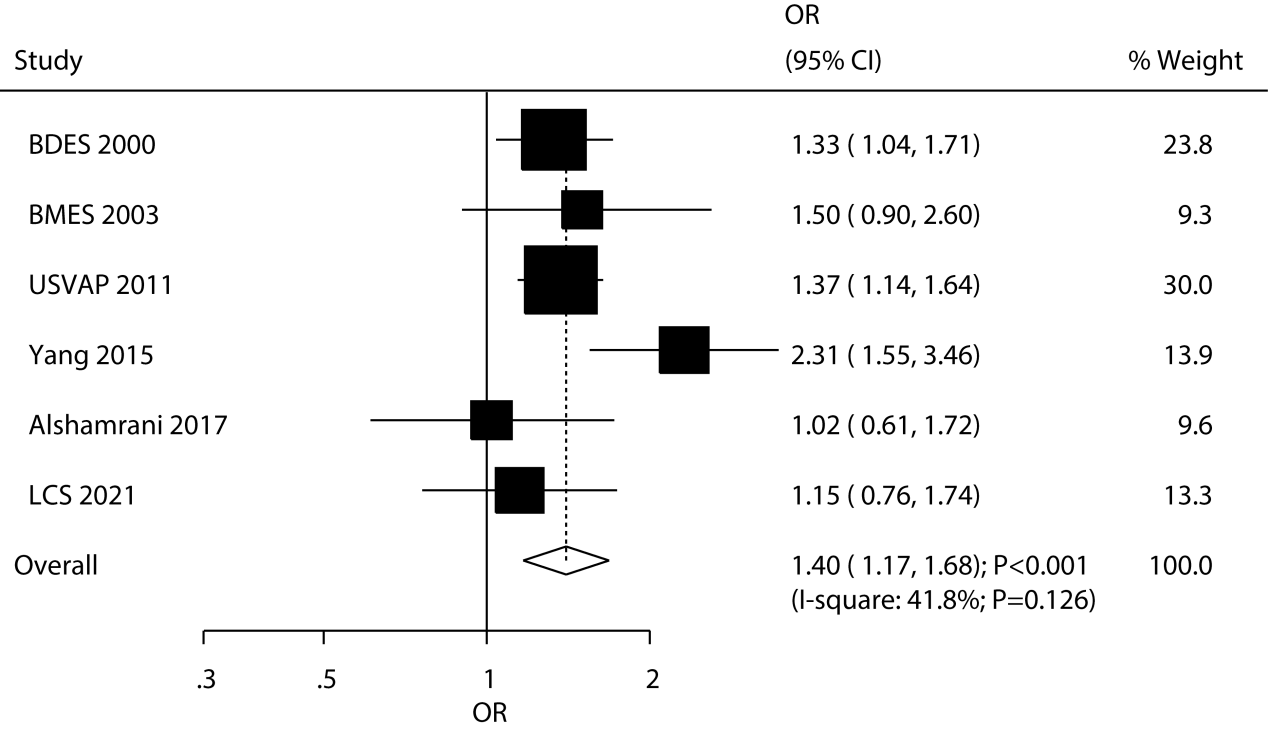


Figure S29. Association of gout with the risk of dry eye syndrome


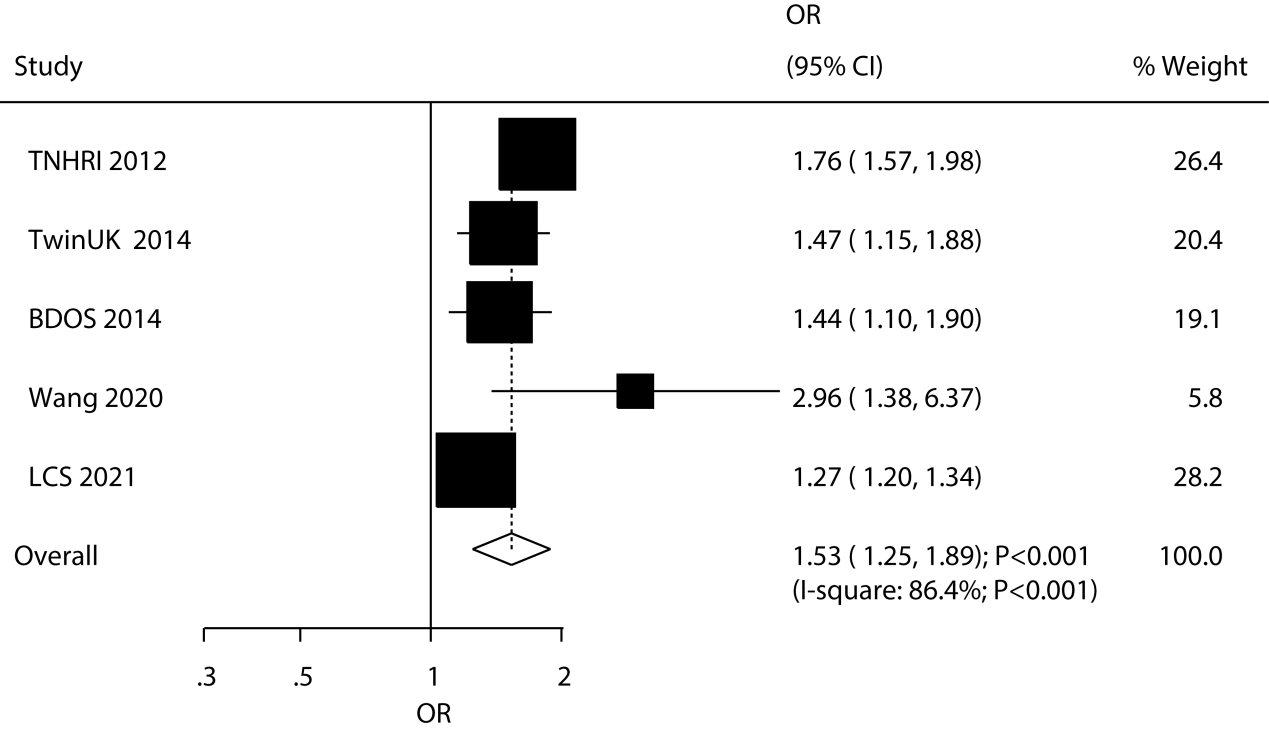


Figure S30. Association of migraines with the risk of dry eye syndrome


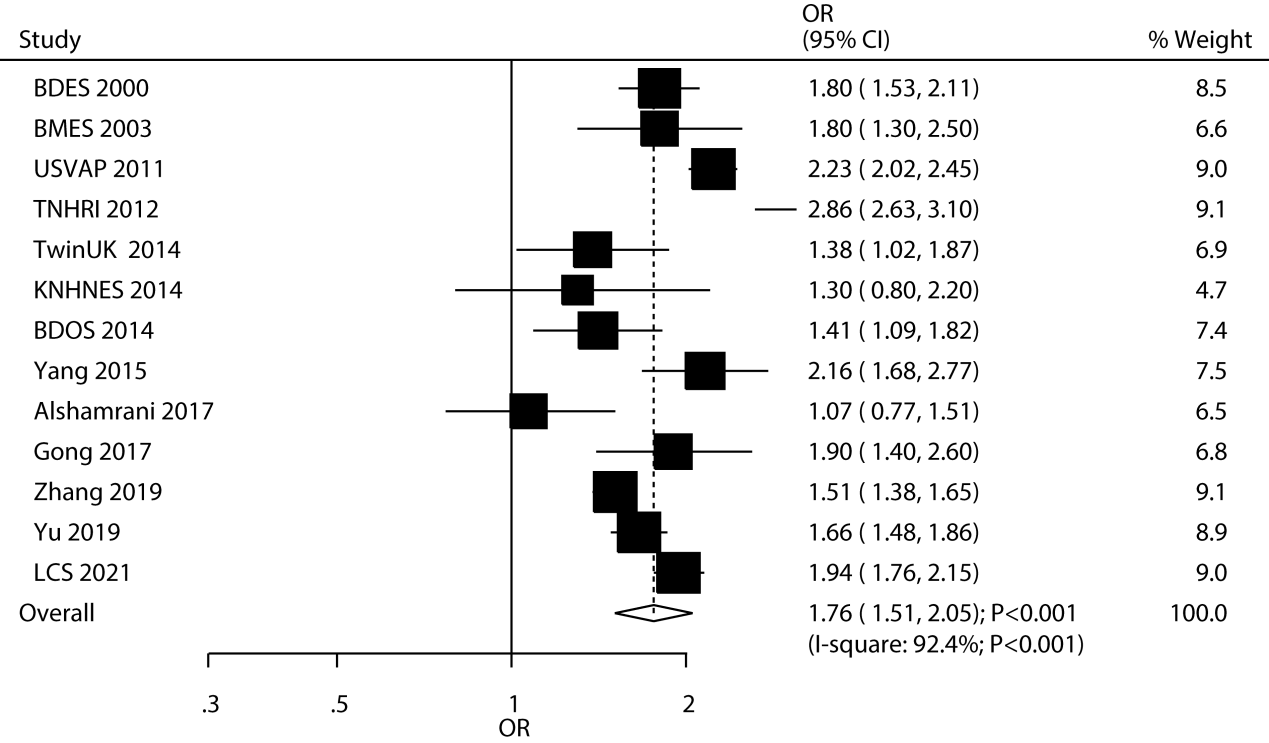


Figure S31. Association of arthritis with the risk of dry eye syndrome


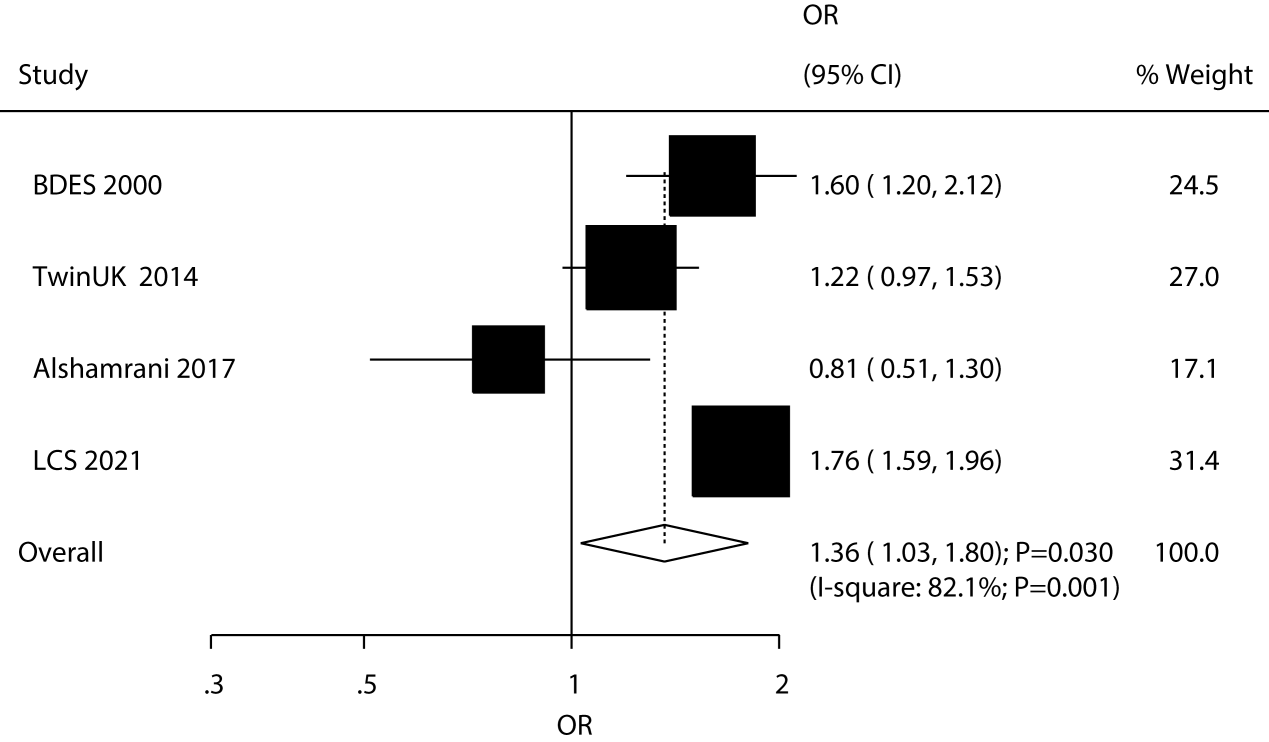


Figure S32. Association of osteoporosis with the risk of dry eye syndrome


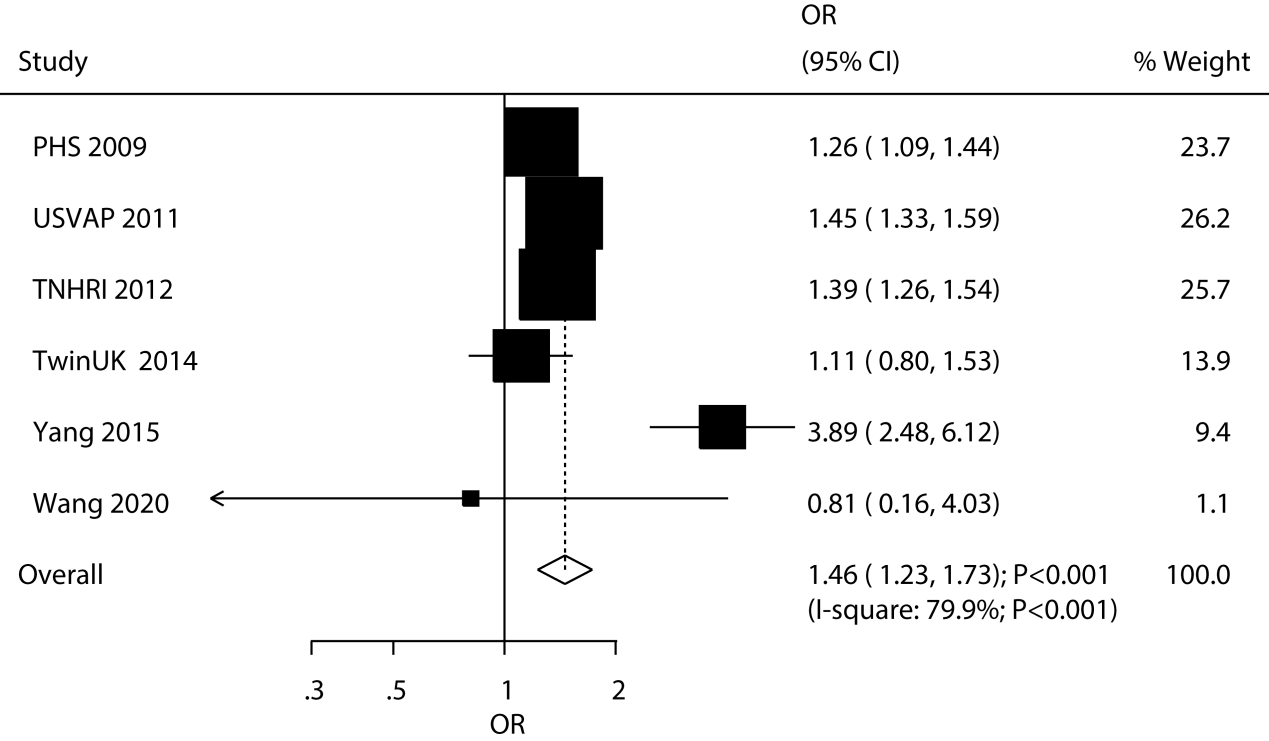


Figure S33. Association of tumor with the risk of dry eye syndrome


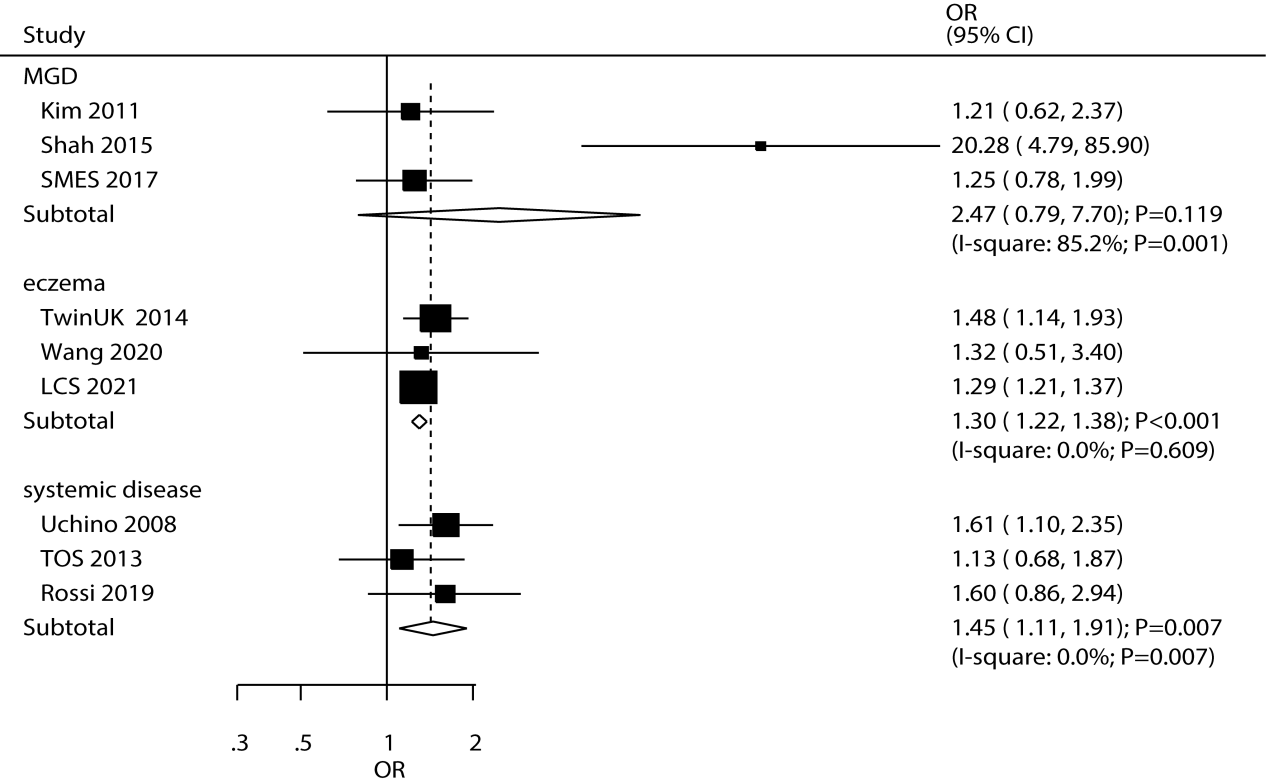


Figure S34. Association of MGD, eczema, and systemic disease with the risk of dry eye syndrome
