# Supplementary material for: Identified risk factors for dry eye syndrome: A systematic review and meta-analysis
Source: PLoS One. 2022 Aug 19;17(8):e0271267. doi: 10.1371/journal.pone.0271267 (PMC9390932; doi:10.1371/journal.pone.0271267)
Supplement: S3 File — (DOCX) [file pone.0271267.s005.docx]

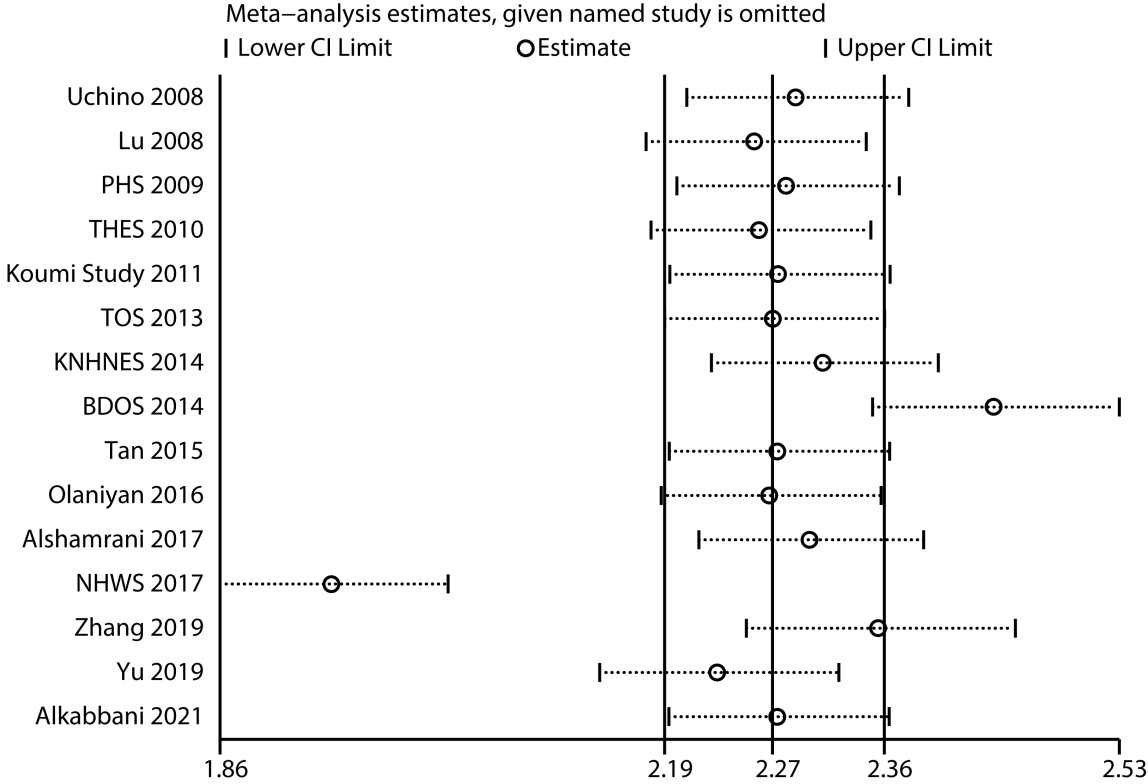


Figure S1. Sensitivity analysis for elderly versus younger on subsequent dry eye syndrome risk


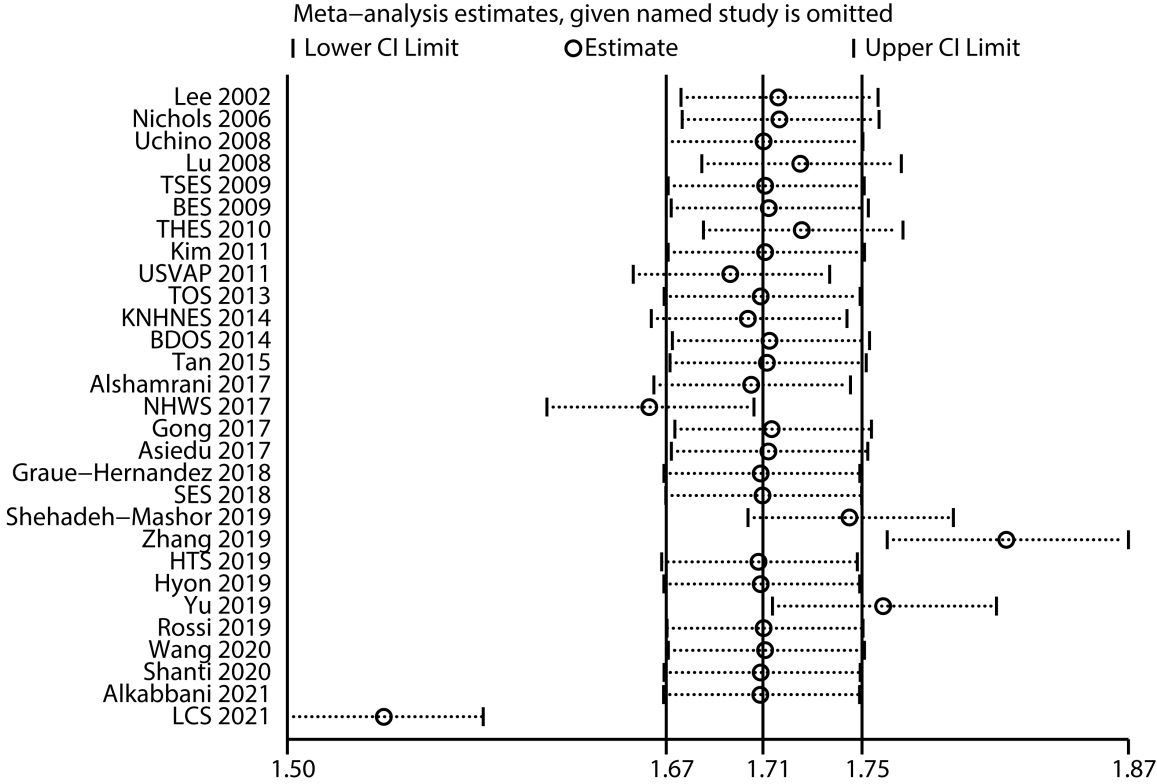


Figure S2. Sensitivity analysis for female versus male on subsequent dry eye syndrome risk


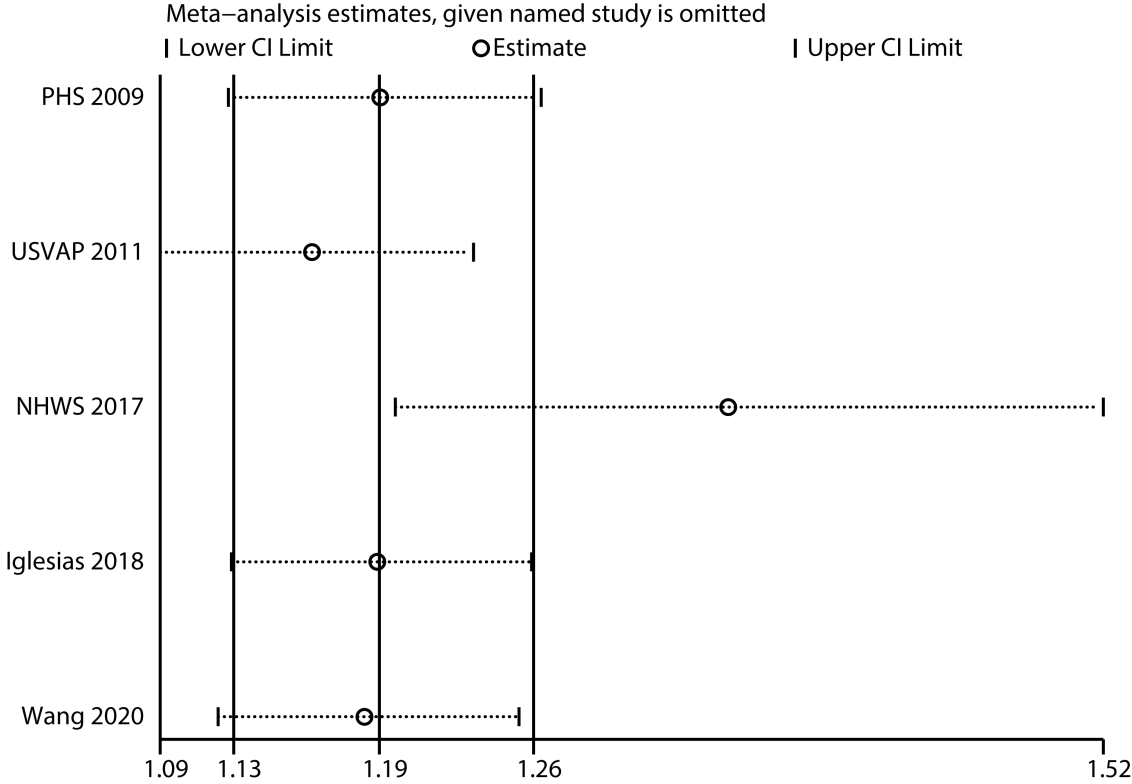


Figure S3. Sensitivity analysis for other race versus white on subsequent dry eye syndrome risk


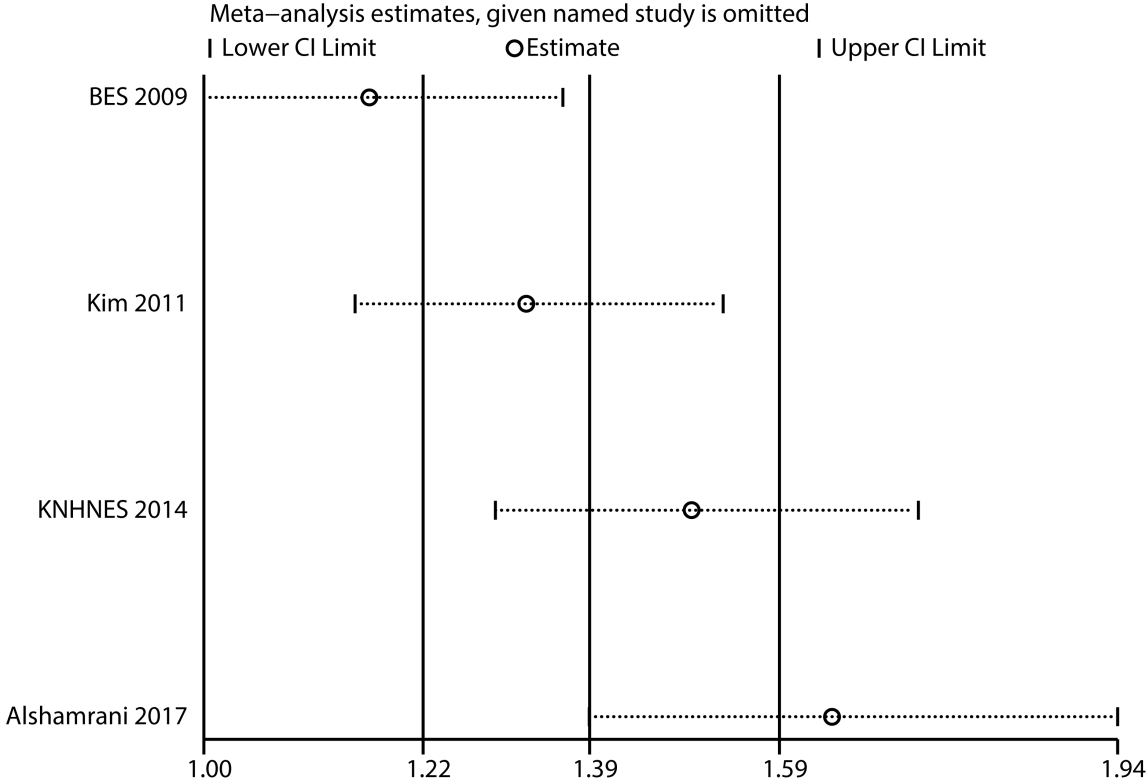


Figure S4. Sensitivity analysis for urban versus rural residence on subsequent dry eye syndrome risk


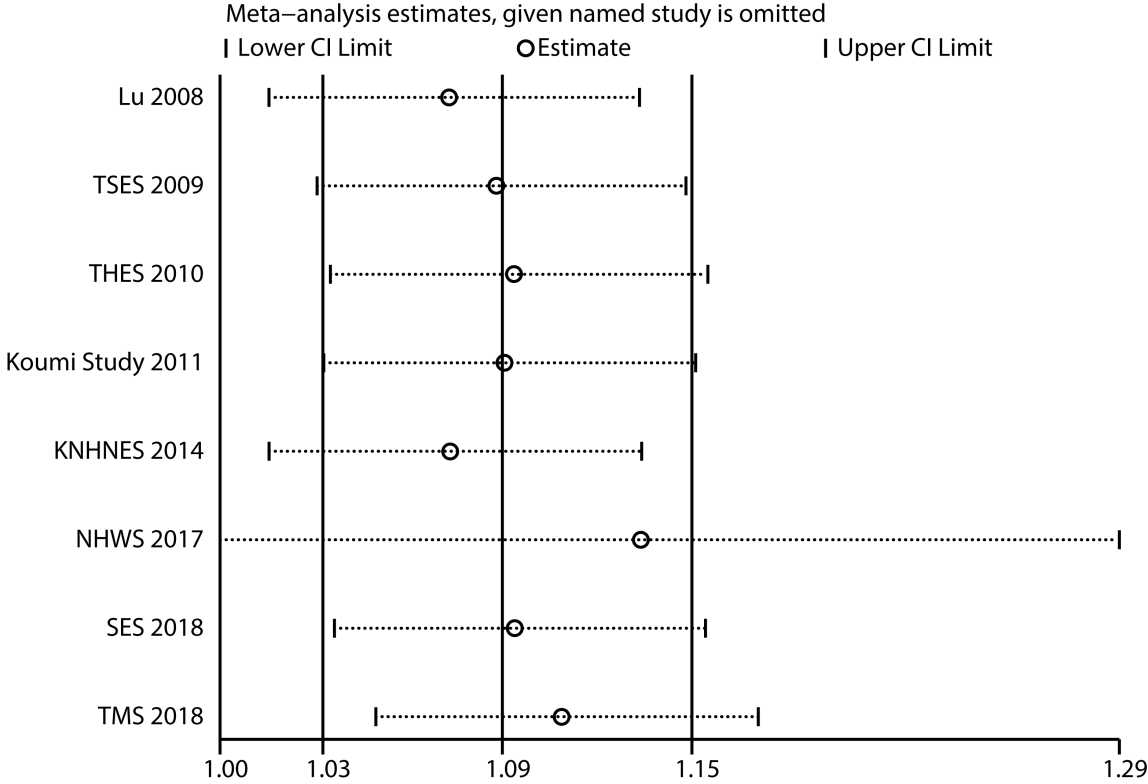


Figure S5. Sensitivity analysis for high versus low education level on subsequent dry eye syndrome risk


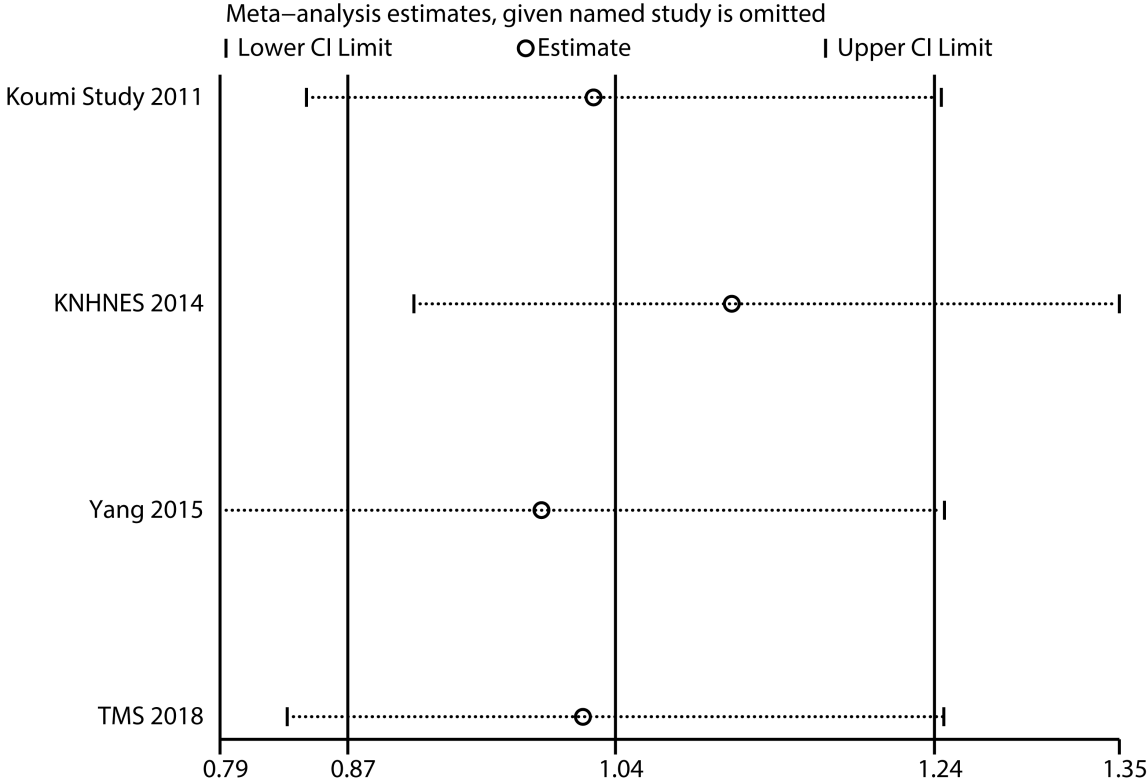


Figure S6. Sensitivity analysis for association of obesity with the risk of dry eye syndrome


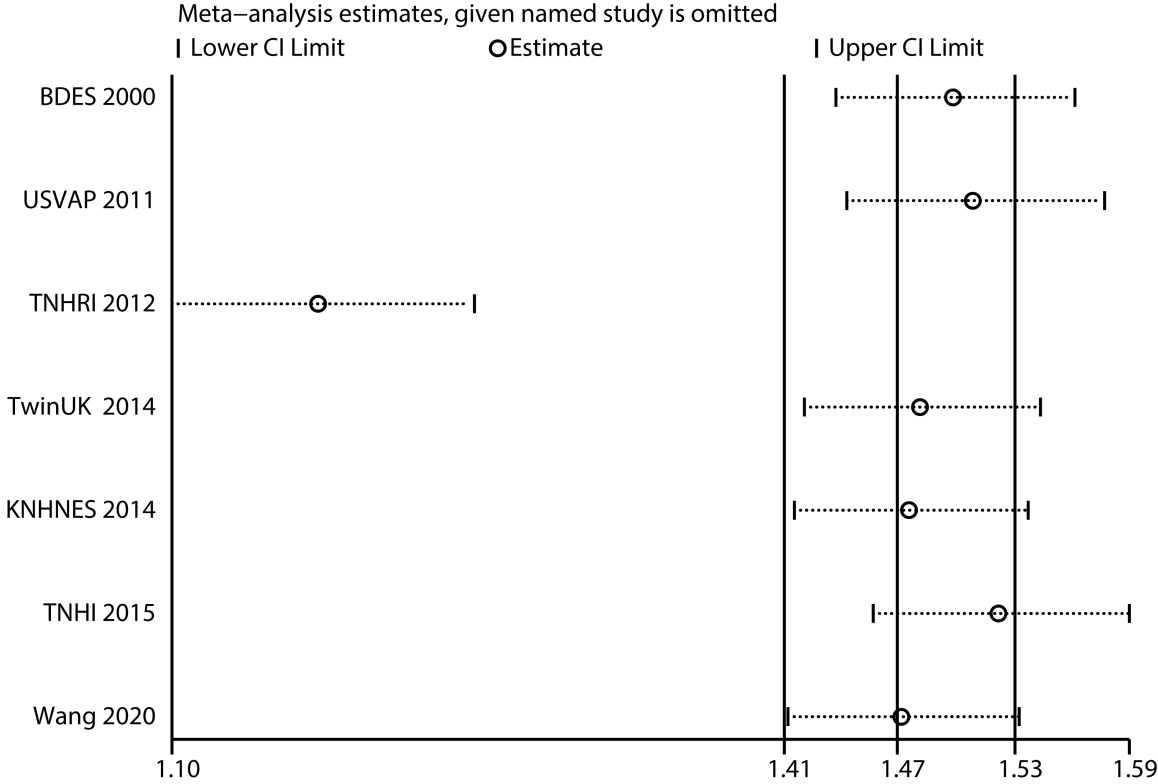


Figure S7. Sensitivity analysis for association of dyslipidemia with the risk of dry eye syndrome


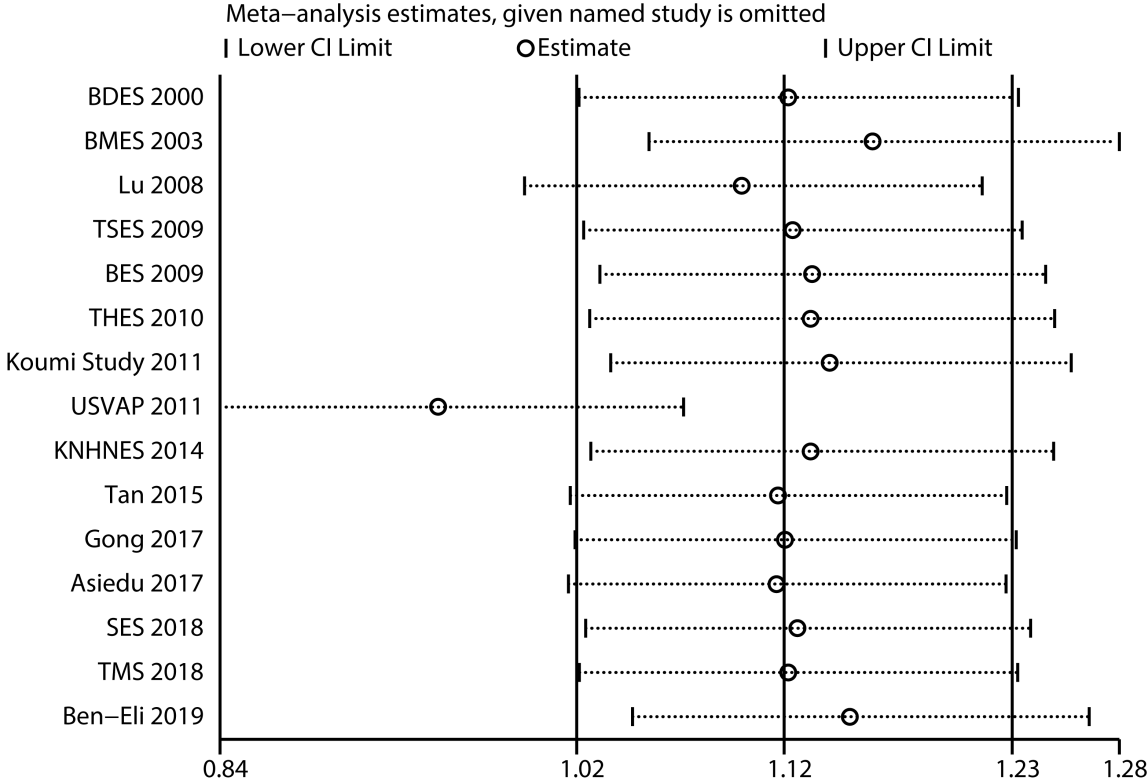


Figure S8. Sensitivity analysis for alcohol intake on subsequent risk of dry eye syndrome


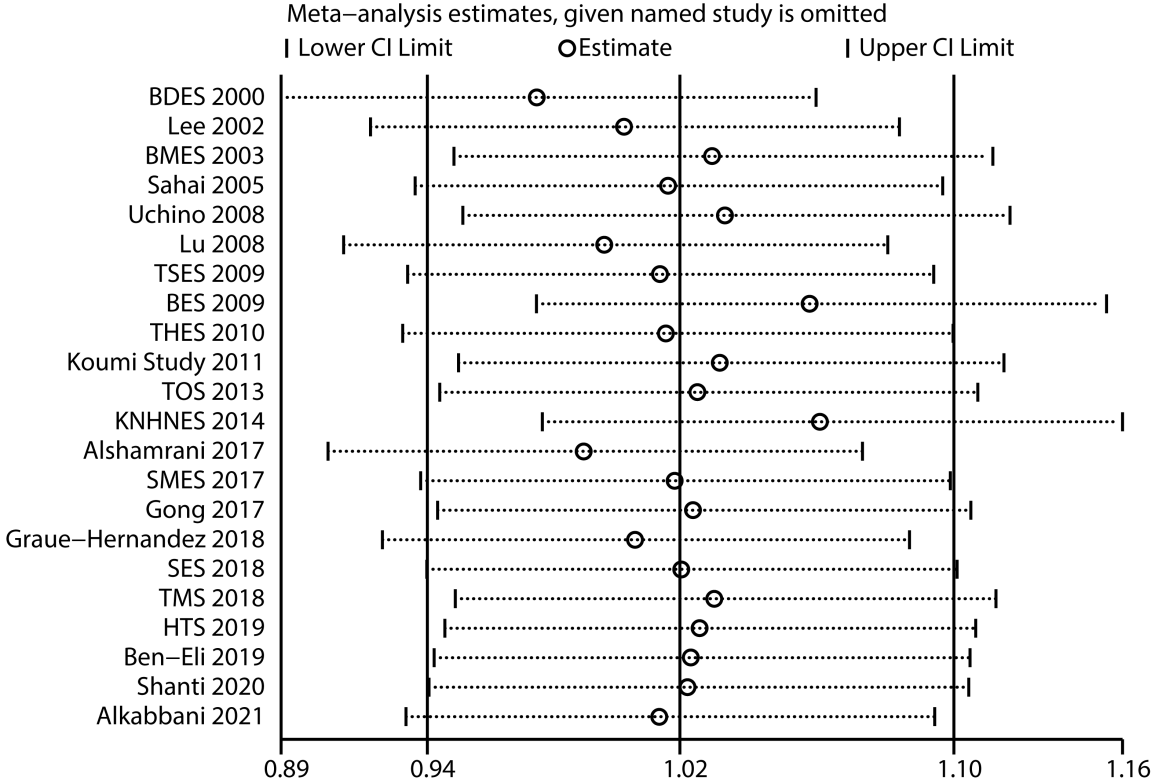


Figure S9. Sensitivity analysis for association of smoking with the risk of dry eye syndrome


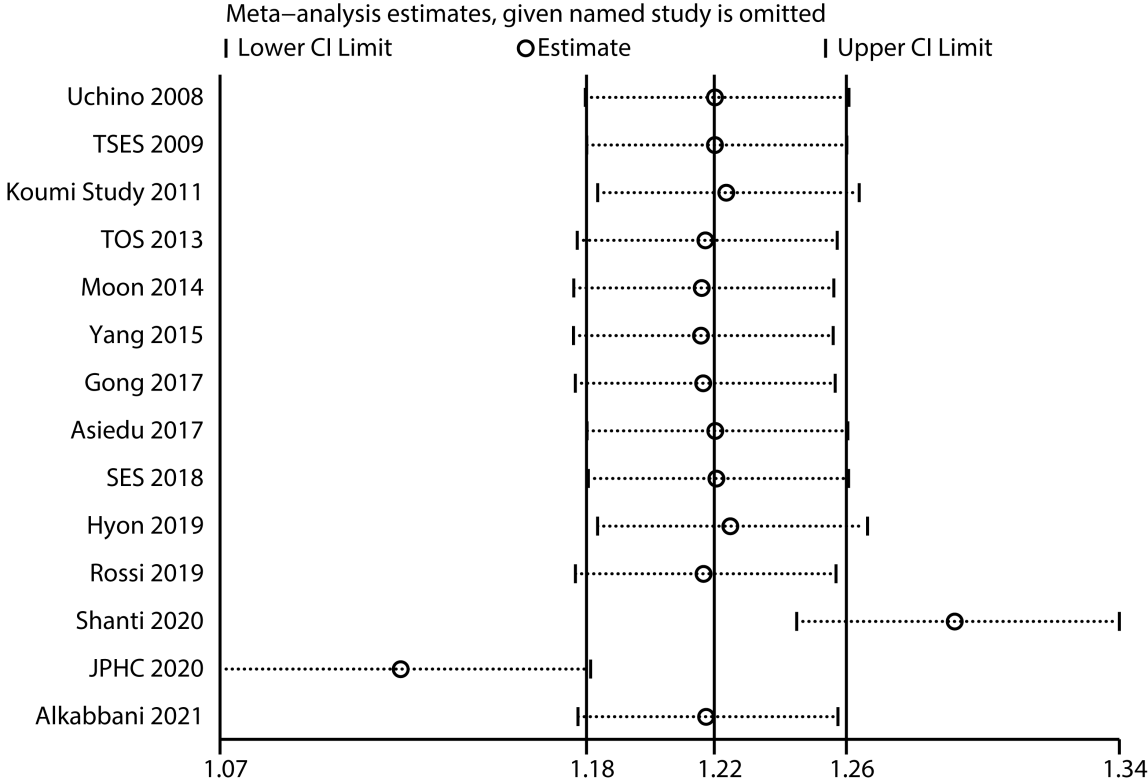


Figure S10. Sensitivity analysis for association of VDT use with the risk of dry eye syndrome


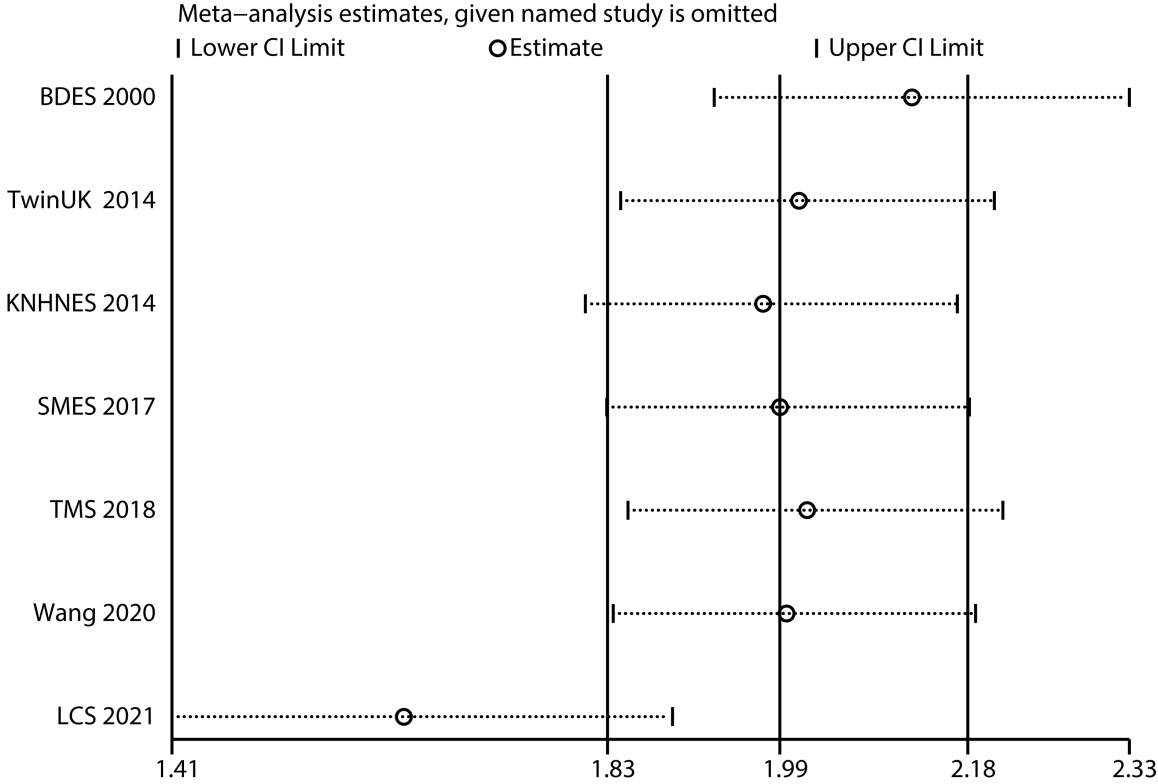


Figure S11. Sensitivity analysis for association of cataract surgery with the risk of dry eye syndrome


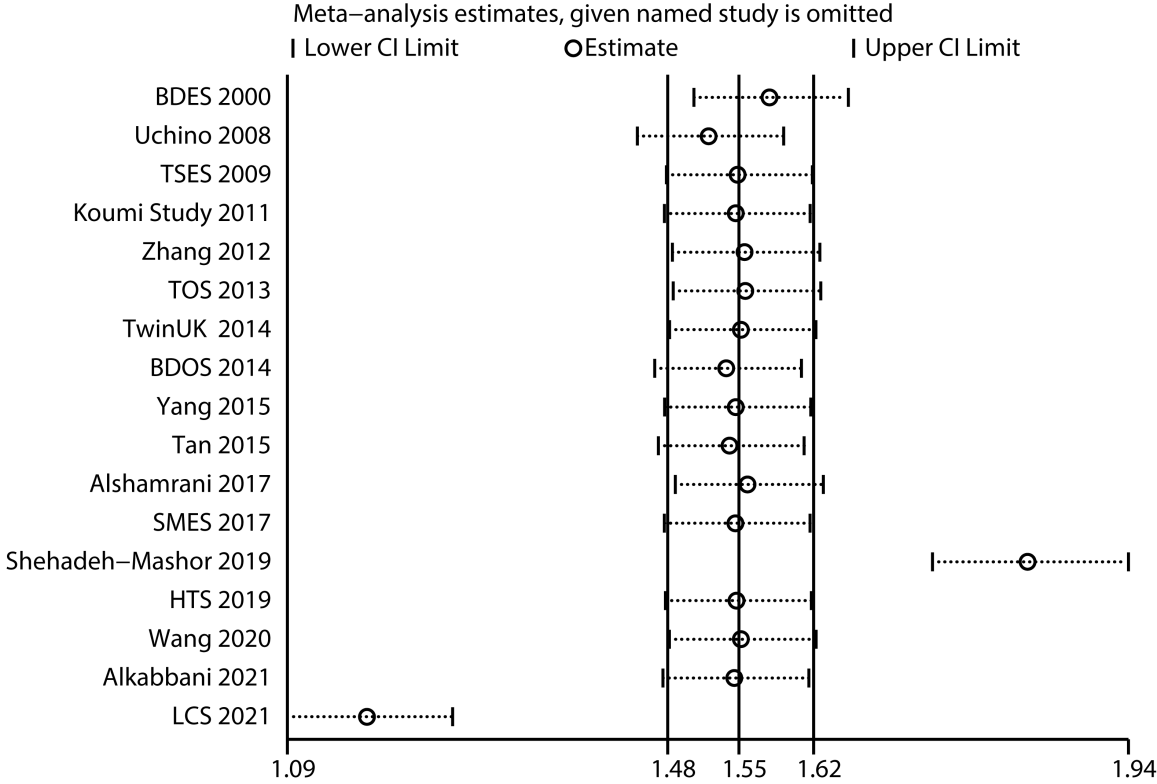


Figure S12. Sensitivity analysis for association of contact lens wear with the risk of dry eye syndrome


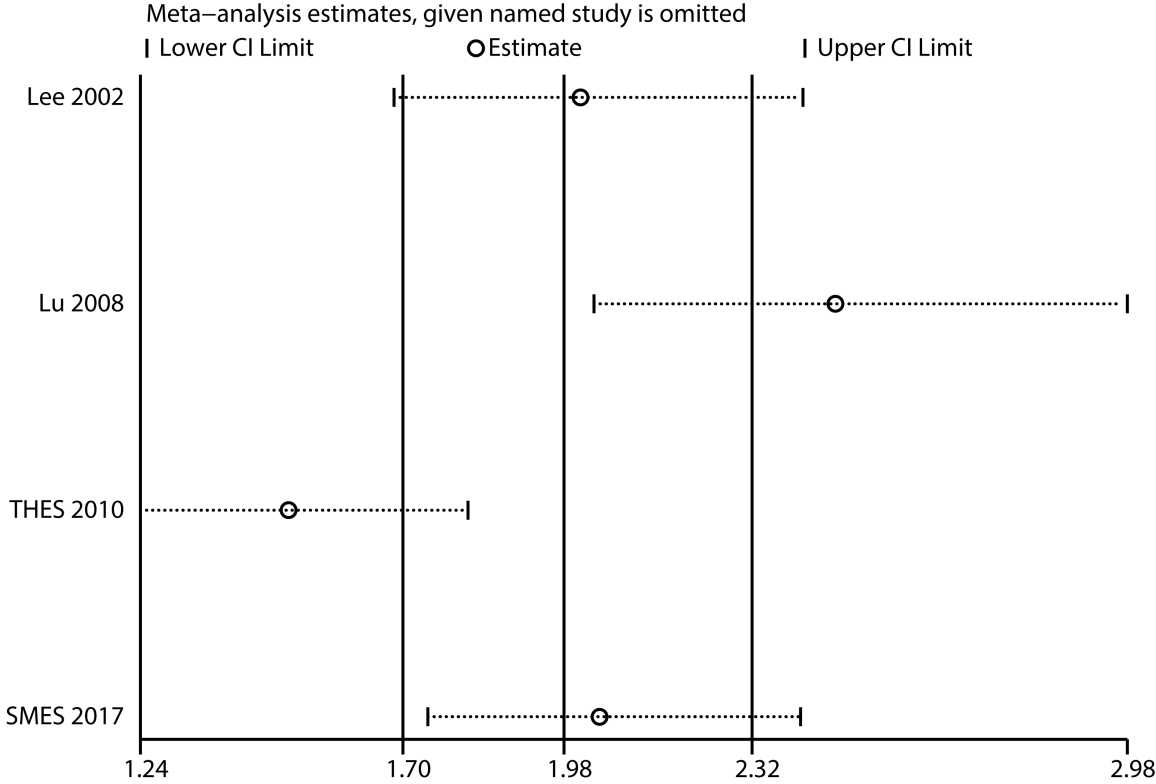


Figure S13. Sensitivity analysis for association of pterygium with the risk of dry eye syndrome


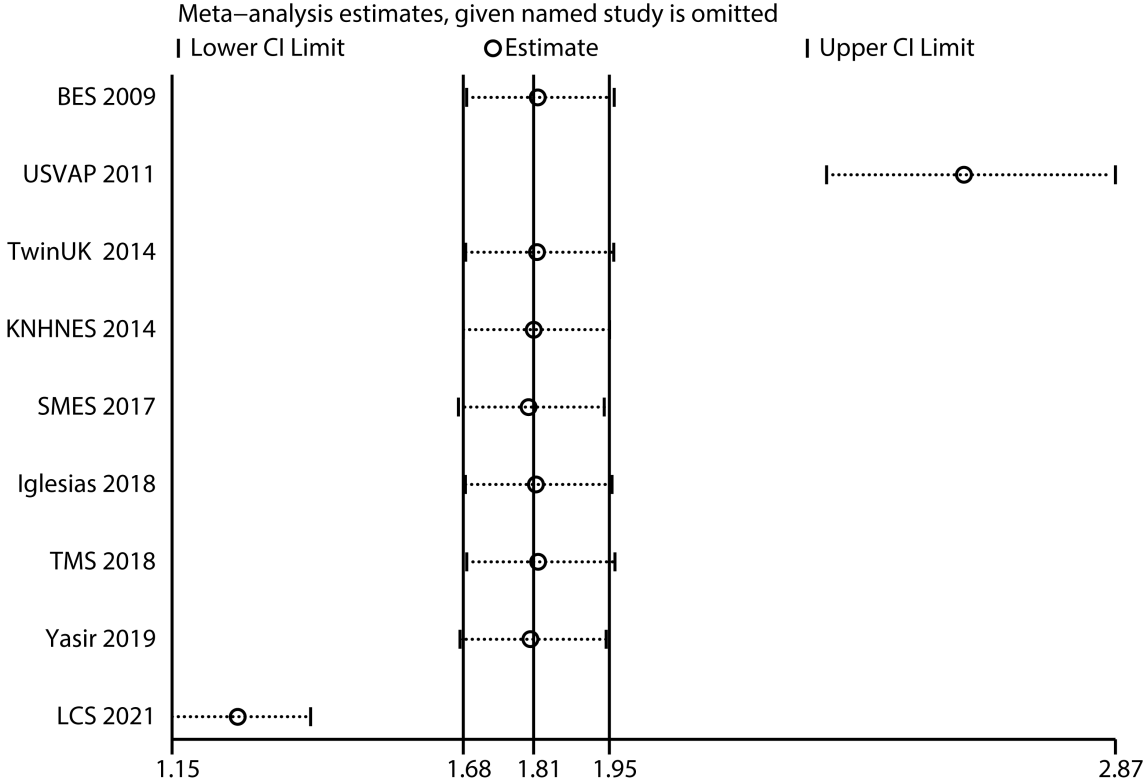


Figure S14. Sensitivity analysis for association of glaucoma with the risk of dry eye syndrome


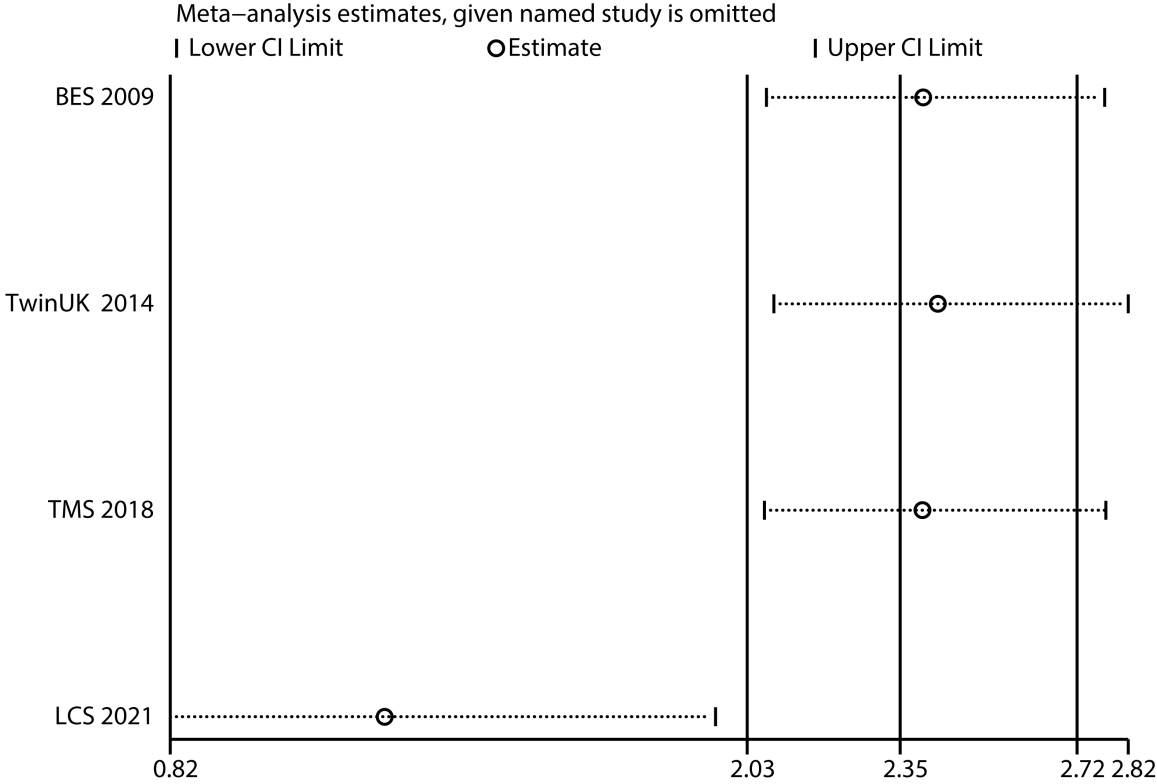


Figure S15. Sensitivity analysis for association of age-related maculopathy with the risk of dry eye syndrome


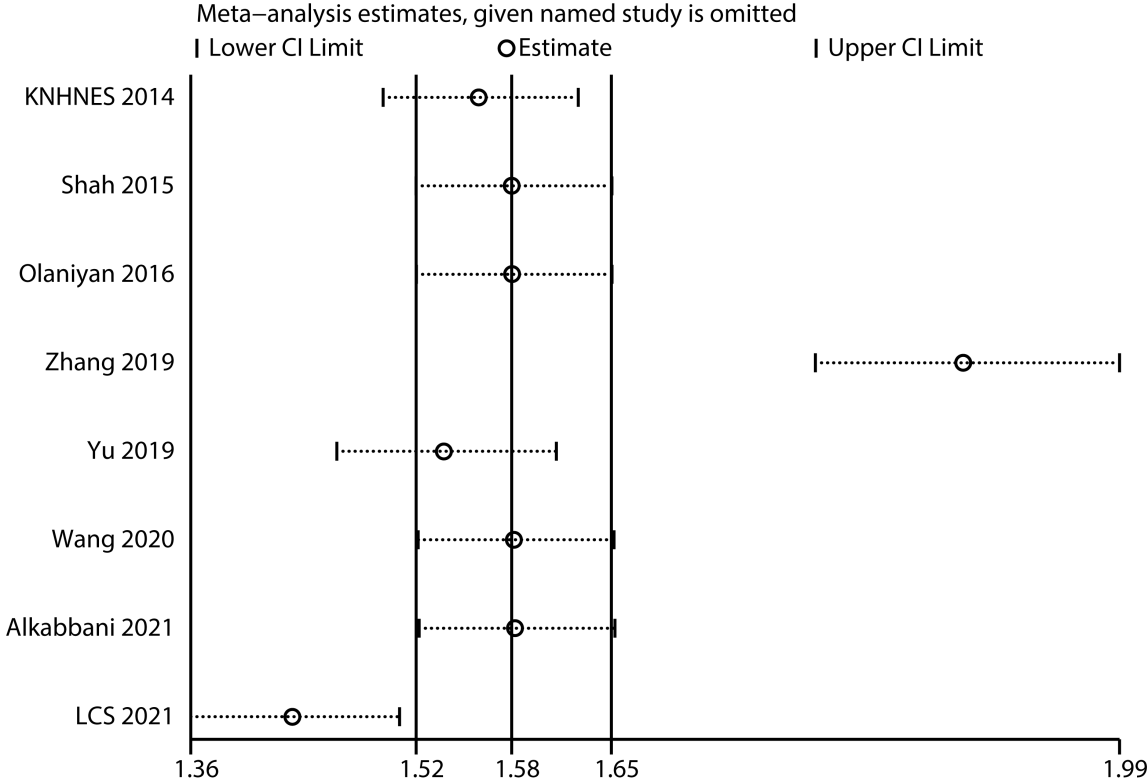


Figure S16. Sensitivity analysis for association of eye surgery with the risk of dry eye syndrome


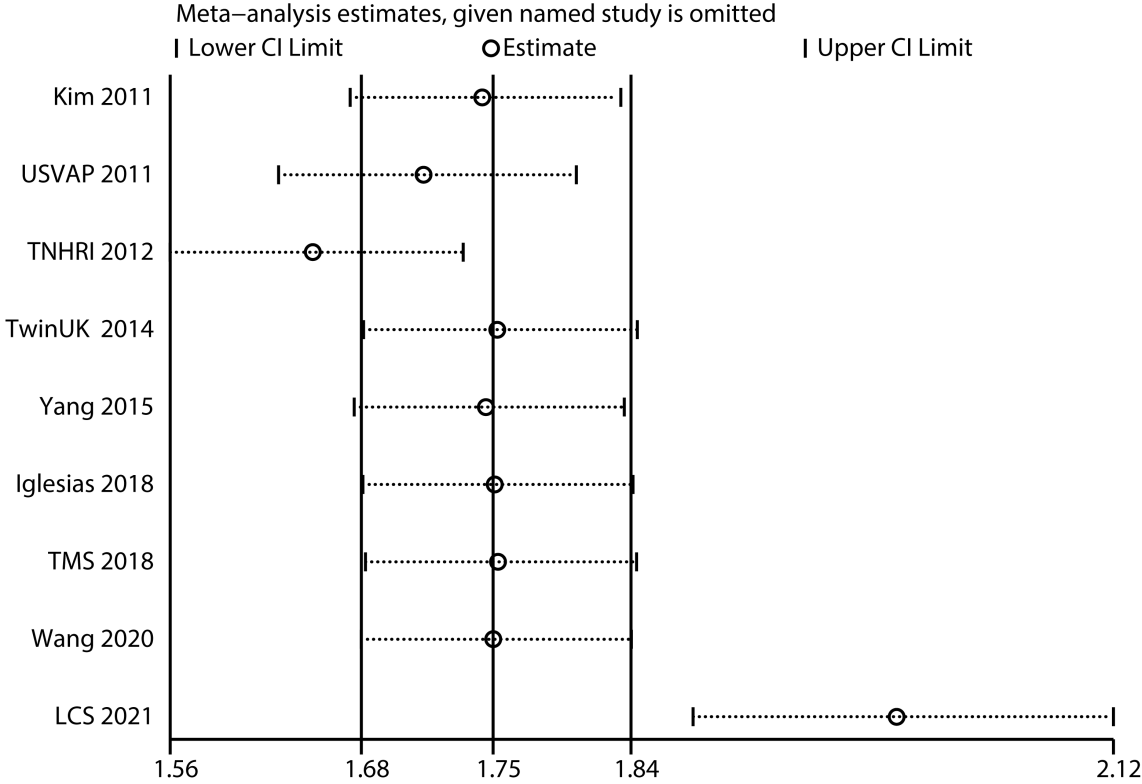


Figure S17. Sensitivity analysis for association of depression with the risk of dry eye syndrome


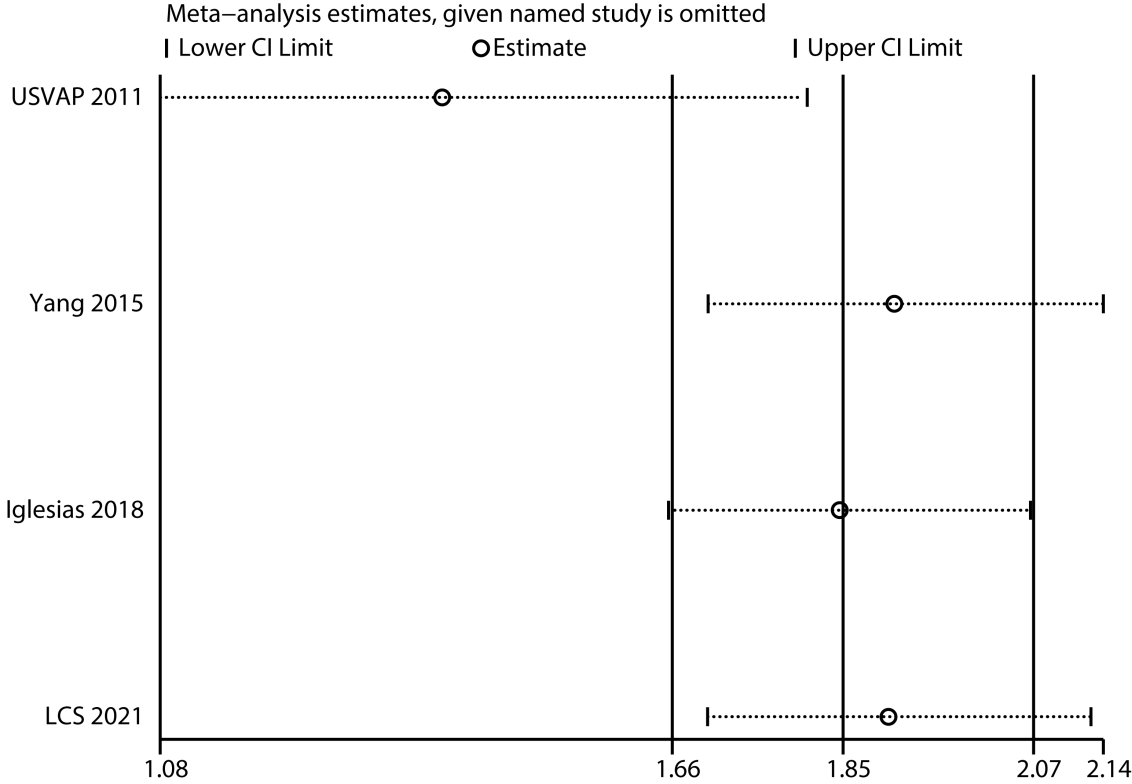


Figure S18. Sensitivity analysis for association of PTSD with the risk of dry eye syndrome


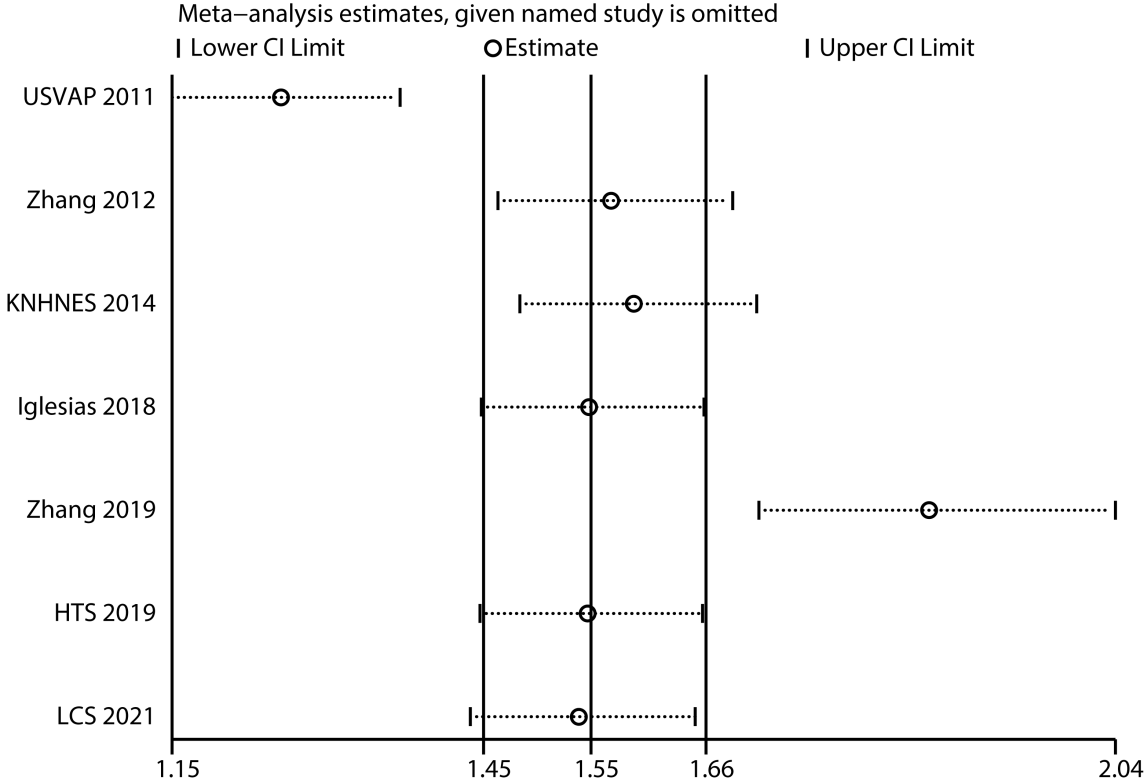


Figure S19. Sensitivity analysis for association of sleep apnea with the risk of dry eye syndrome


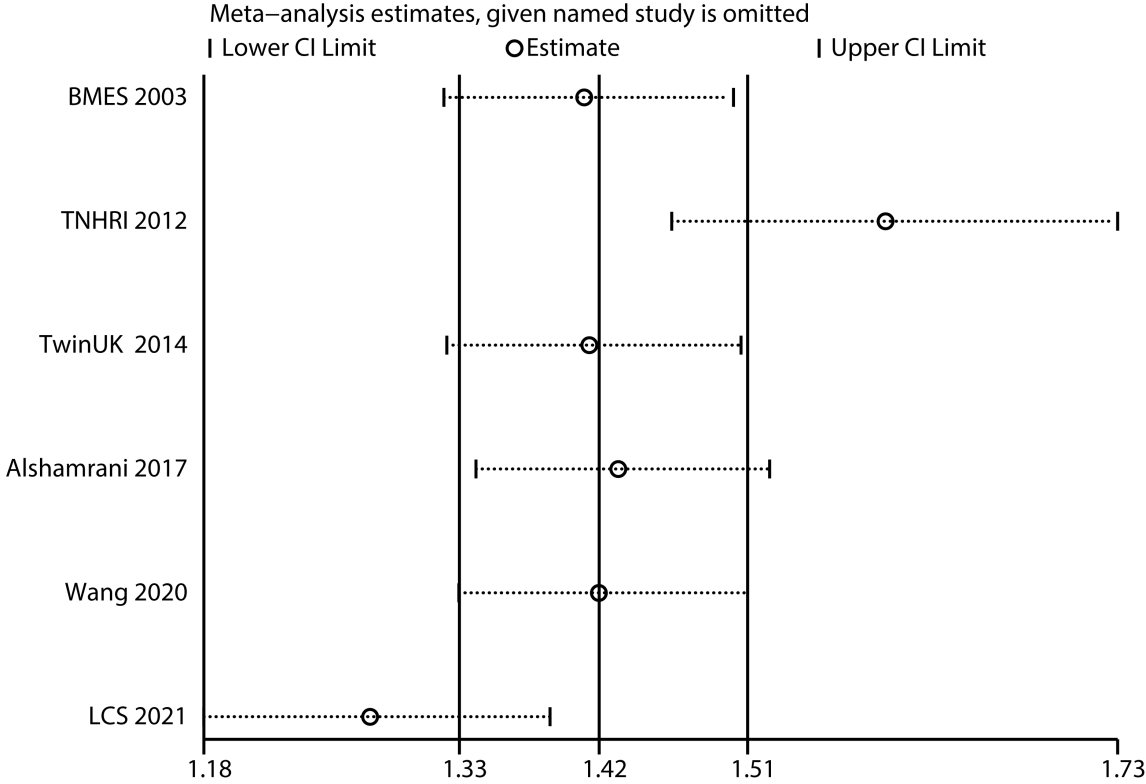


Figure S20. Sensitivity analysis for association of asthma with the risk of dry eye syndrome


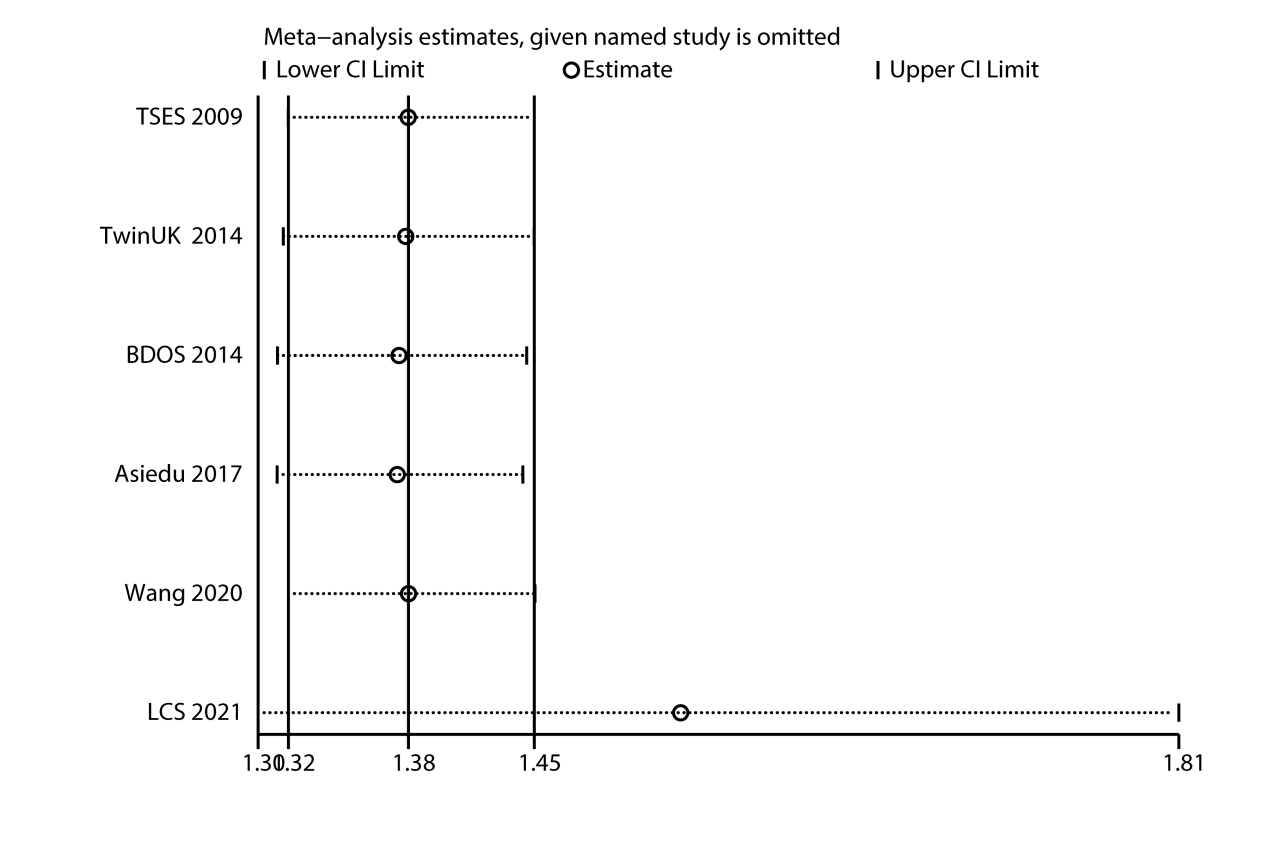


Figure S21. Sensitivity analysis for association of allergy with the risk of dry eye syndrome


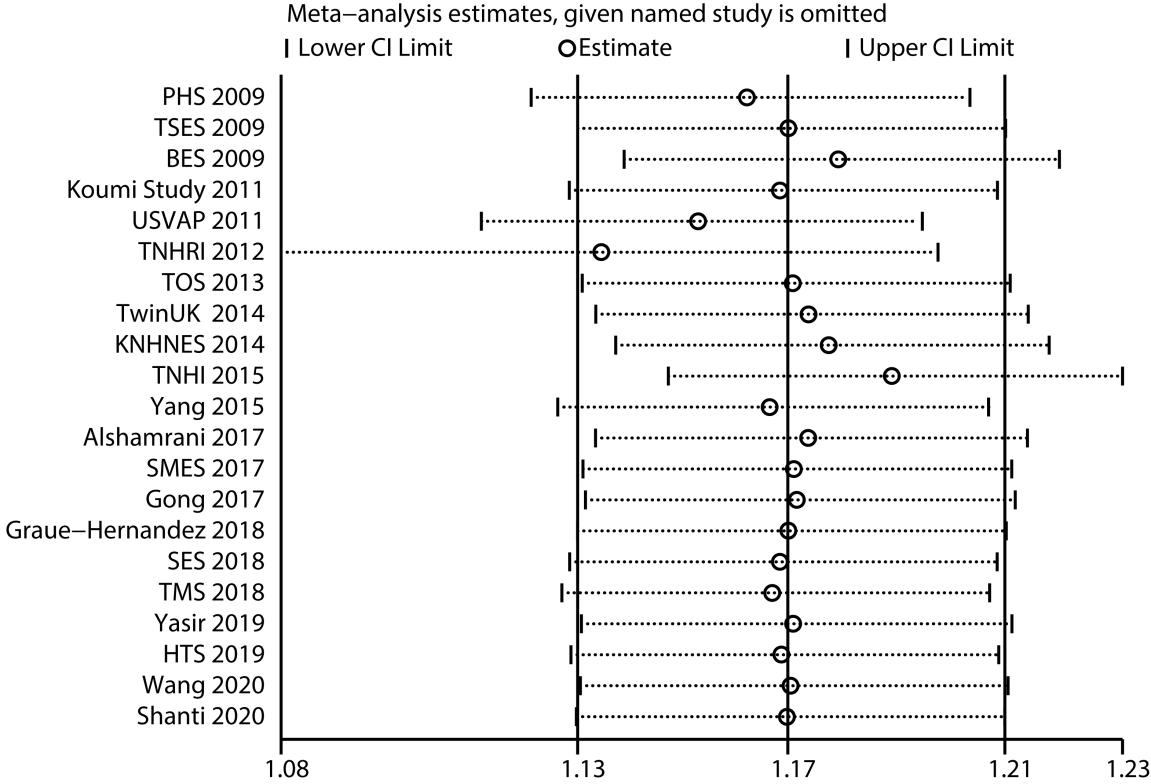


Figure S22. Sensitivity analysis for association of hypertension with the risk of dry eye syndrome


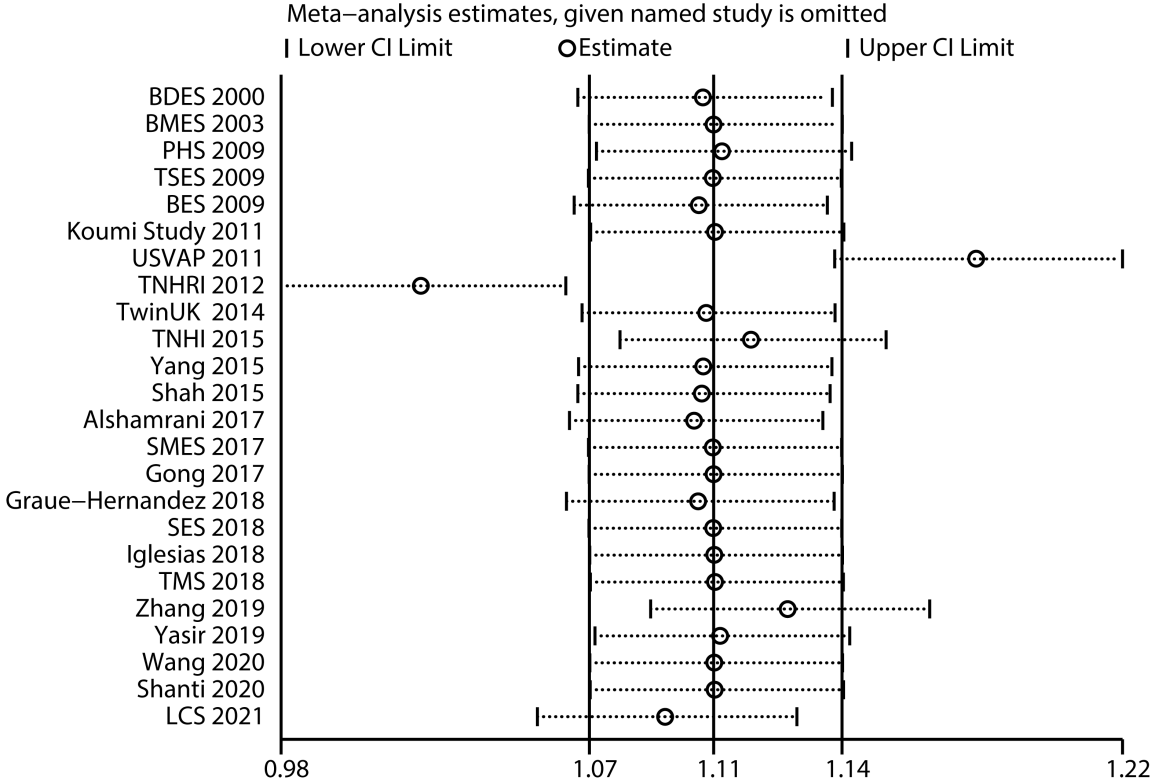


Figure S23. Sensitivity analysis for association of DM with the risk of dry eye syndrome


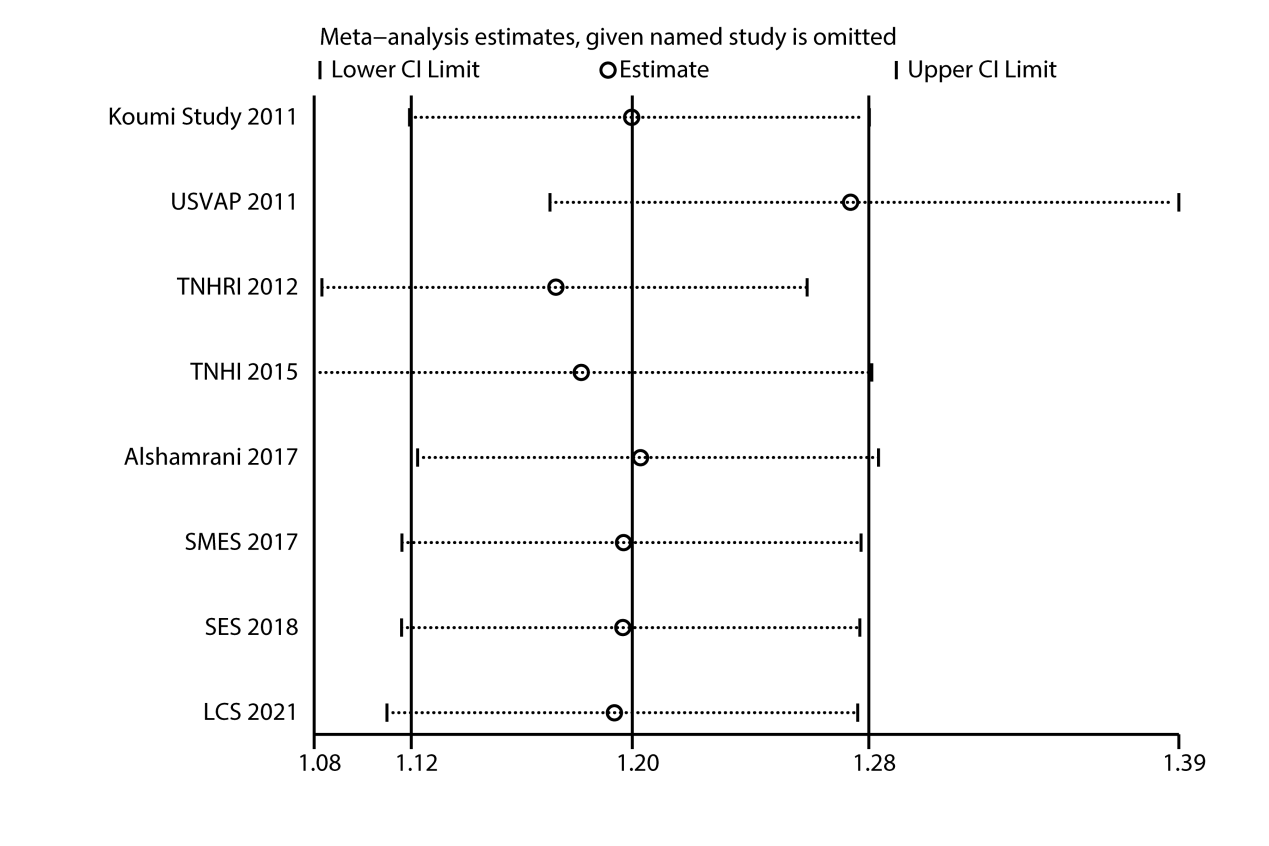


Figure S24. Sensitivity analysis for association of CVD with the risk of dry eye syndrome


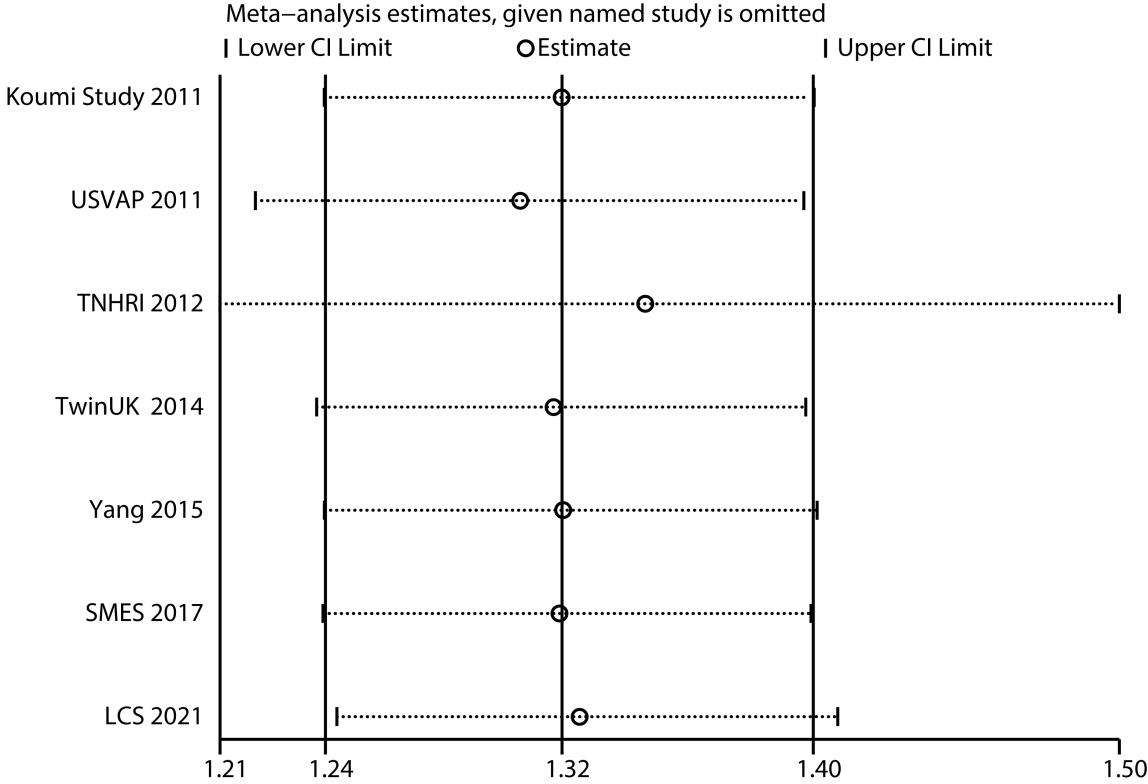


Figure S25. Sensitivity analysis for association of stroke with the risk of dry eye syndrome


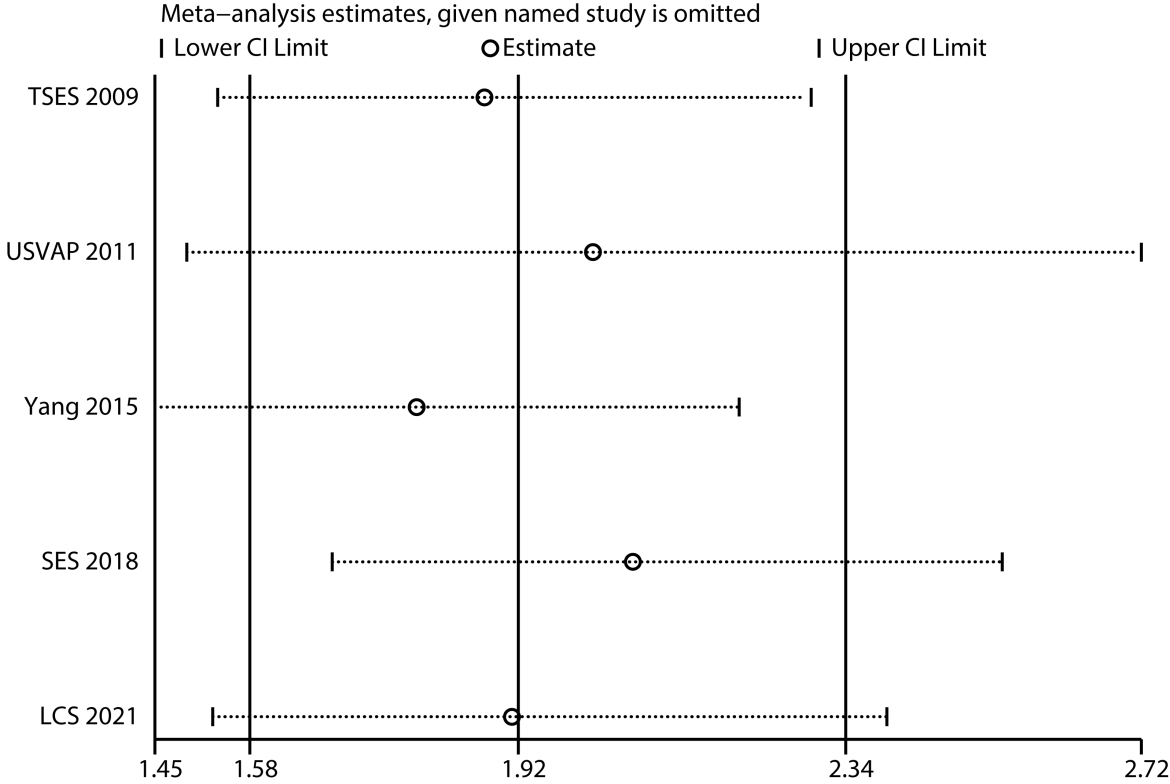


Figure S26. Sensitivity analysis for association of rosacea with the risk of dry eye syndrome


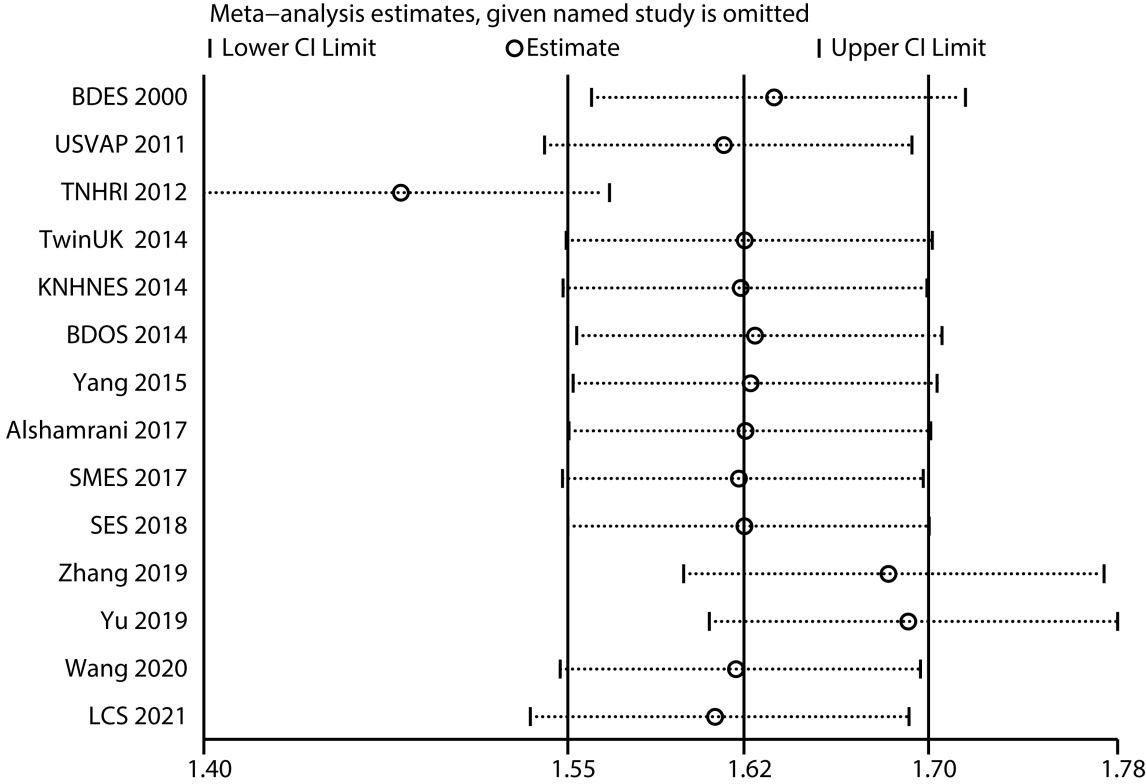


Figure S27. Sensitivity analysis for association of thyroid disease with the risk of dry eye syndrome


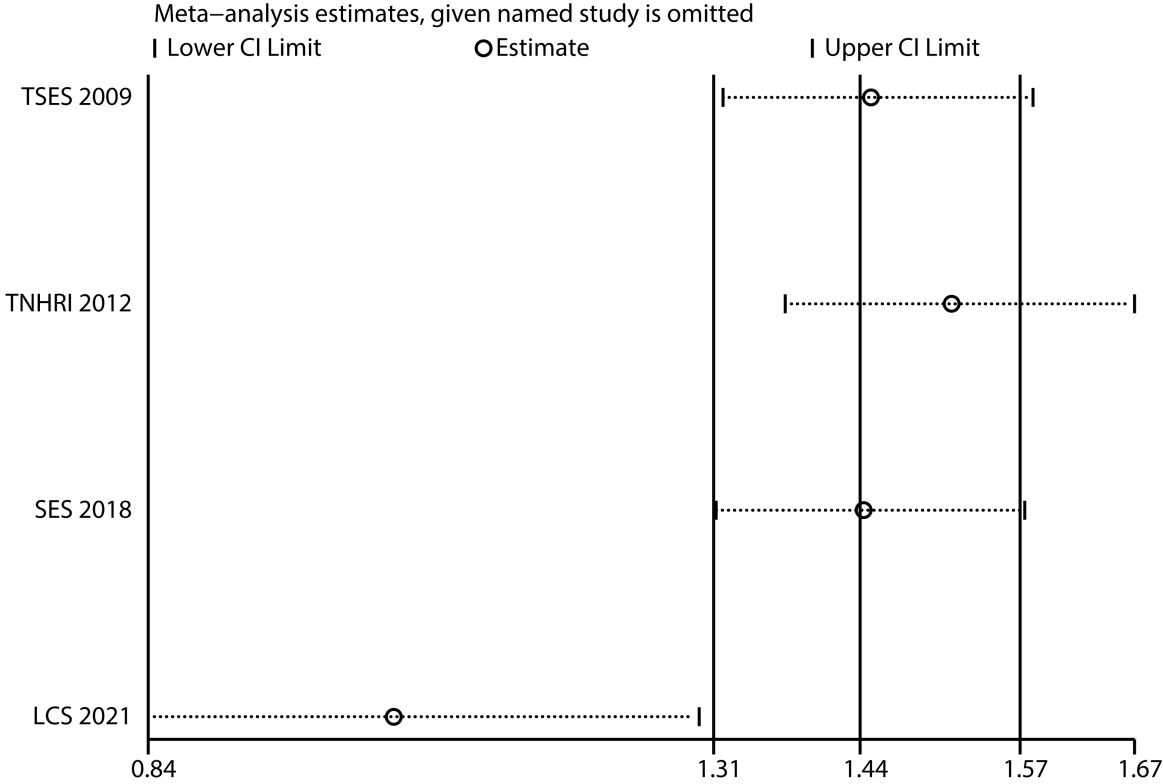


Figure S28. Sensitivity analysis for association of COPD with the risk of dry eye syndrome


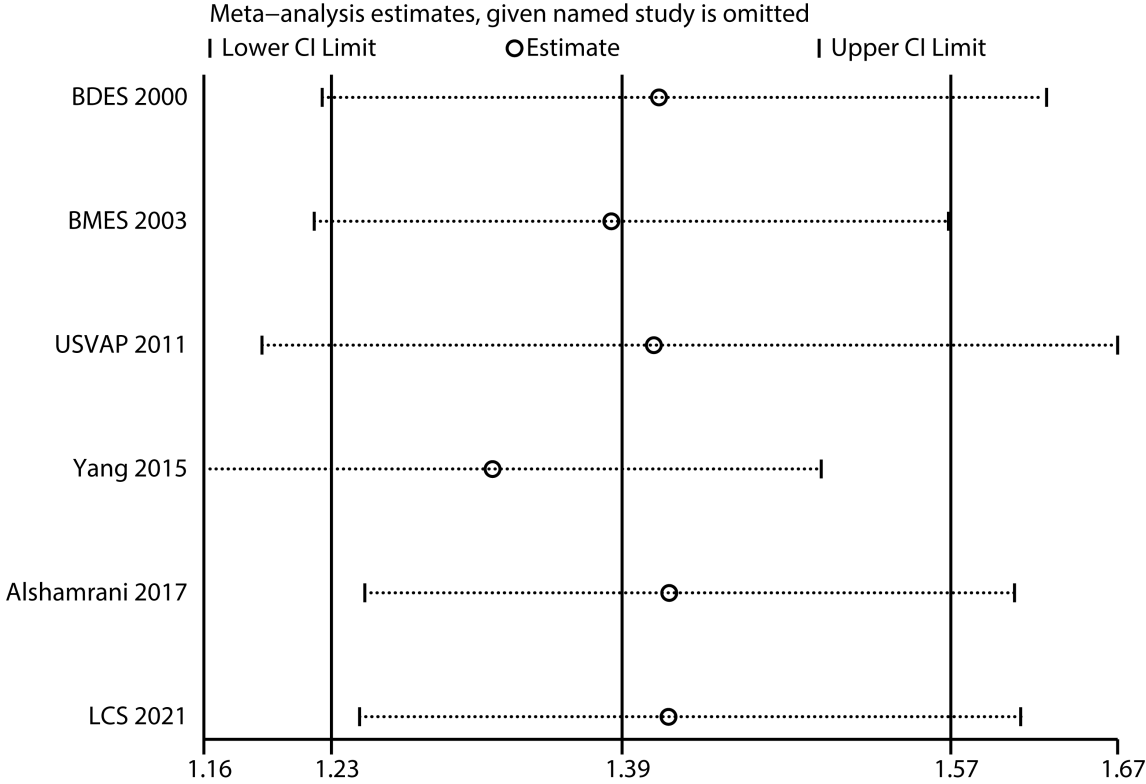


Figure S29. Sensitivity analysis for association of gout with the risk of dry eye syndrome


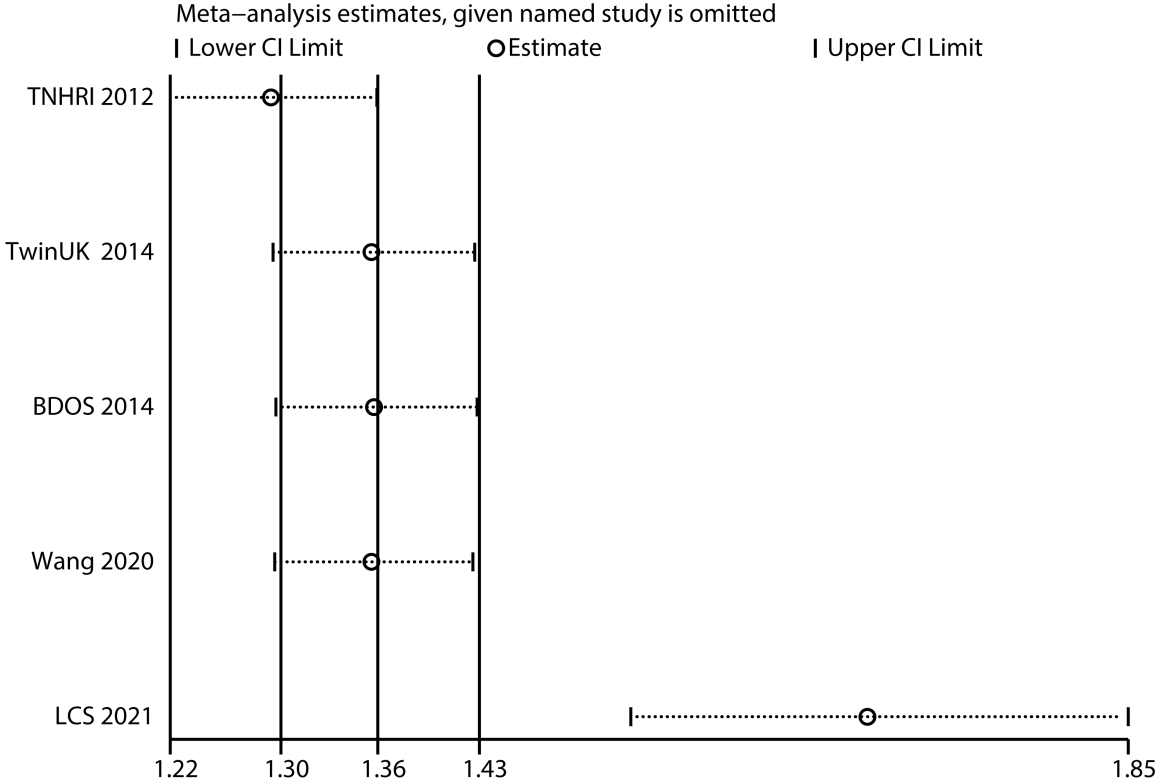


Figure S30. Sensitivity analysis for association of migraines with the risk of dry eye syndrome


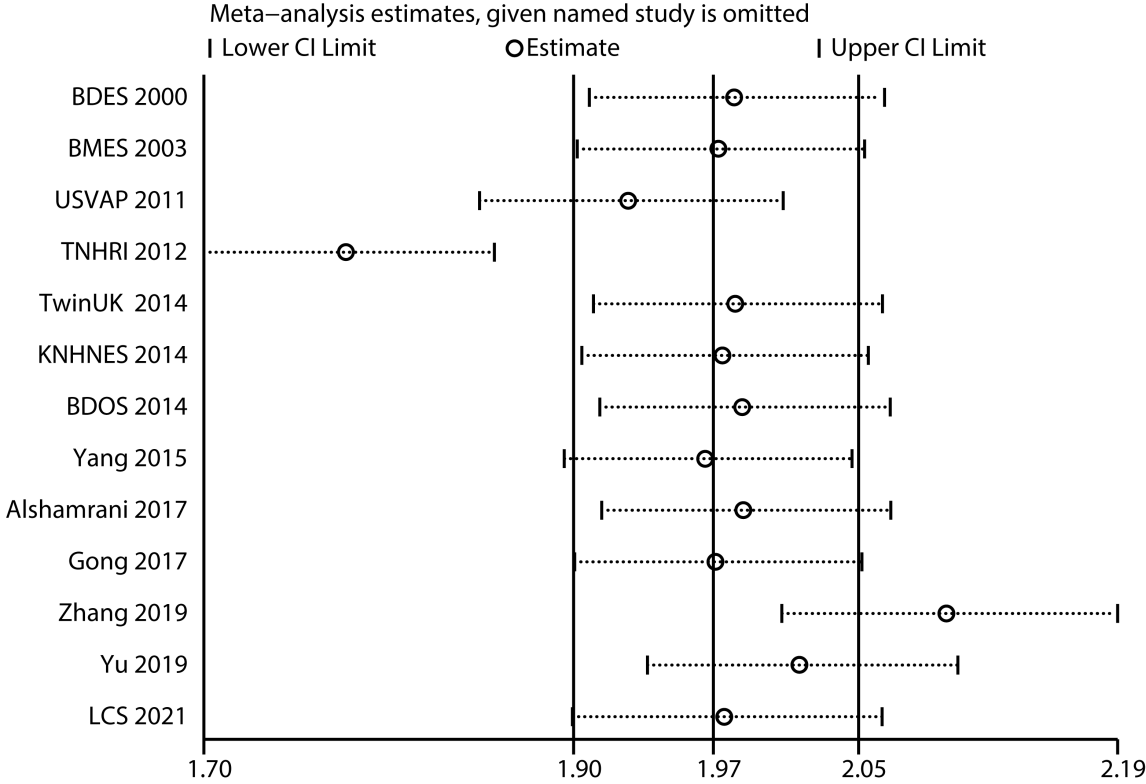


Figure S31. Sensitivity analysis for association of arthritis with the risk of dry eye syndrome


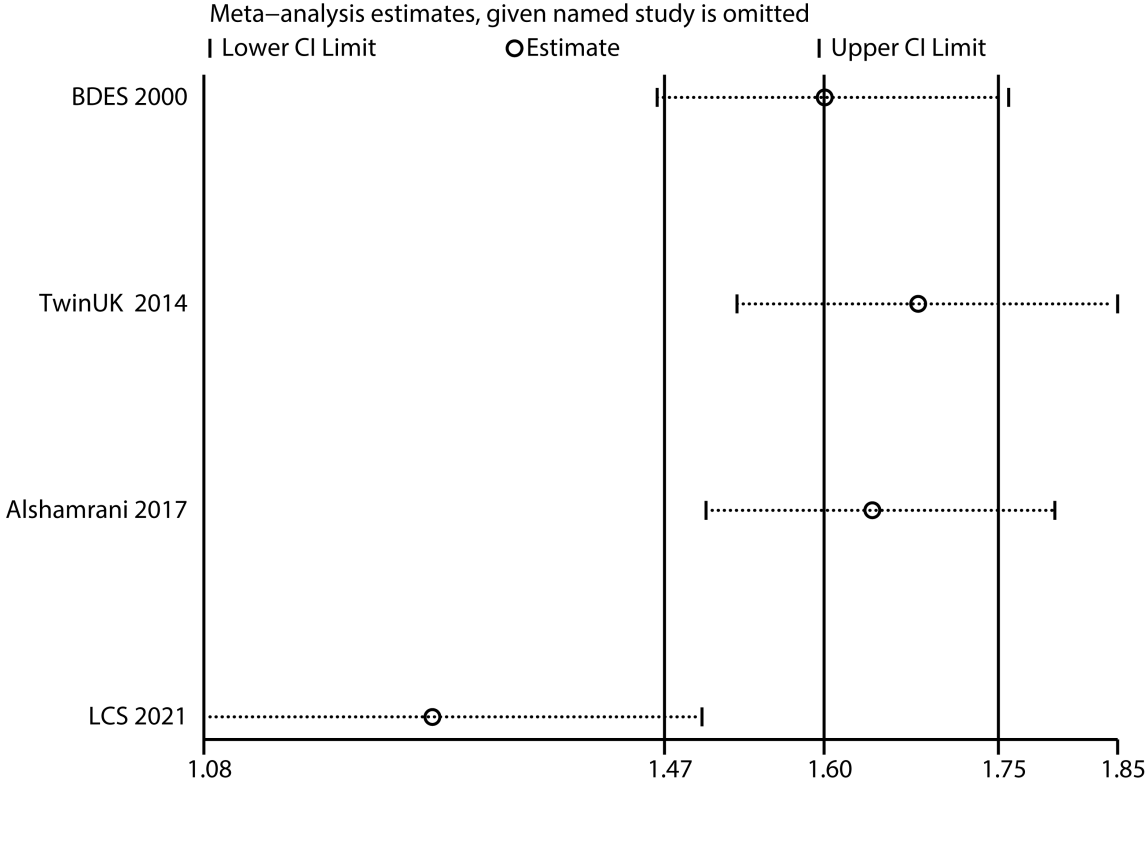


Figure S32. Sensitivity analysis for association of osteoporosis with the risk of dry eye syndrome


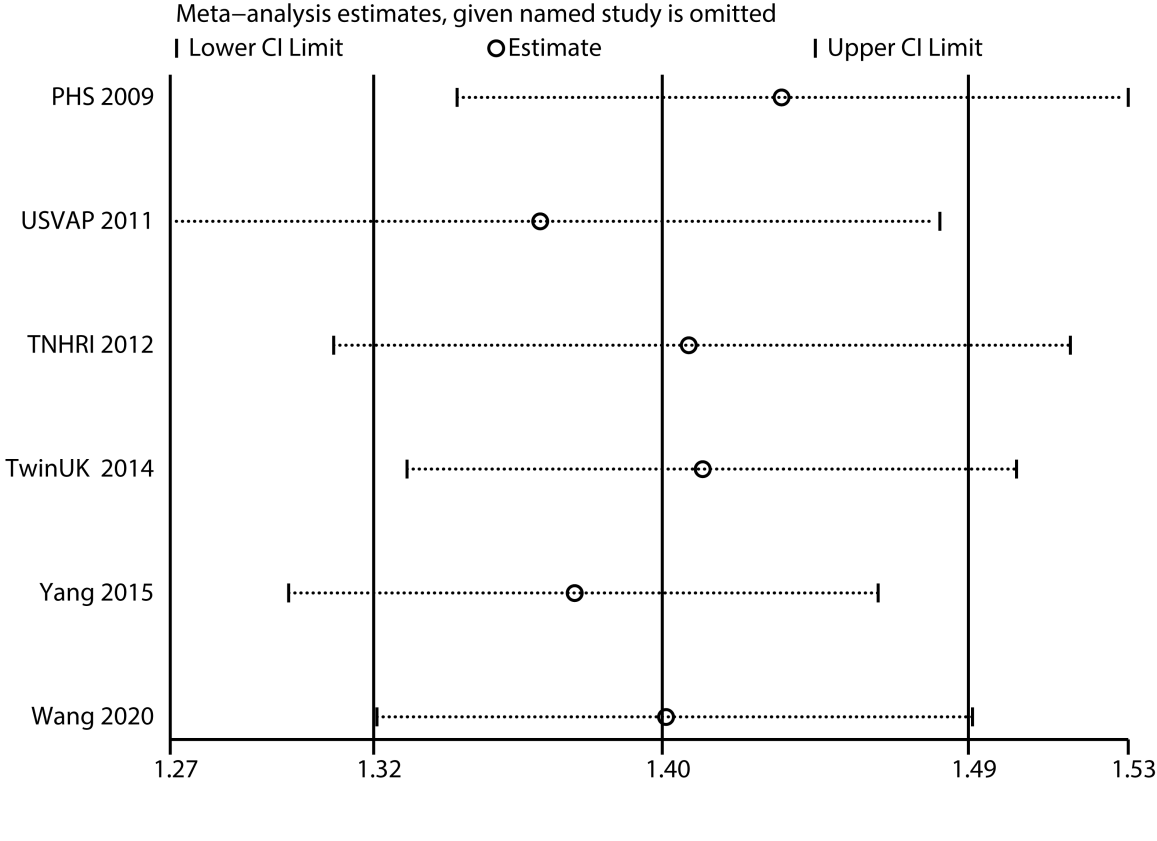


Figure S33. Sensitivity analysis for association of tumor with the risk of dry eye syndrome
