# Supplementary material for: Identified risk factors for dry eye syndrome: A systematic review and meta-analysis
Source: PLoS One. 2022 Aug 19;17(8):e0271267. doi: 10.1371/journal.pone.0271267 (PMC9390932; doi:10.1371/journal.pone.0271267)
Supplement: S4 File — (DOCX) [file pone.0271267.s006.docx]

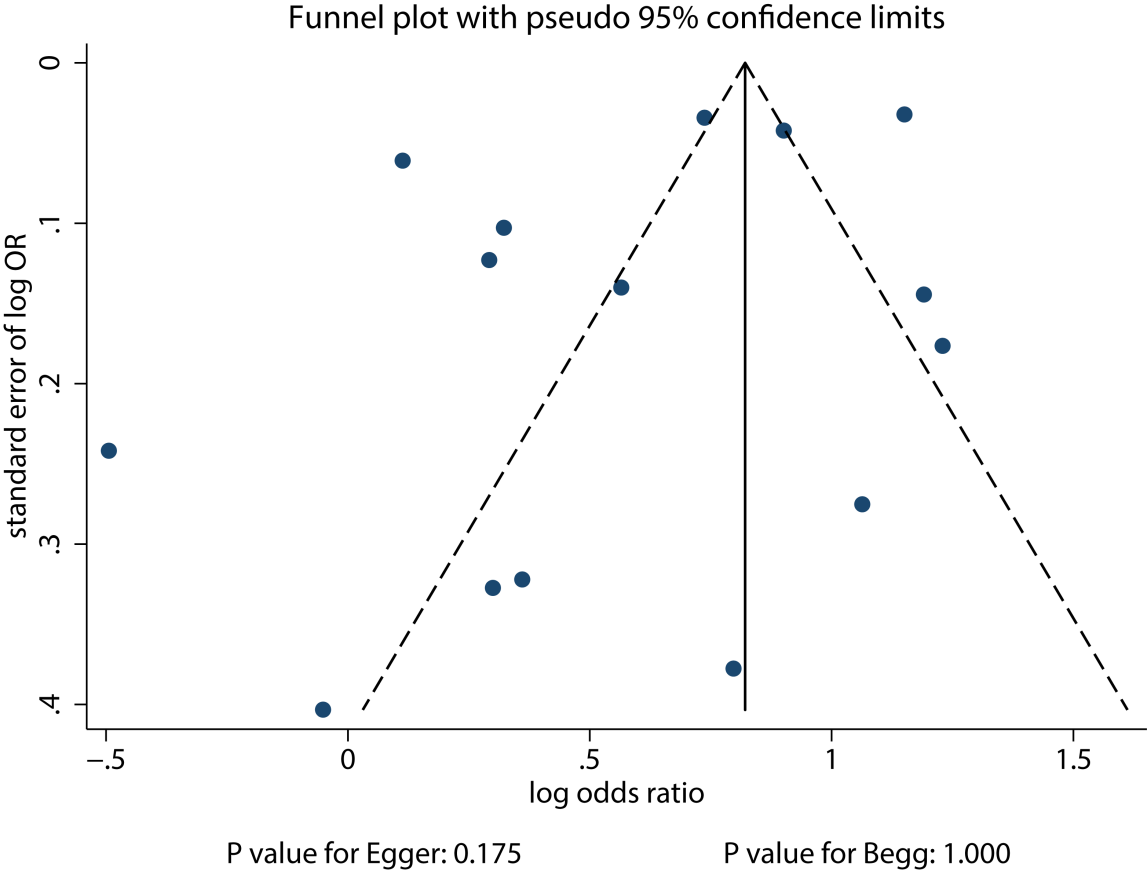


Figure S1. Funnel plot for elderly versus younger on subsequent dry eye syndrome risk


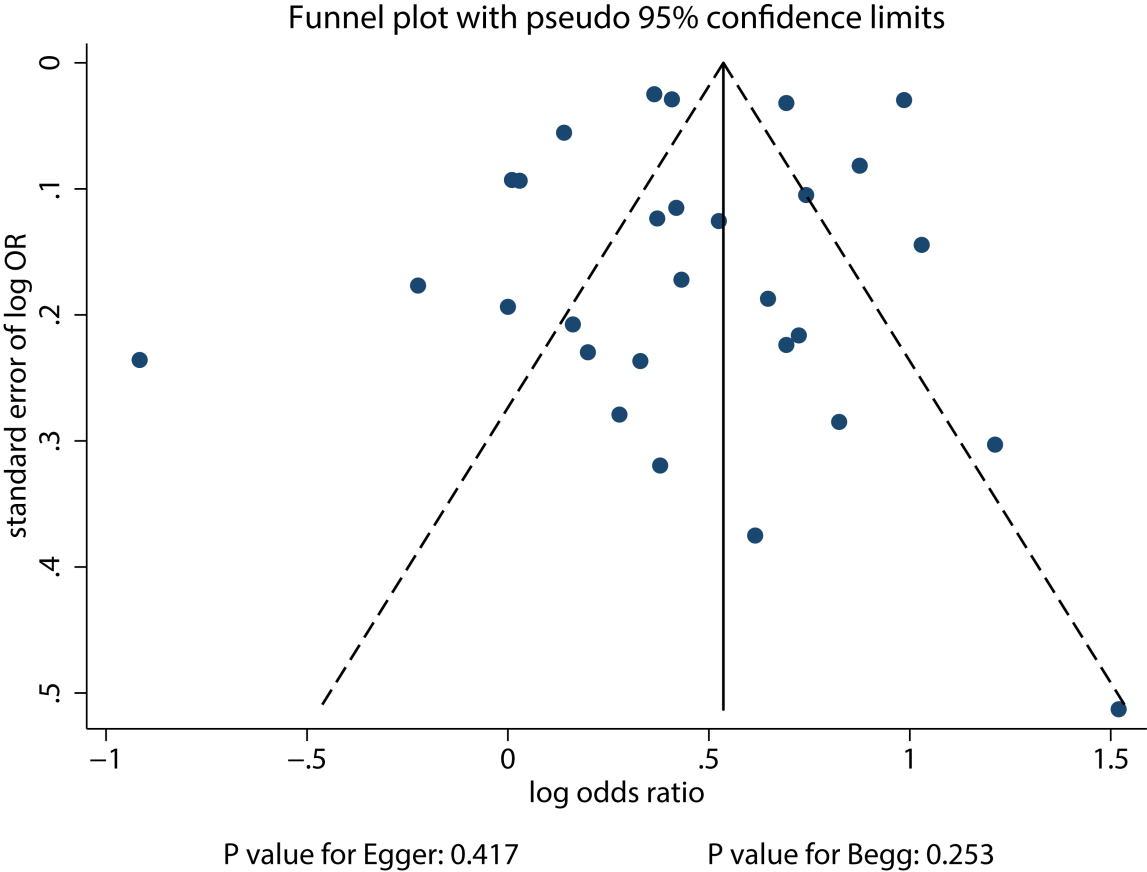


Figure S2. Funnel plot for female versus male on subsequent dry eye syndrome risk


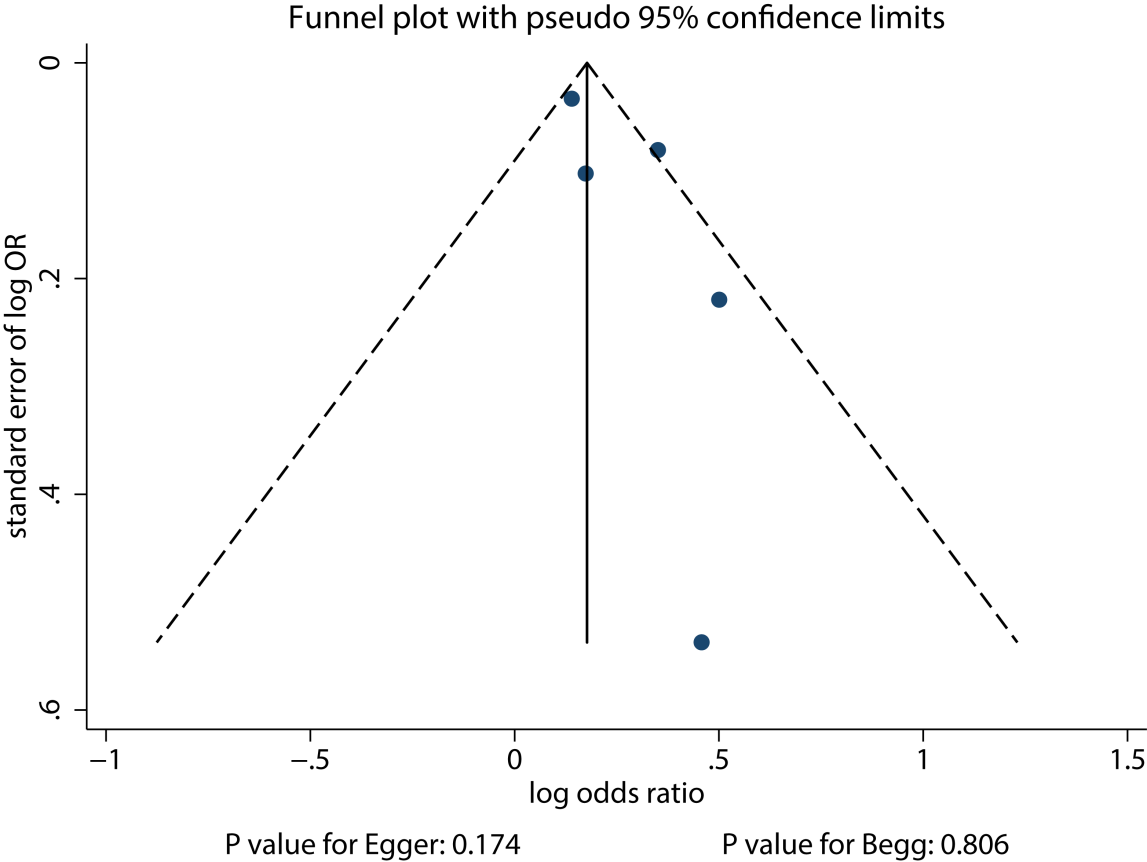


Figure S3. Funnel plot for other race versus white on subsequent dry eye syndrome risk


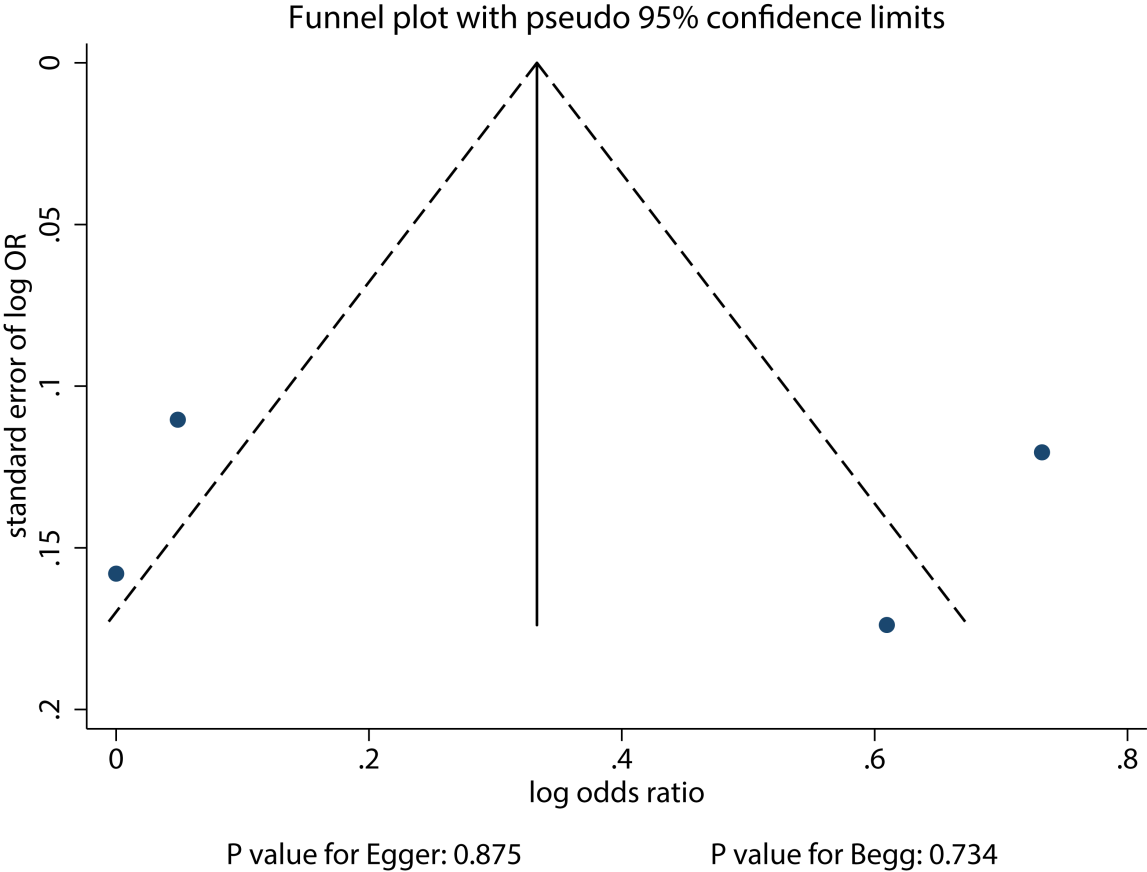


Figure S4. Funnel plot for urban versus rural residence on subsequent dry eye syndrome risk


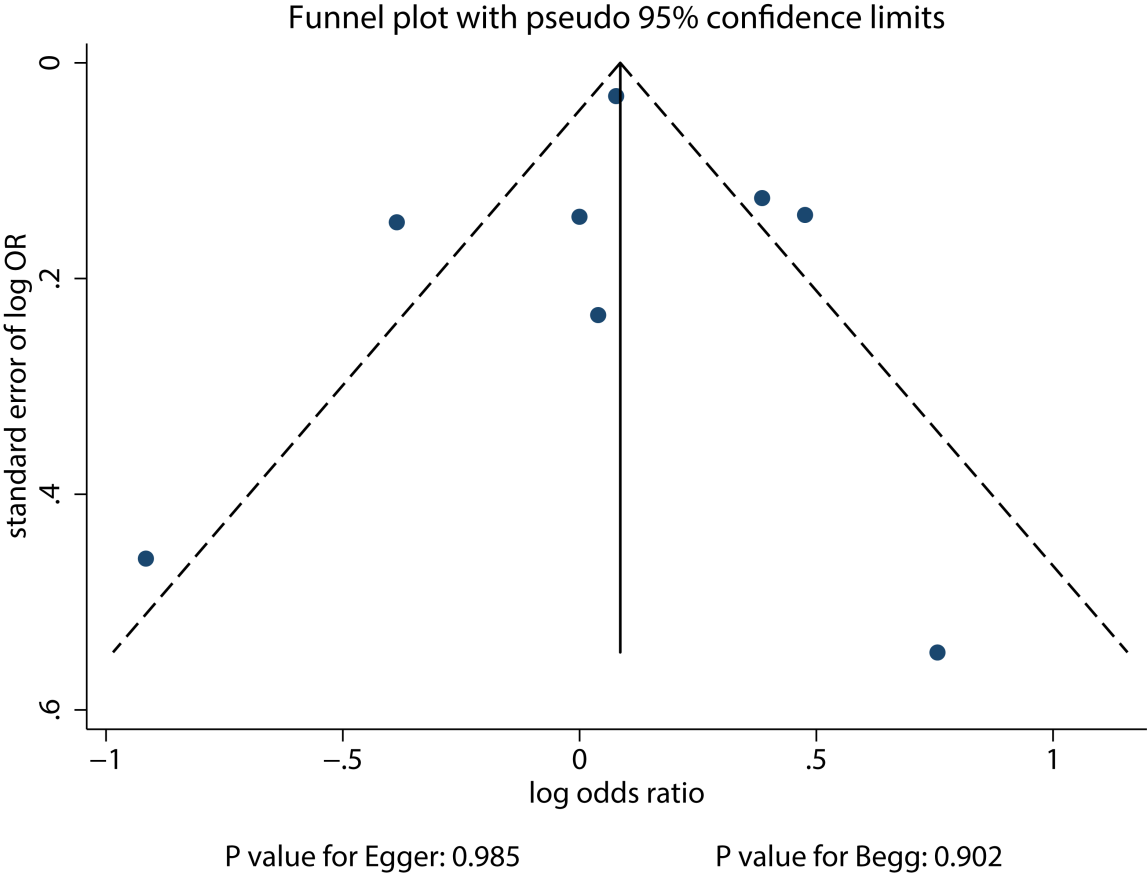


Figure S5. Funnel plot for high versus low education level on subsequent dry eye syndrome risk


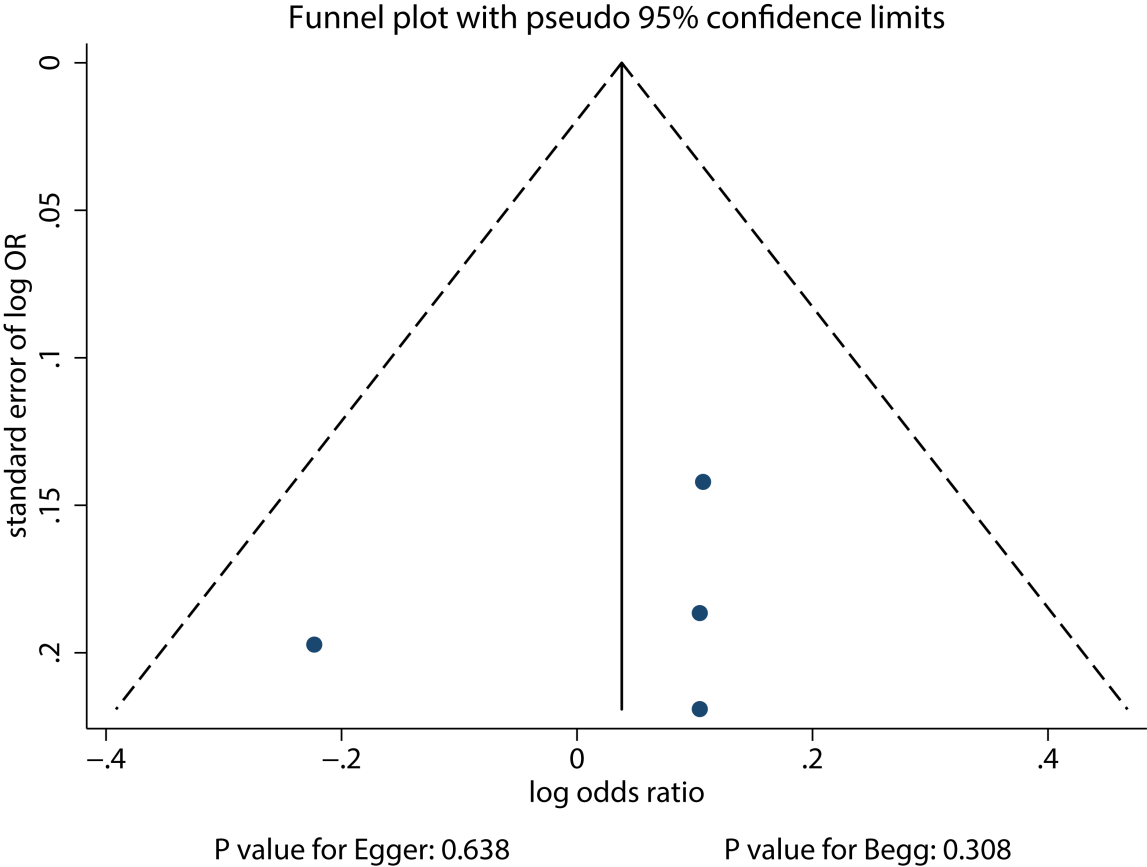


Figure S6. Funnel plot for association of obesity with the risk of dry eye syndrome


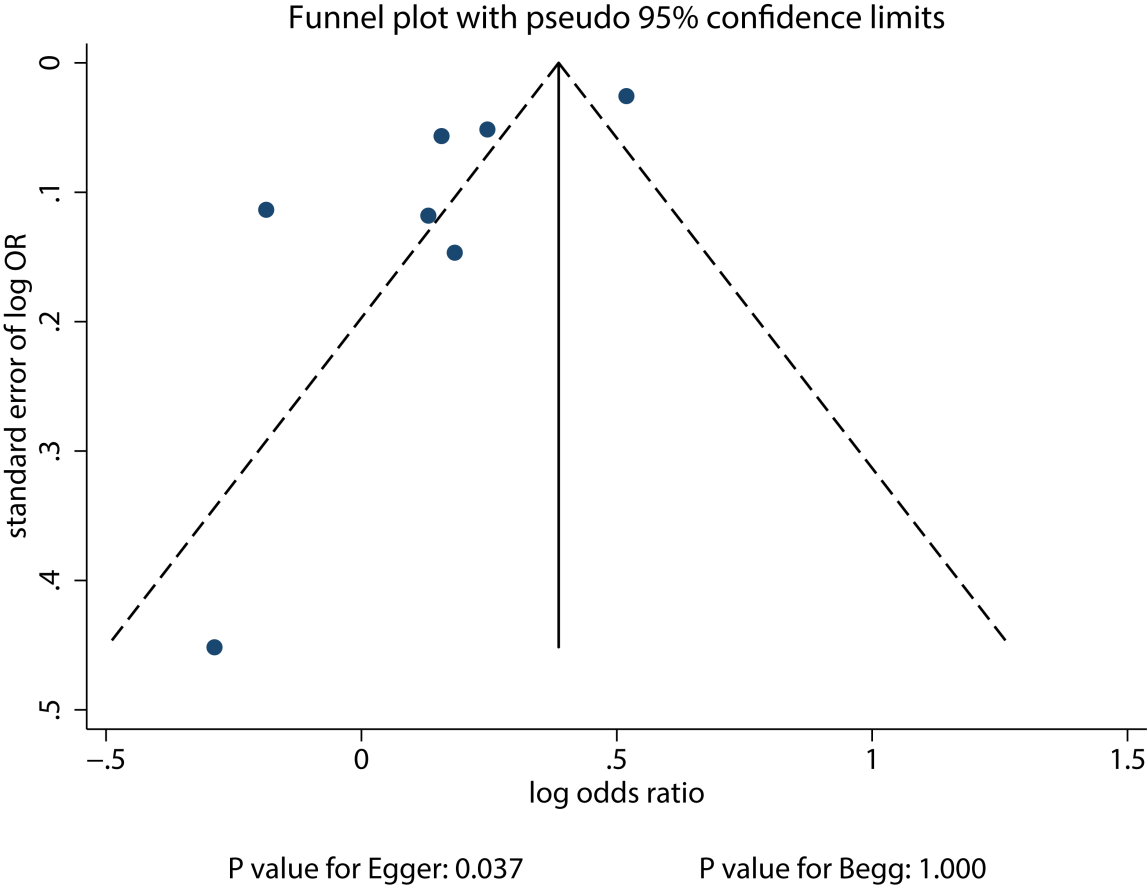


Figure S7. Funnel plot for association of dyslipidemia with the risk of dry eye syndrome


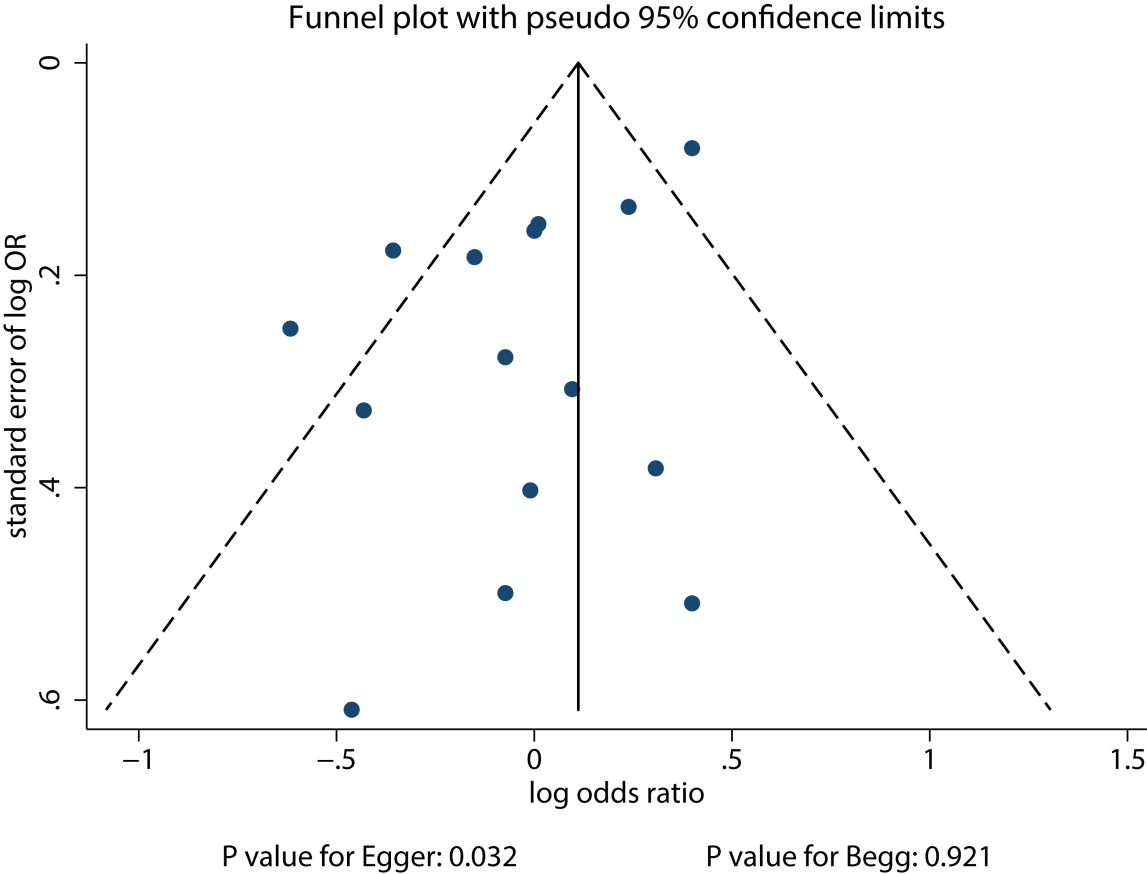


Figure S8. Funnel plot for alcohol intake on subsequent risk of dry eye syndrome


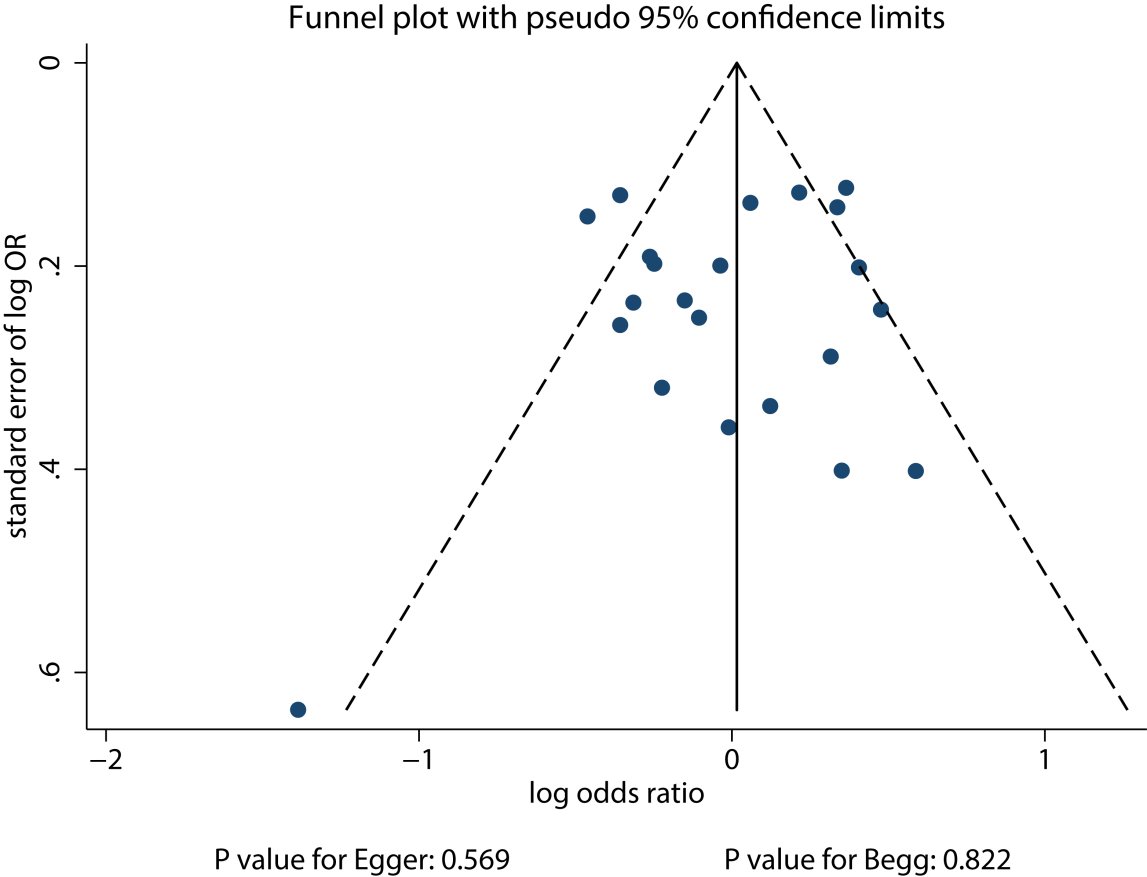


Figure S9. Funnel plot for association of smoking with the risk of dry eye syndrome


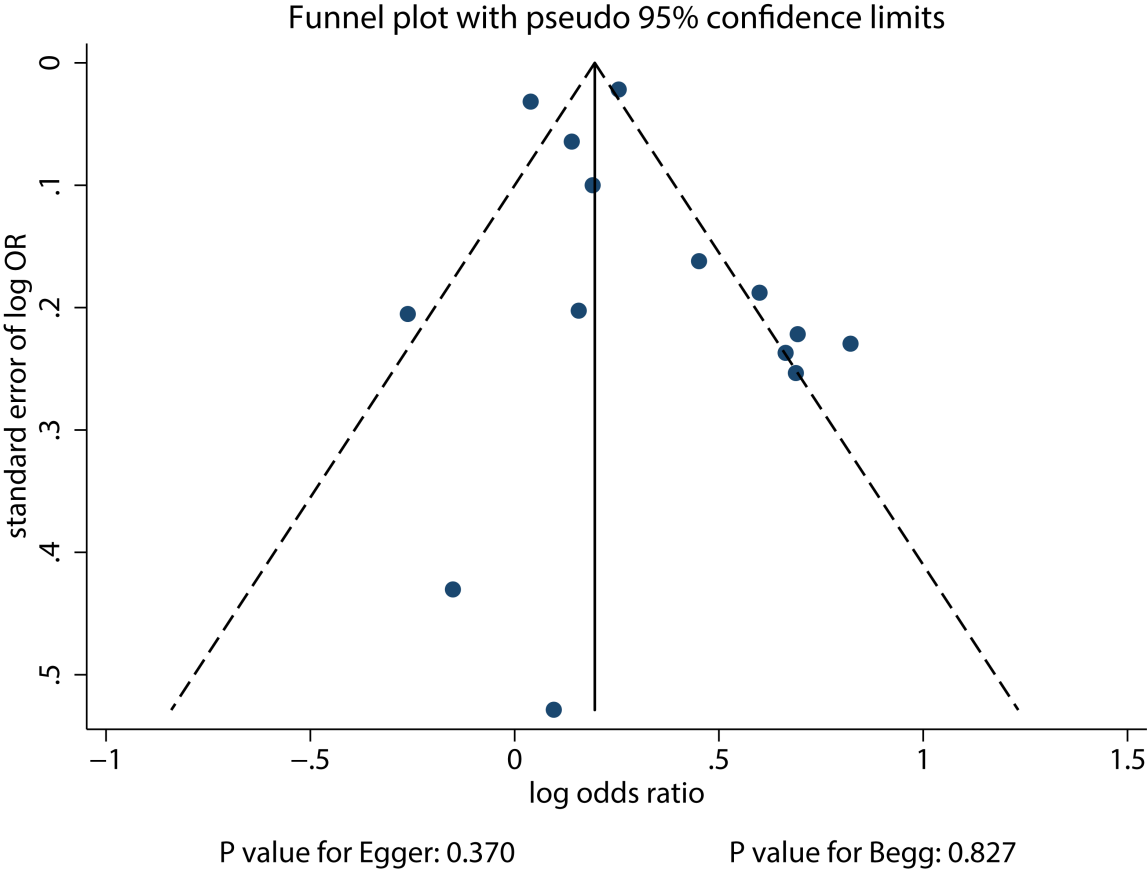


Figure S10. Funnel plot for association of VDT use with the risk of dry eye syndrome


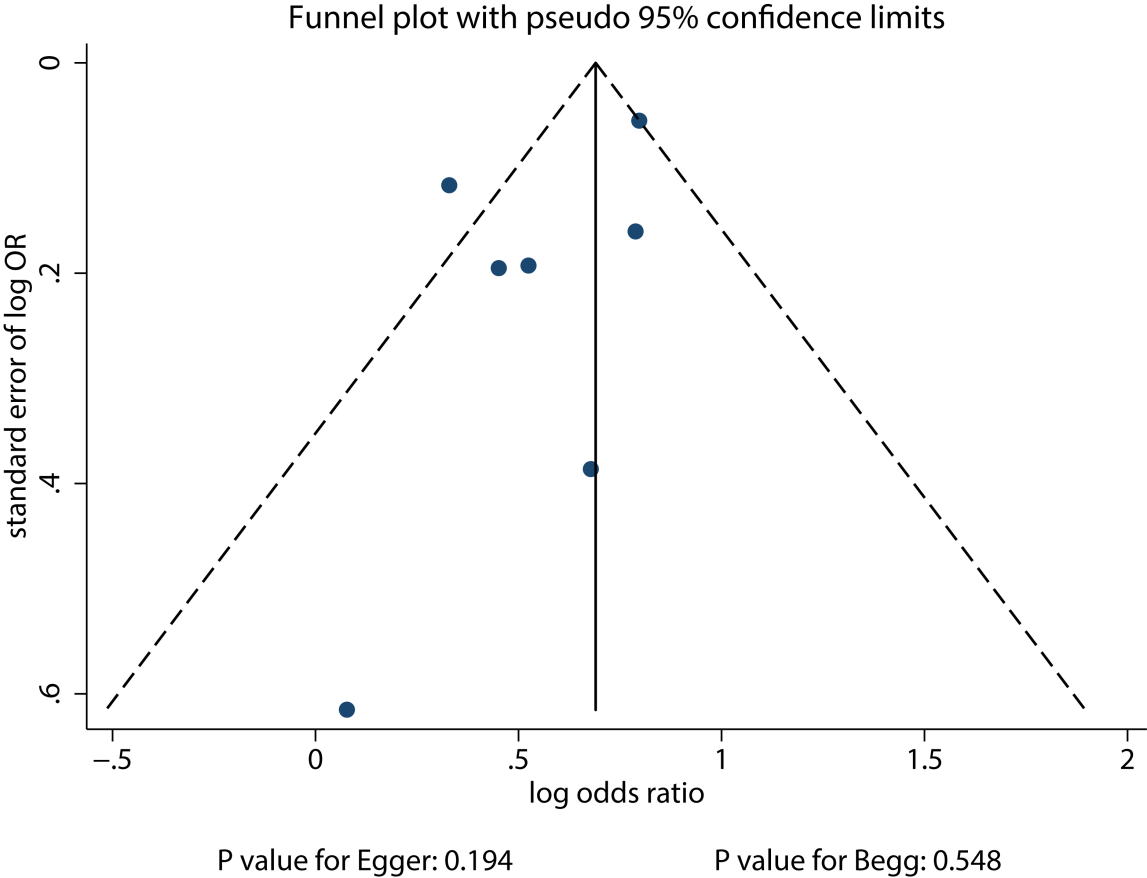


Figure S11. Funnel plot for association of cataract surgery with the risk of dry eye syndrome


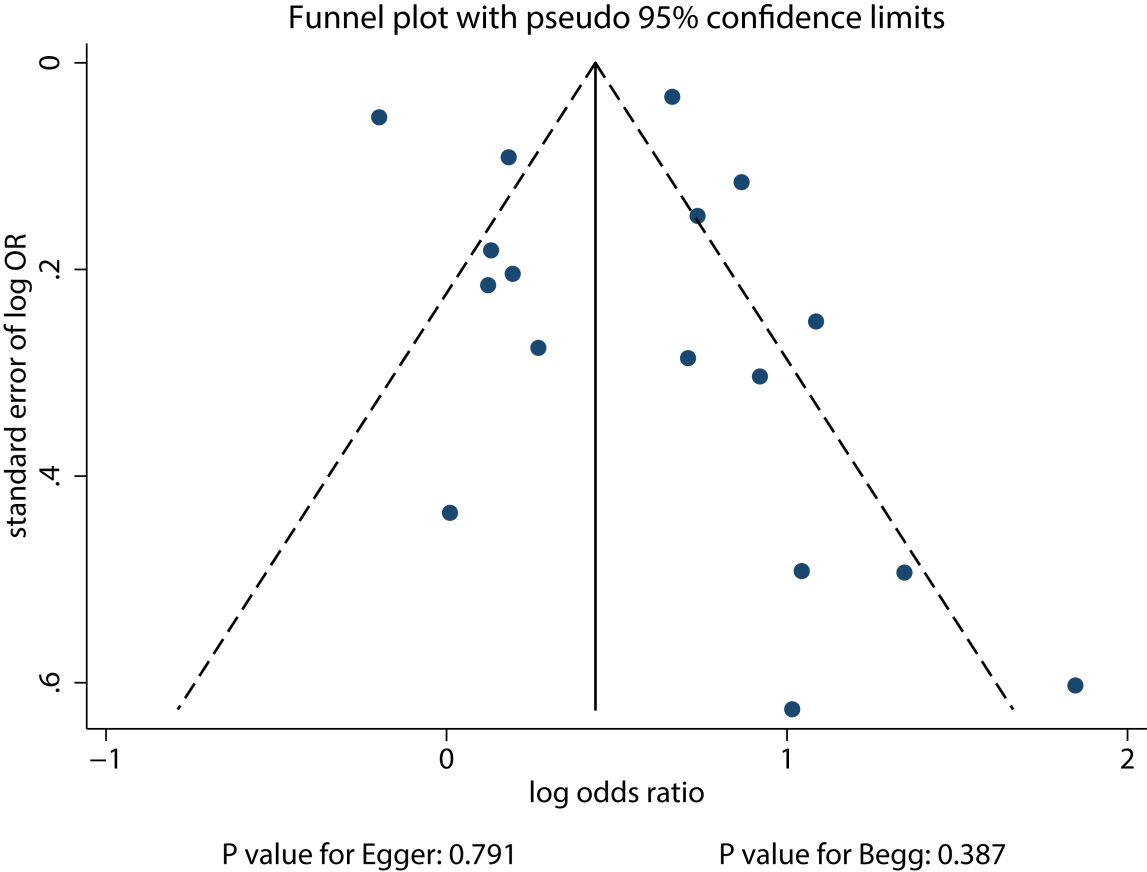


Figure S12. Funnel plot for association of contact lens wear with the risk of dry eye syndrome


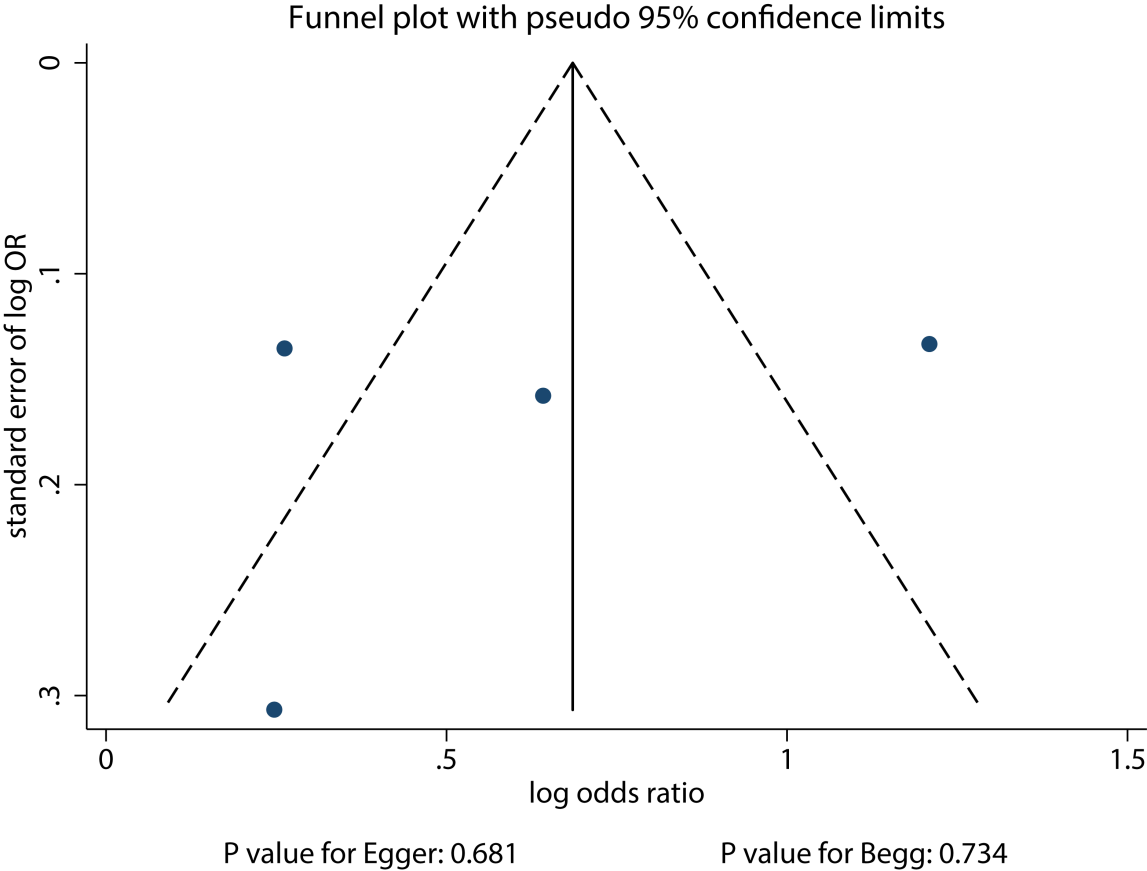


Figure S13. Funnel plot for association of pterygium with the risk of dry eye syndrome


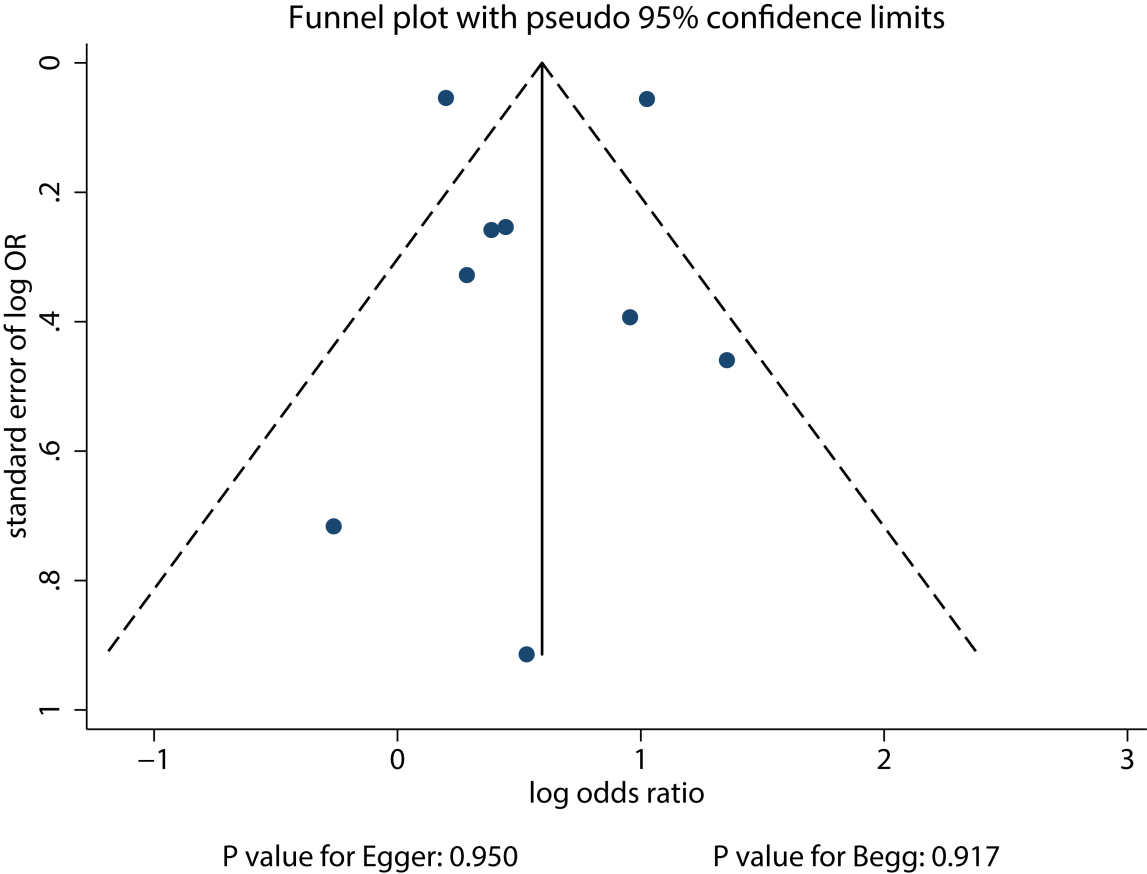


Figure S14. Funnel plot for association of glaucoma with the risk of dry eye syndrome


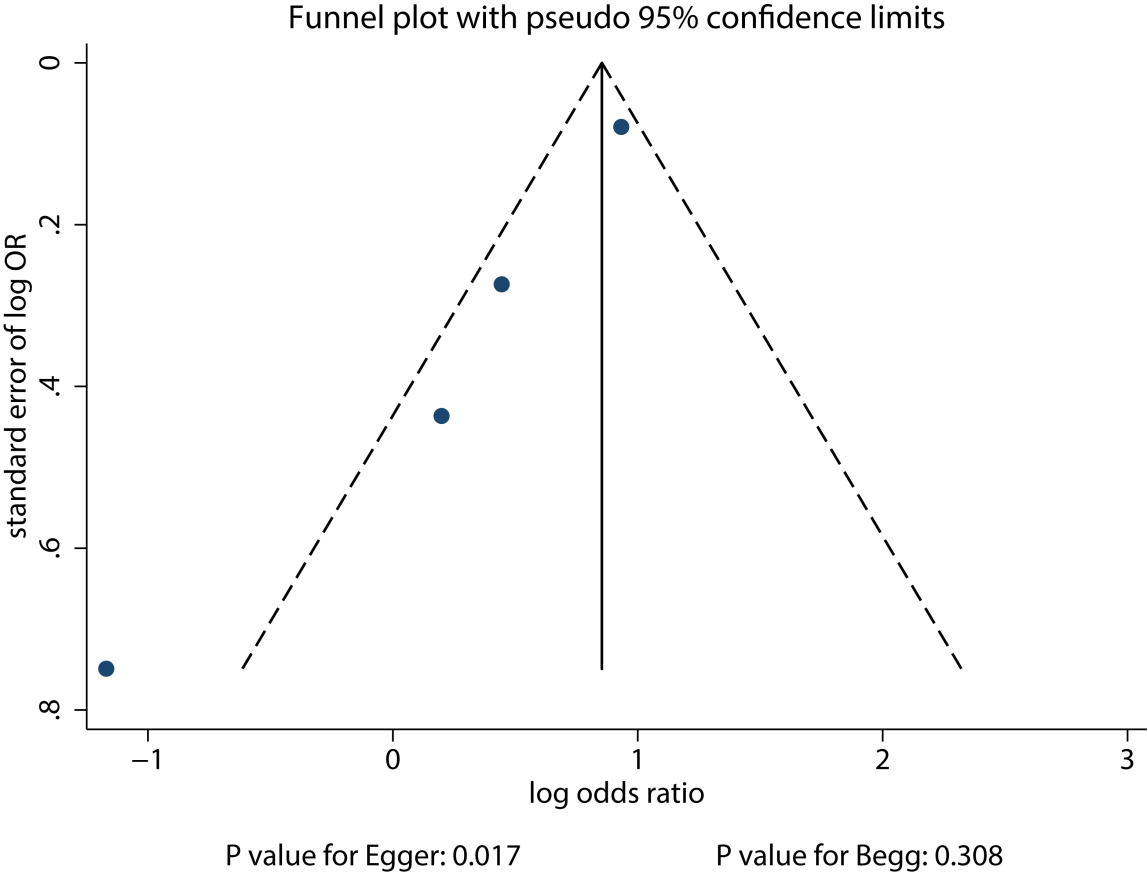


Figure S15. Funnel plot for association of age-related maculopathy with the risk of dry eye syndrome


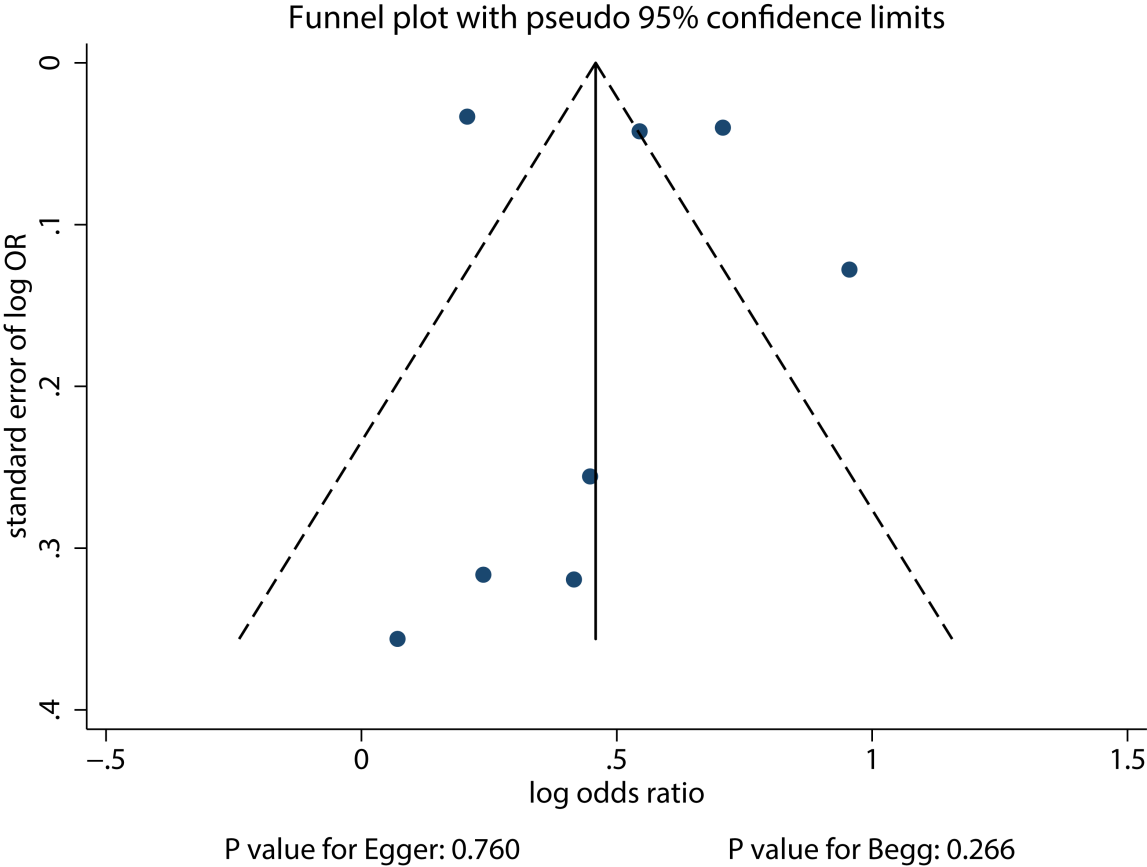


Figure S16. Funnel plot for association of eye surgery with the risk of dry eye syndrome


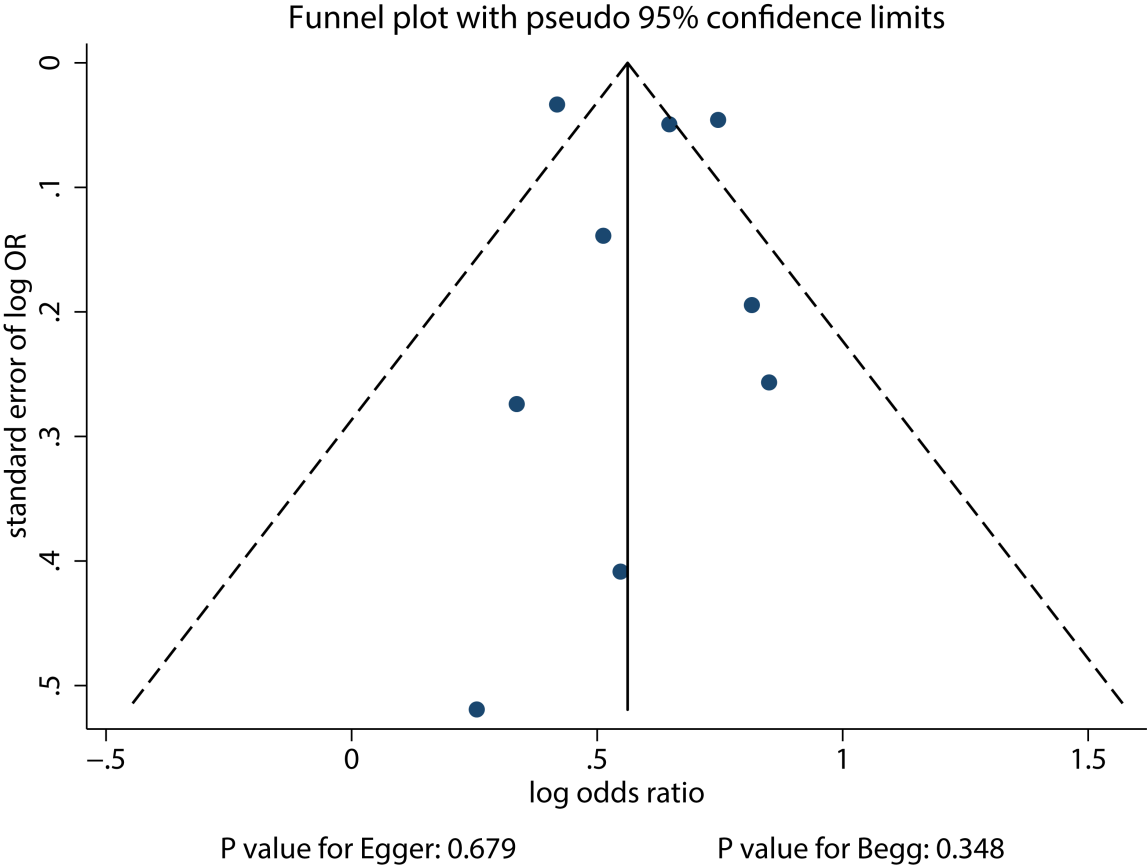


Figure S17. Funnel plot for association of depression with the risk of dry eye syndrome


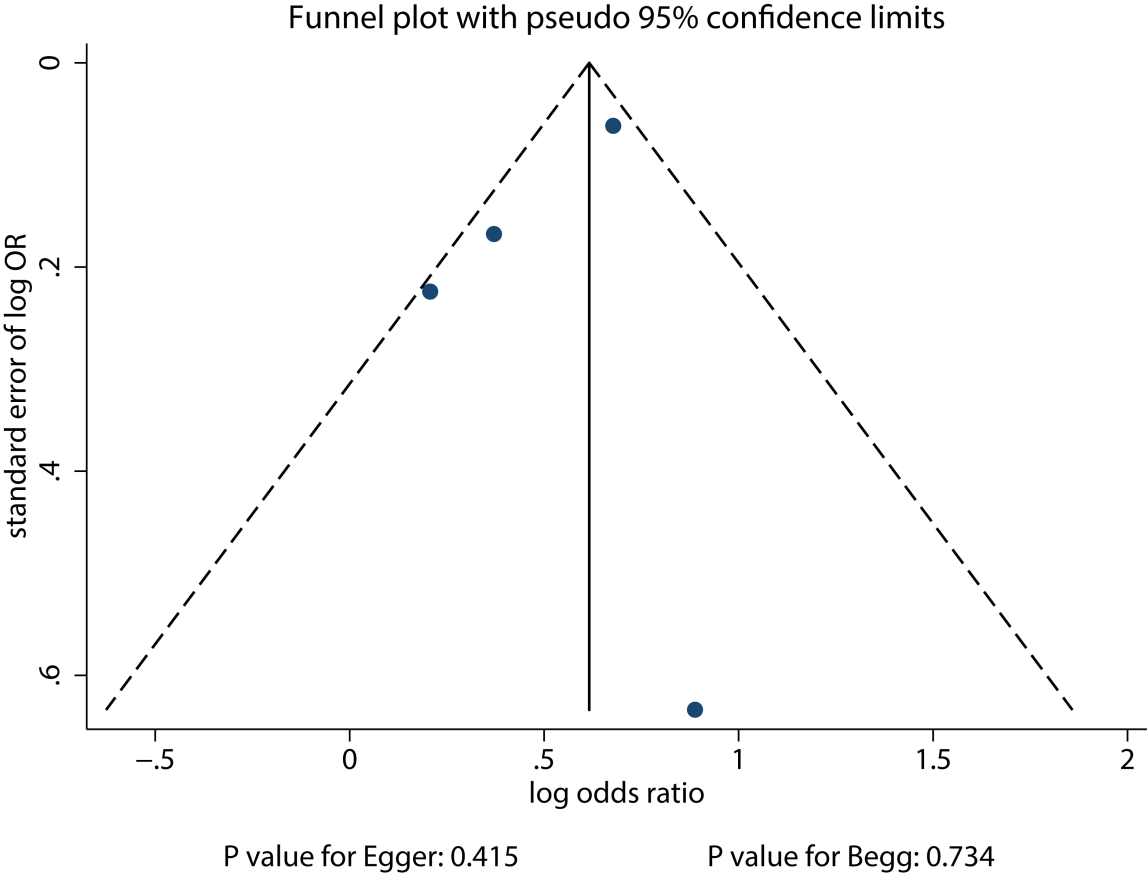


Figure S18. Funnel plot for association of PTSD with the risk of dry eye syndrome


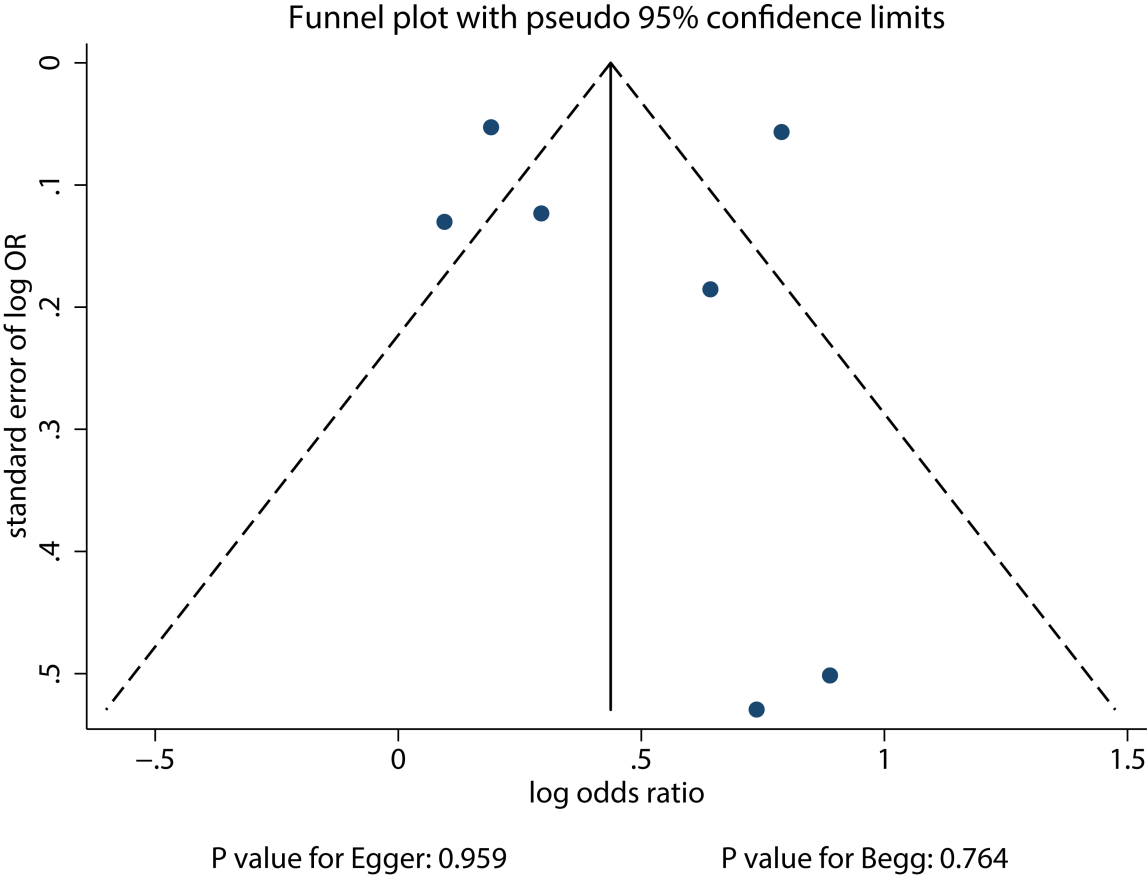


Figure S19. Funnel plot for association of sleep apnea with the risk of dry eye syndrome


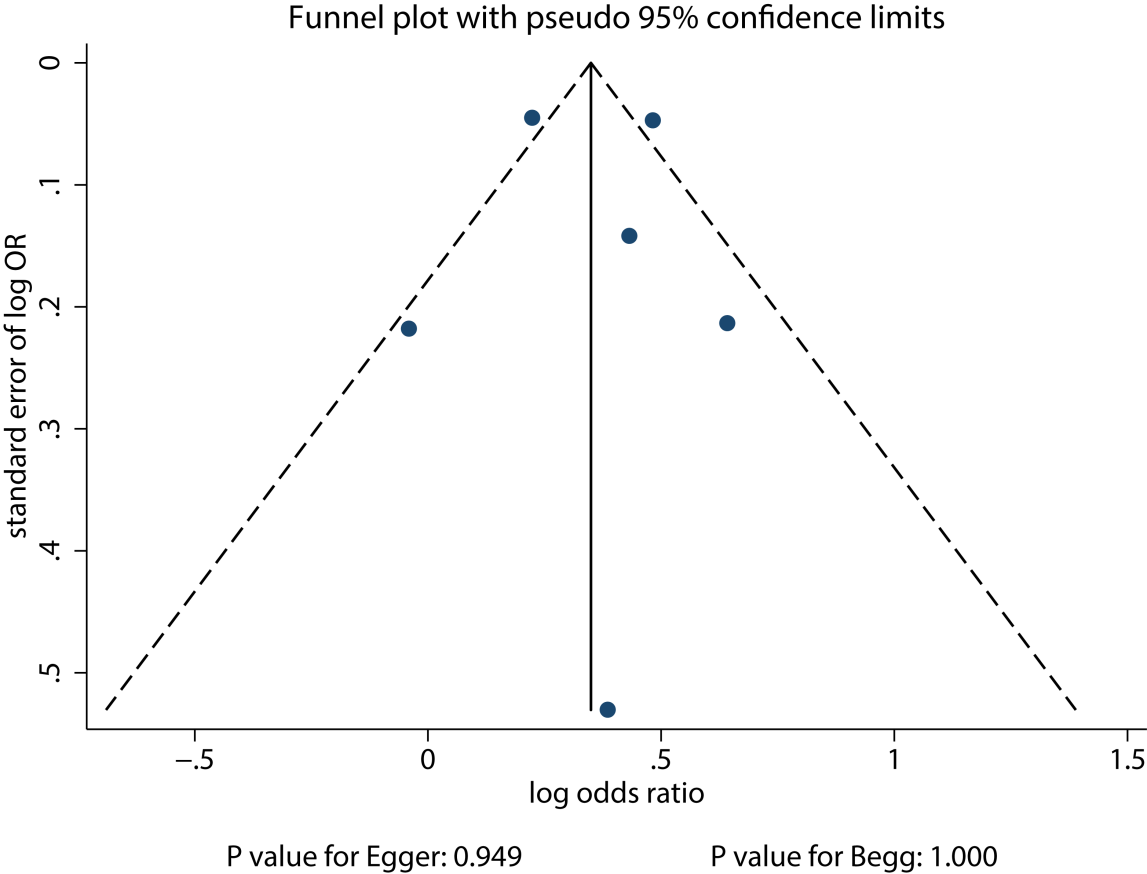


Figure S20. Funnel plot for association of asthma with the risk of dry eye syndrome


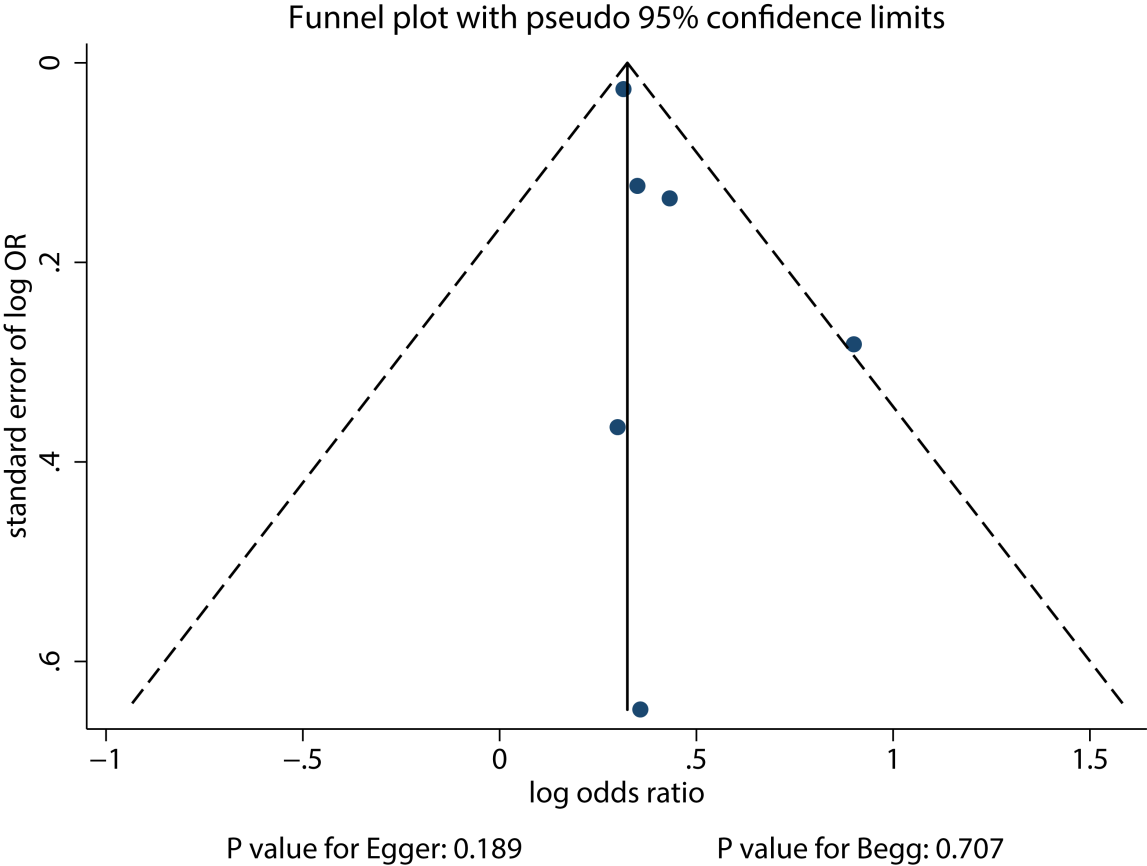


Figure S21. Funnel plot for association of allergy with the risk of dry eye syndrome


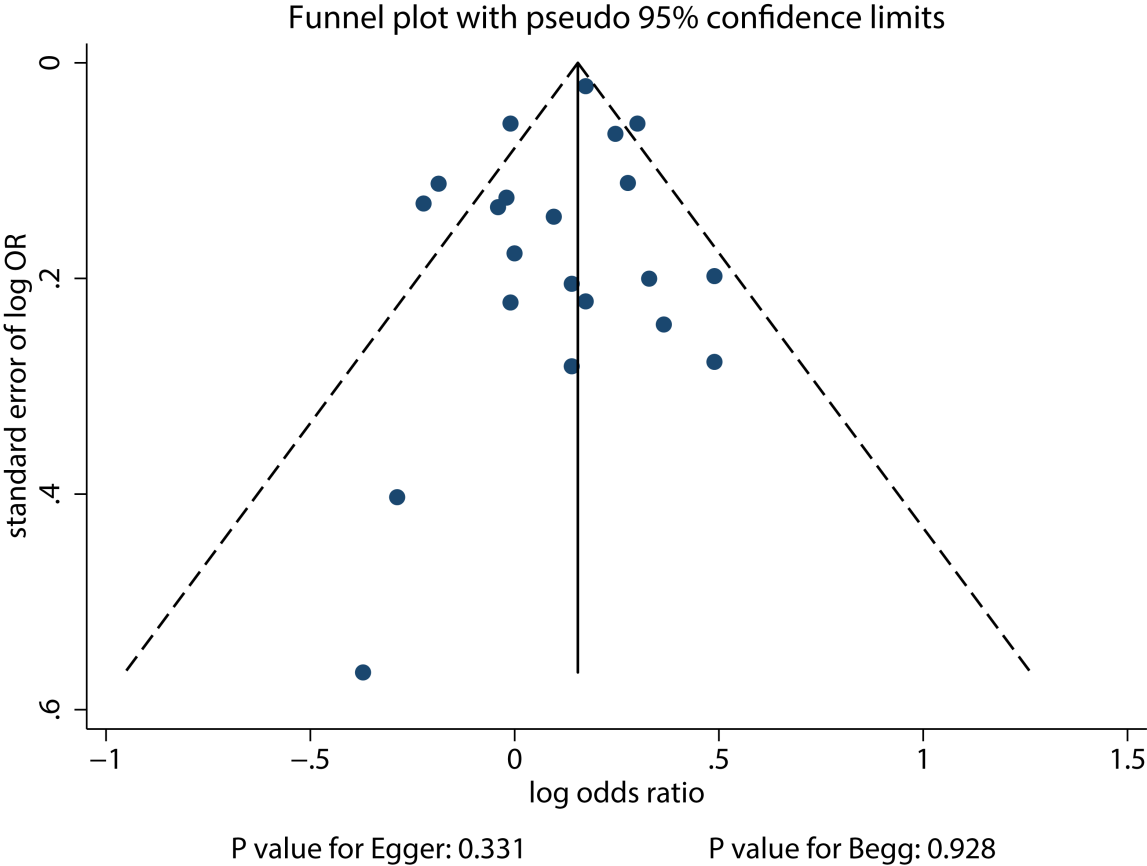


Figure S22. Funnel plot for association of hypertension with the risk of dry eye syndrome


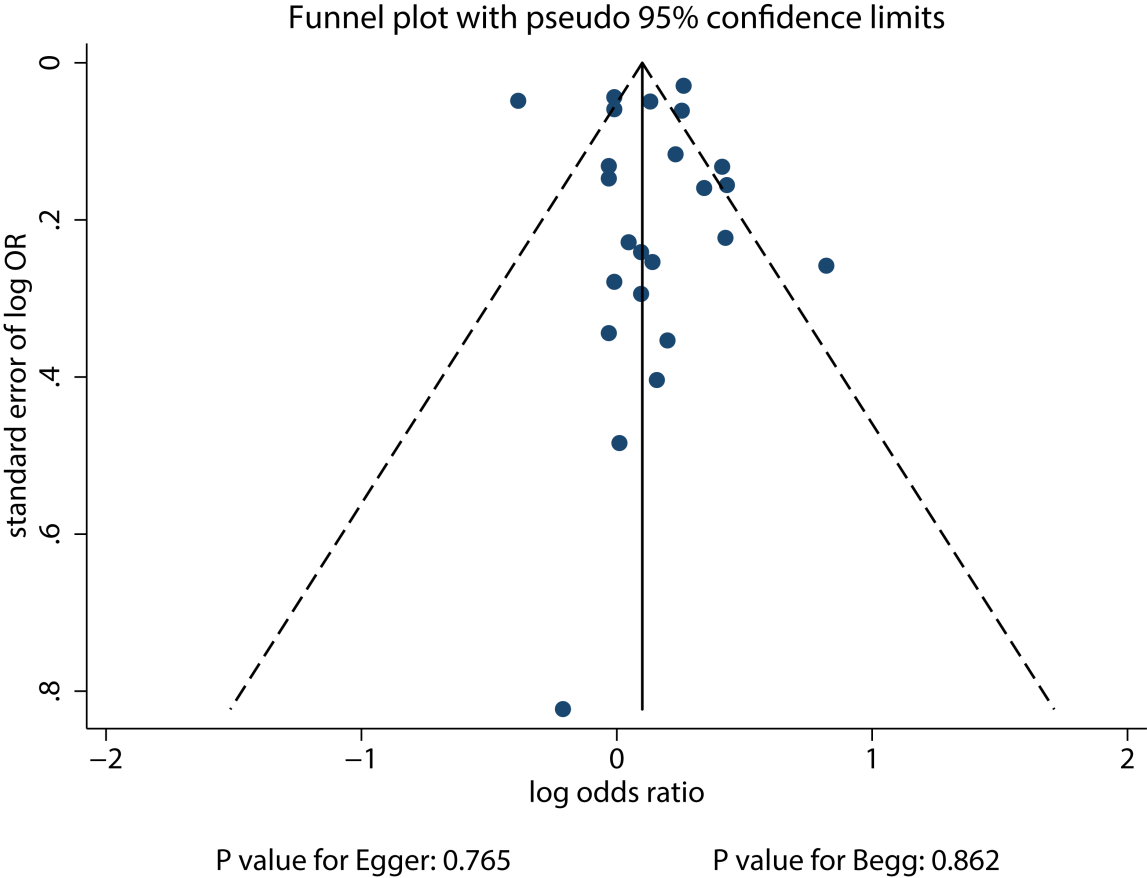


Figure S23. Funnel plot for association of DM with the risk of dry eye syndrome


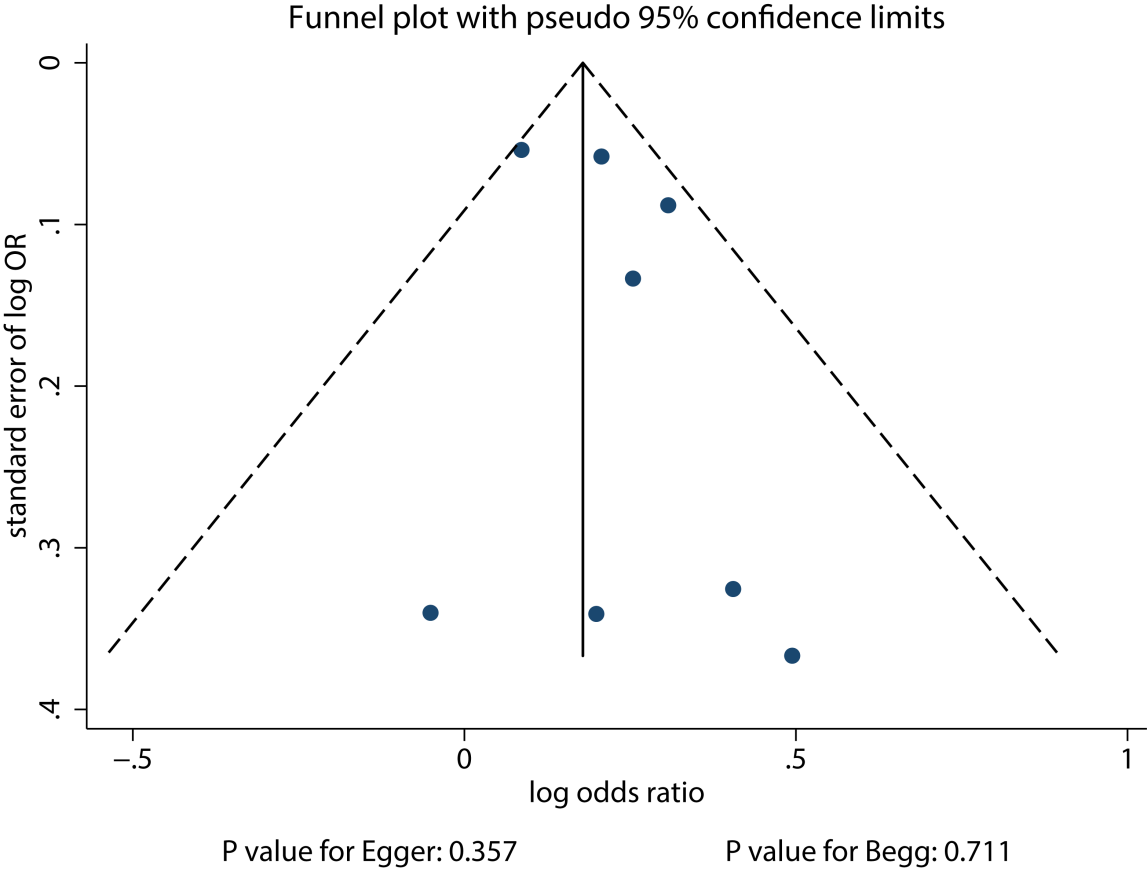


Figure S24. Funnel plot for association of CVD with the risk of dry eye syndrome


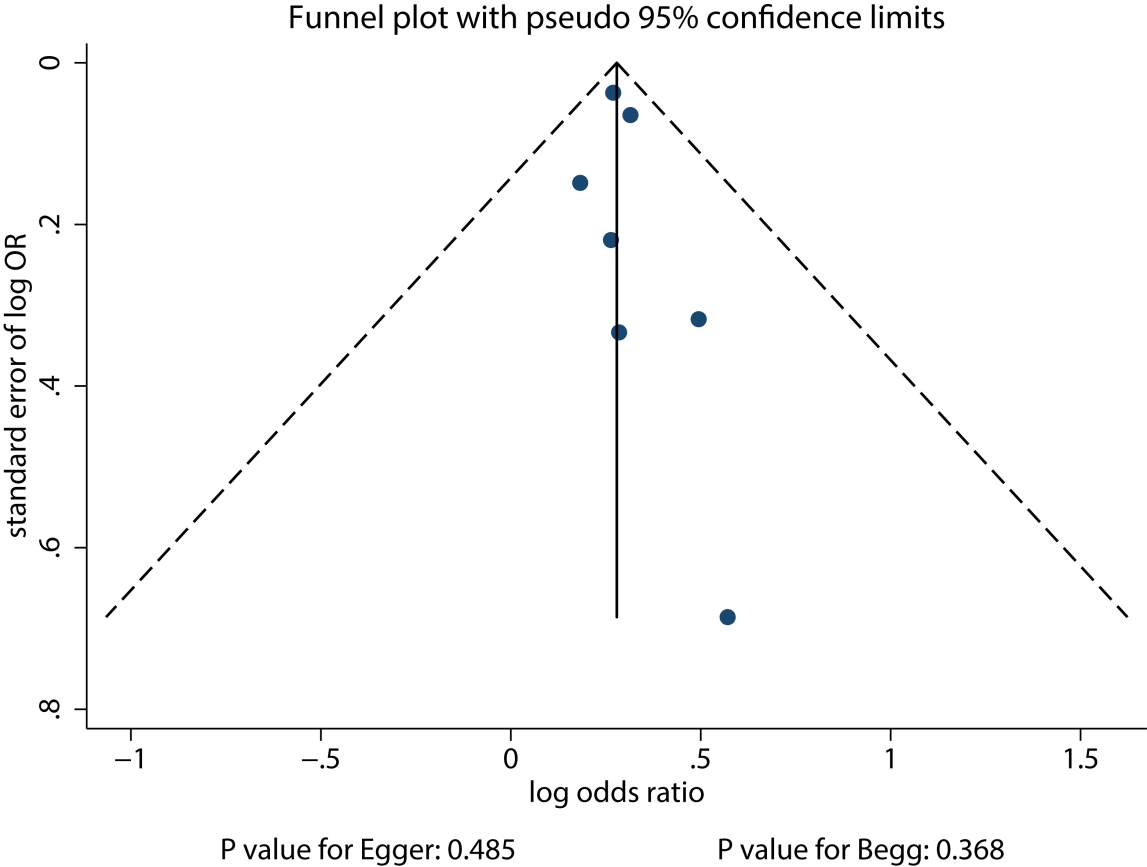


Figure S25. Funnel plot for association of stroke with the risk of dry eye syndrome


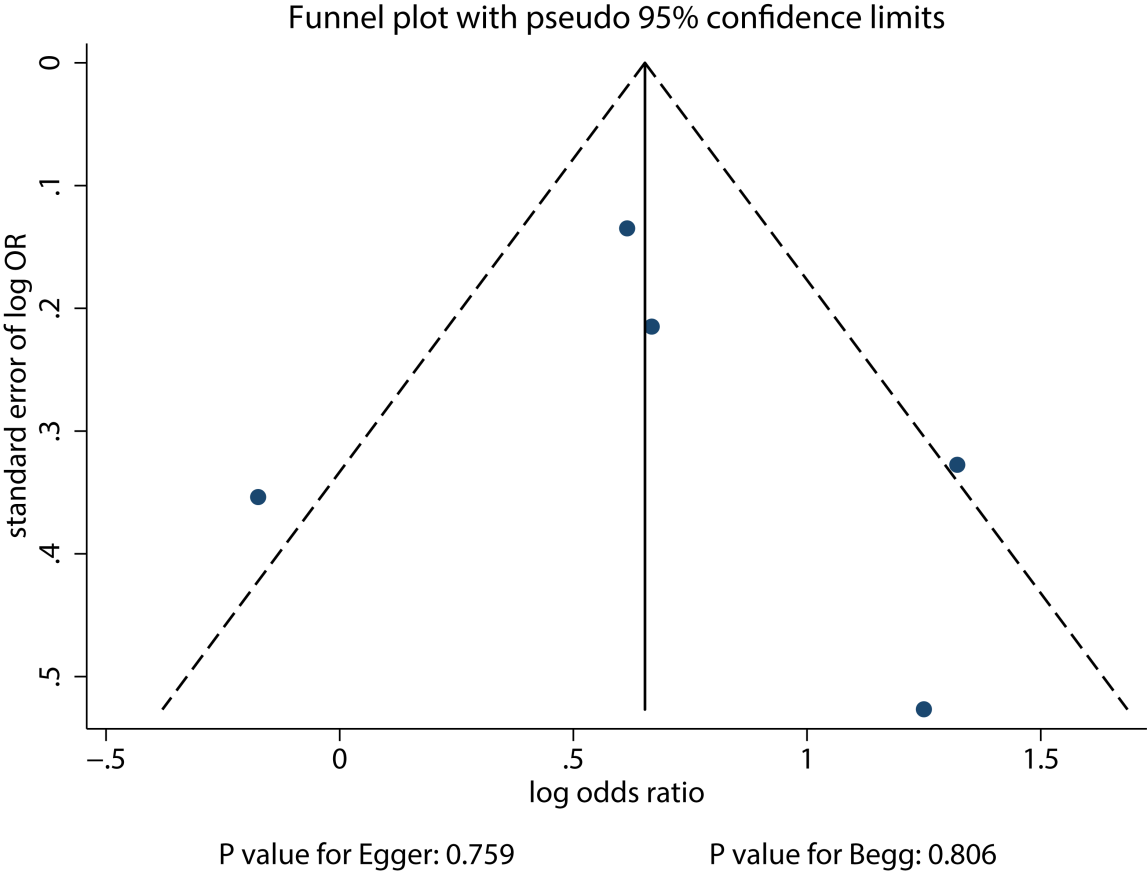


Figure S26. Funnel plot for association of rosacea with the risk of dry eye syndrome


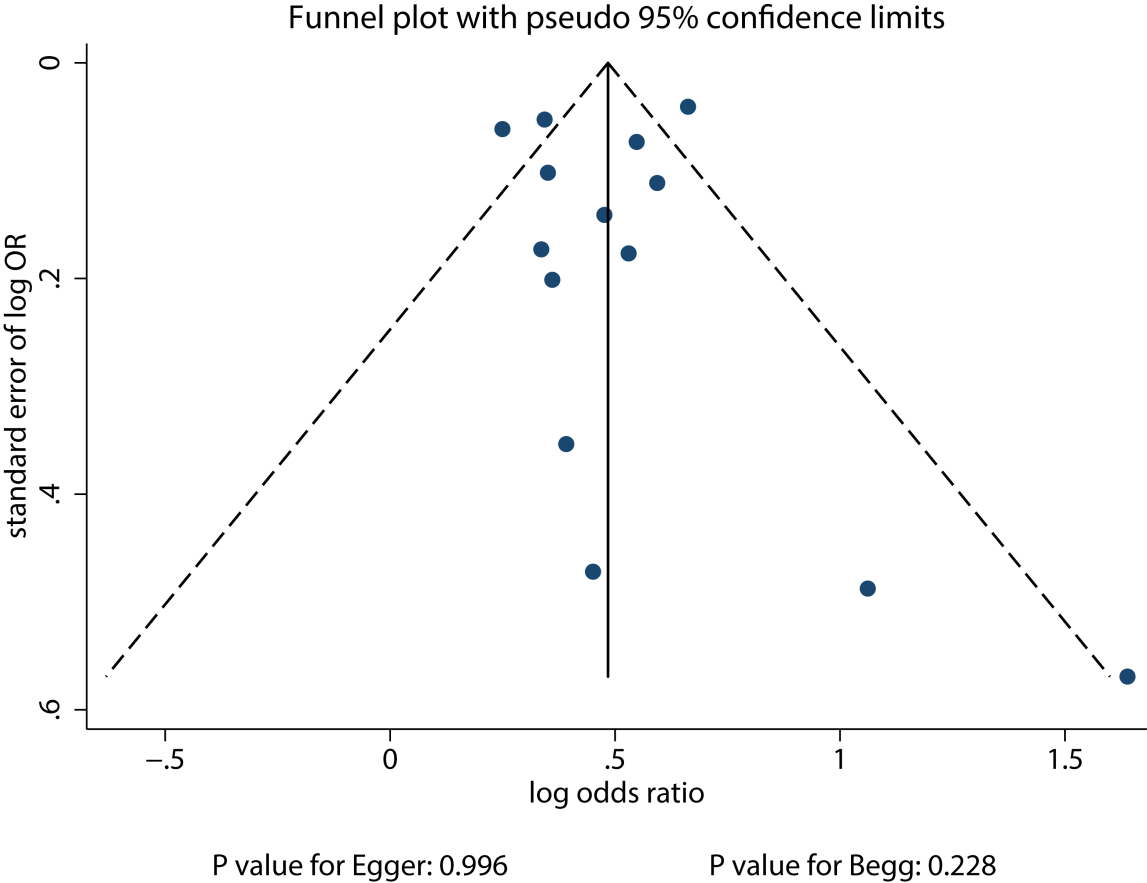


Figure S27. Funnel plot for association of thyroid disease with the risk of dry eye syndrome


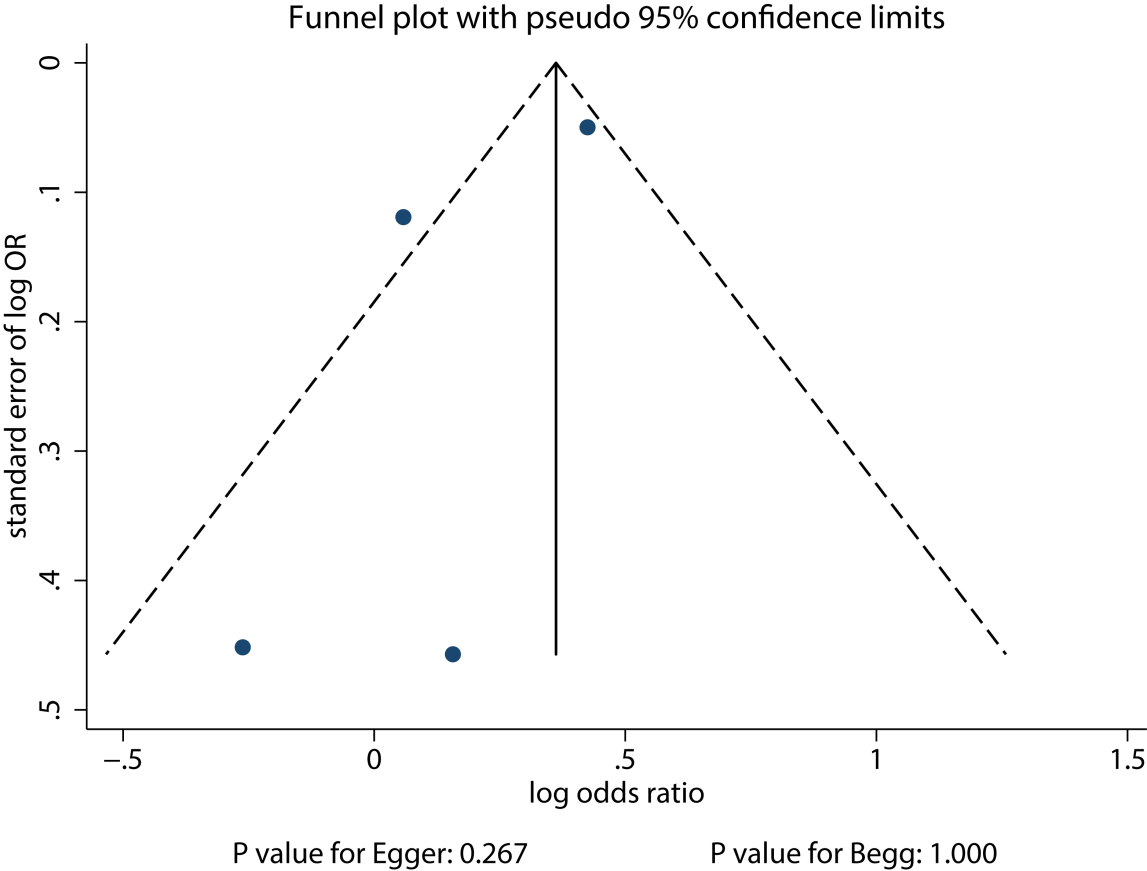


Figure S28. Funnel plot for association of COPD with the risk of dry eye syndrome


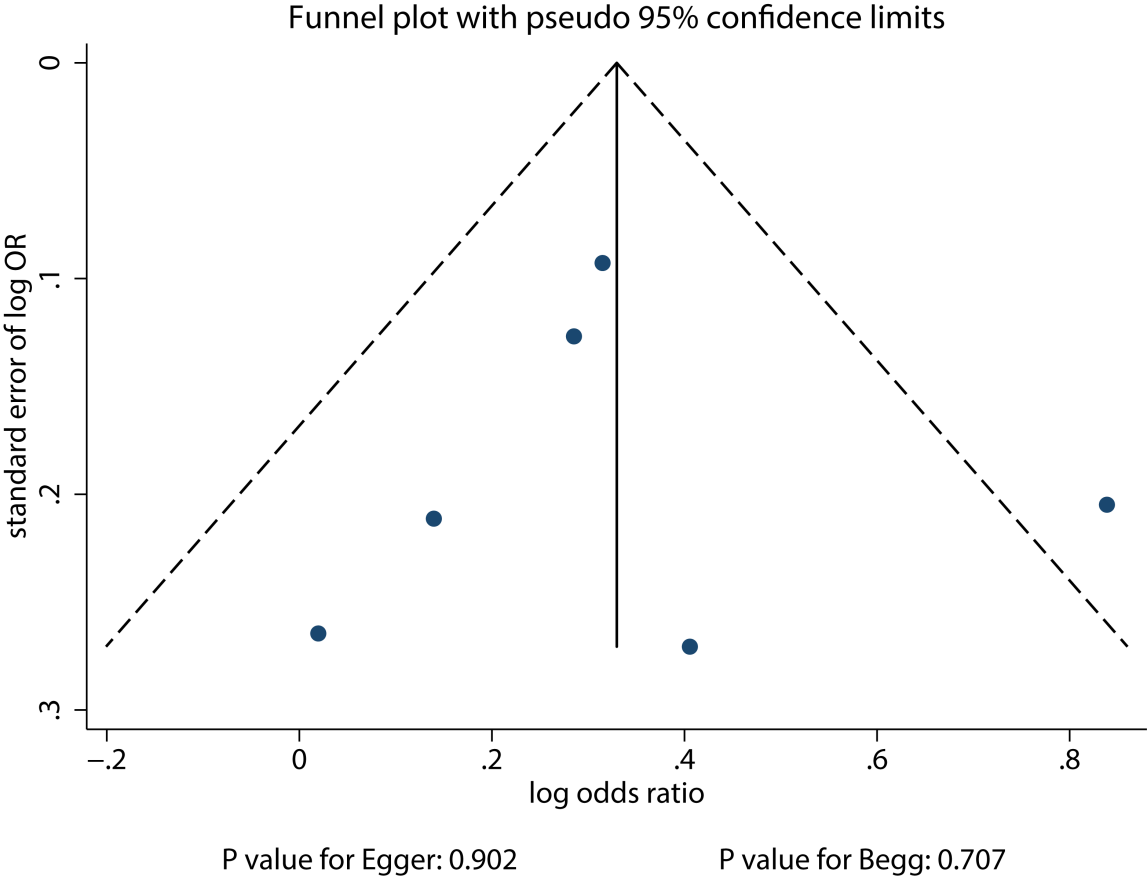


Figure S29. Funnel plot for association of gout with the risk of dry eye syndrome


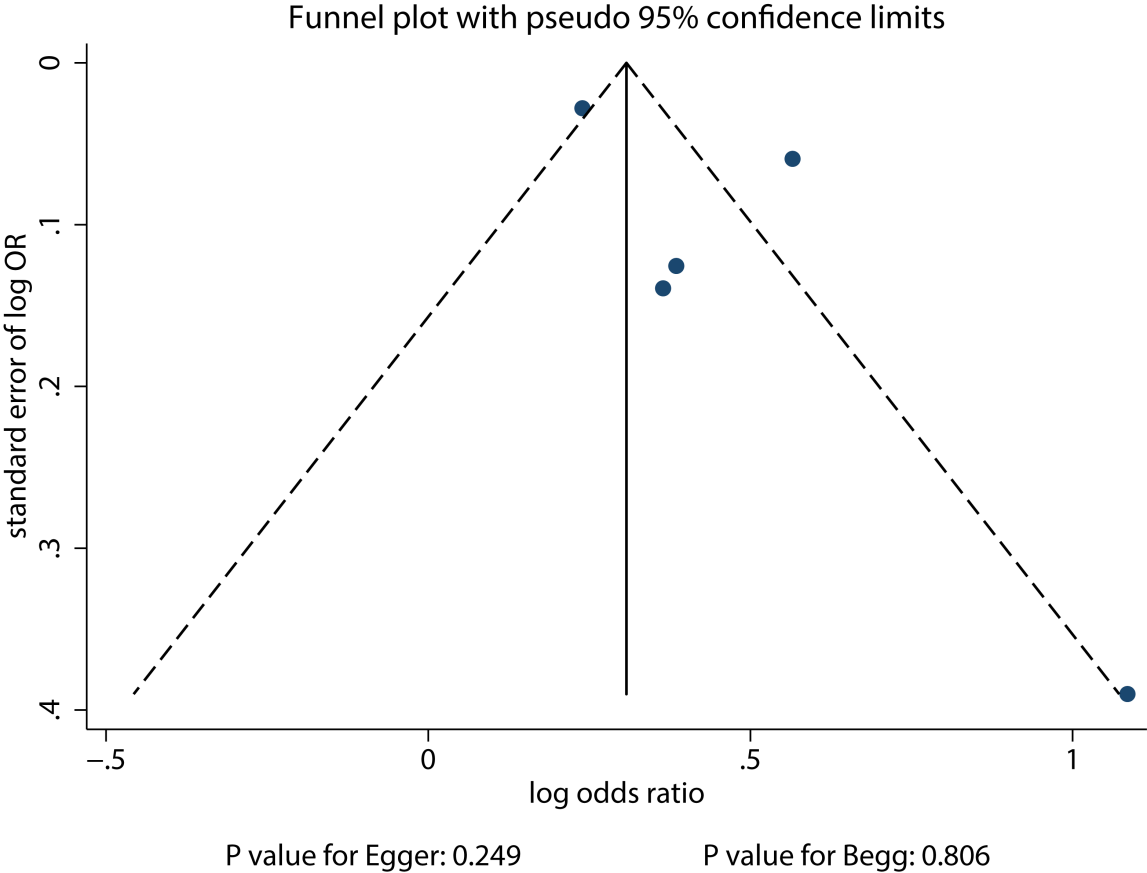


Figure S30. Funnel plot for association of migraines with the risk of dry eye syndrome


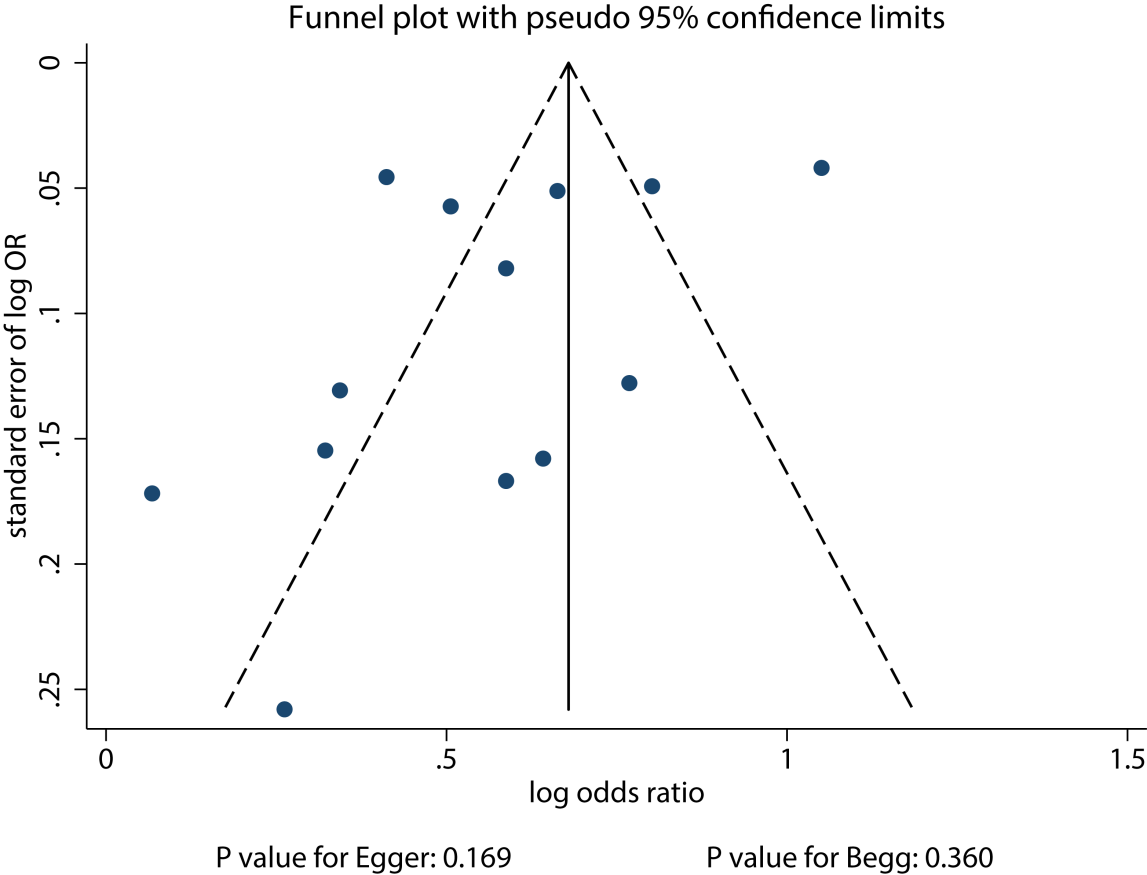


Figure S31. Funnel plot for association of arthritis with the risk of dry eye syndrome


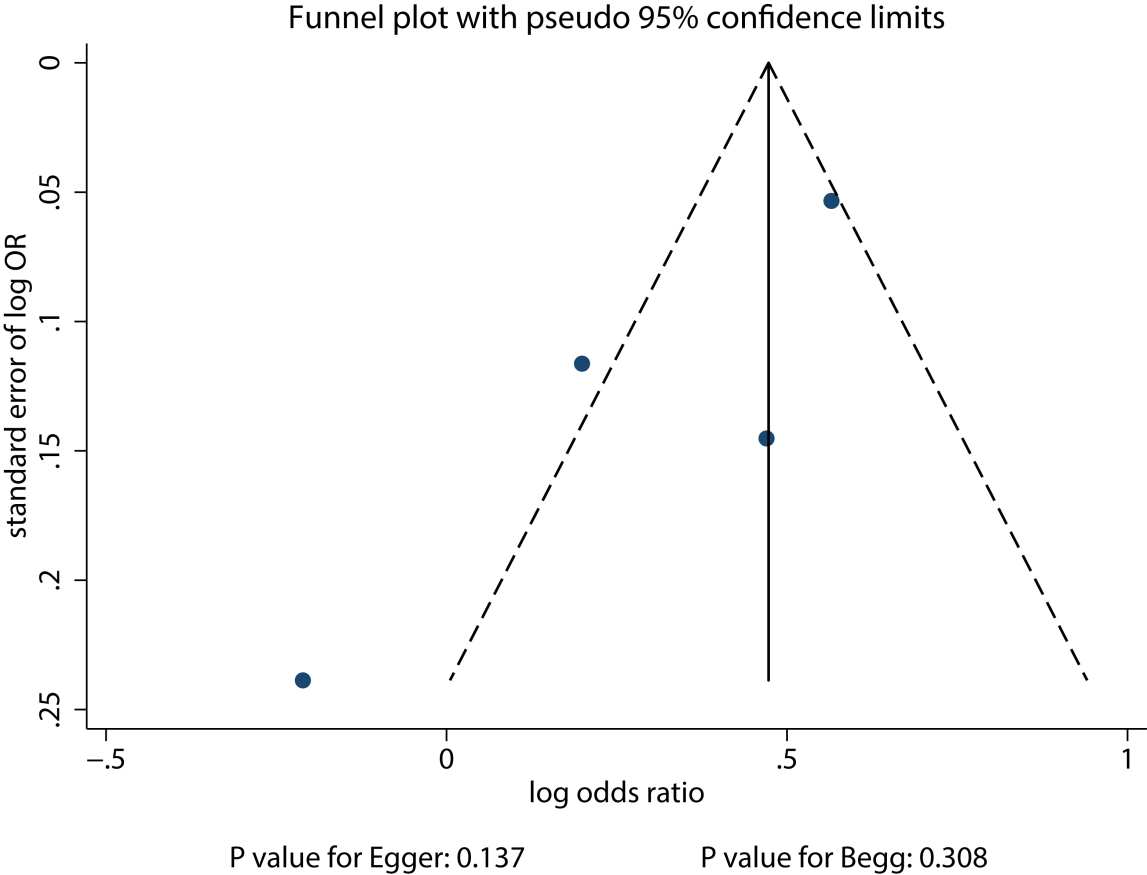


Figure S32. Funnel plot for association of osteoporosis with the risk of dry eye syndrome


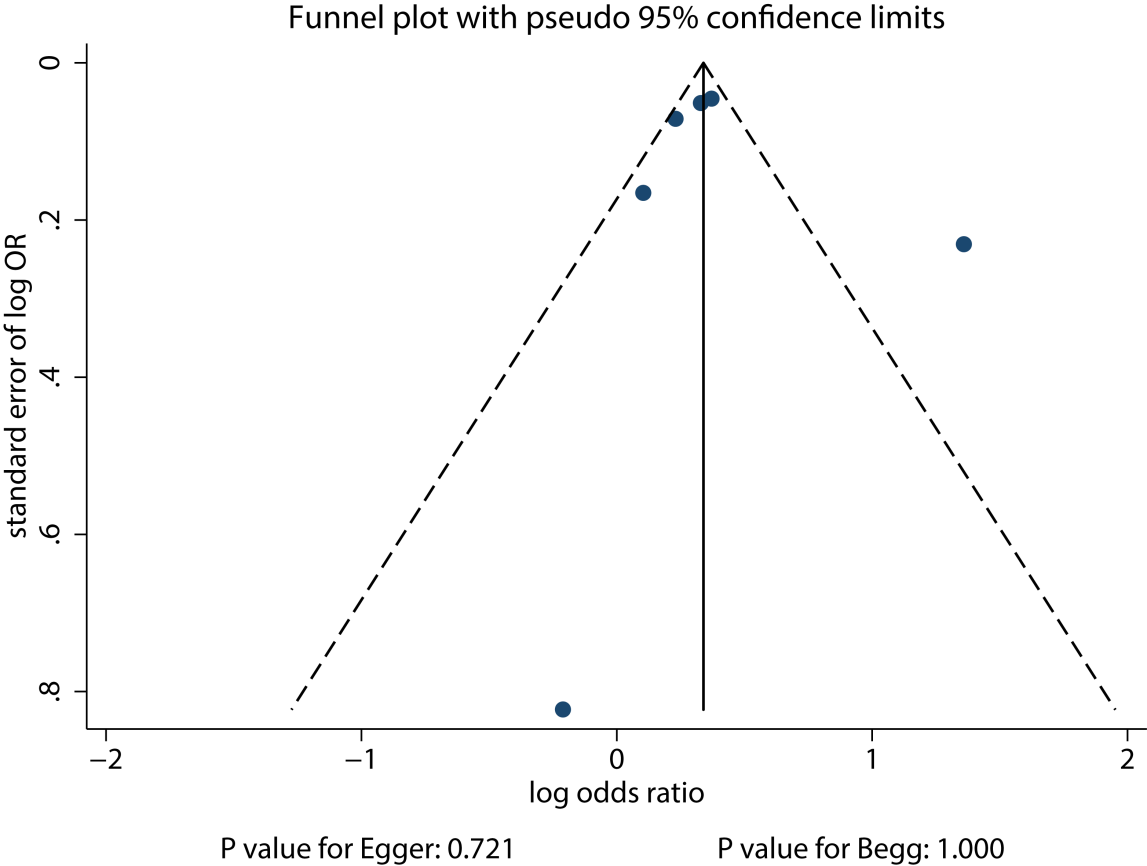


Figure S33. Funnel plot for association of tumor with the risk of dry eye syndrome
