# Supplementary material for: Identified risk factors for dry eye syndrome: A systematic review and meta-analysis
Source: PLoS One. 2022 Aug 19;17(8):e0271267. doi: 10.1371/journal.pone.0271267 (PMC9390932; doi:10.1371/journal.pone.0271267)
Supplement: Attachment — Submitted filename: Response to reviewer.docx [file pone.0271267.s008.docx]

**Reviewer #1:**

General comments: The reviewer thanks the authors for their attempt at revising the manuscript. While improved, the manuscript still has a few limitations as noted below:

Response: As behalf of all co-authors, I would like to appreciate this referee due to thoughtful comments proposed by the peer review. After we revised the manuscript, those significant issues could be changed.

Question 1: Conclusion, Abstract: “This study reported the comprehensive risk factors for dry eye syndrome, including demographic information, clinical characteristics, and comorbidities.” This sentence is not suitable as the last sentence of the abstract. Instead, the authors should discuss the implications of their research (i.e. why identification of these risk factors is important to improve clinical care) or alternatively discuss other areas of research that could be explored.

Response: Thanks for this suggestion, and the conclusion have already changed into: “This study reported risk factors for dry eye syndrome, and identified patients at high risk for dry eye syndrome.”

Question 2: The manuscript should be thoroughly proofread by a native English speaker. For instance, this sentence contains multiple grammatical errors: “Moreover, the occupational risk factor of visual display terminal (VDT) use was related to the progression of DES, which could explained by decreases blink rate and increases the proportion of incomplete blinks could causing the increased exposure of the ocular surface to the environment”. A more appropriate sentence would be: “Moreover, the occupational risk factor of visual display terminal (VDT) use was related to the progression of DES, which could be explained by a decreased blink rate and increased proportion of incomplete blinks that could be caused by the increased exposure of the ocular surface to the environment”. In some sections, the language is so poor that it is uninterpretable: “which restricting the assessment of causality relationships between risk factors and DES”.

Response: Thanks for this suggestion, and the English revision have already performed by Editage Company.

Question 3: The search strategy is still insufficient to permit replication of the search by an independent researcher. The authors should specify the search strategy in a line-by-line format in a table that outlines clearly what term was searched, how terms were combined, and whether any restrictions were applied. The search should be updated to March 2022. In the table, the authors should highlight whether each term was a MeSH subject heading or a text based term.

Response: Thanks for this suggestion, and the details search strategy in PubMed have already added in S1 file.

Question 4: The authors note that studies which assessed risk factors in at least 3 cohorts were included. The authors should cite the meta-analysis in Lancet Global Health that they followed for this methodology. I would advise changing ‘cohorts’ to ‘studies’ to make this sentence clearer in the Methods.

Response: Thanks for this suggestion, and this articles have already cited in the revised manuscript. Moreover, the ‘cohorts’ have already changed into ‘studies’.

Question 5: The authors note that “this study contained cross-sectional, retrospective, and prospective observational studies, and the GRADE evaluation was not applied for certainty of findings.” This reviewer believes it is important to conduct a GRADE evaluation for this meta-analysis especially because the evidence comes primarily from observational studies which are by their nature susceptible to bias. To provide readers with an indication of the certainty of evidence, the GRADE evaluation should be integrated.

Response: We acknowledge the importance of GRADE, while 39 of included studies designed as cross-sectional studies, and the causality relationships between risk factors and DES were not available. Moreover, the quality of included studies were assessed using the Newcastle-Ottawa Scale, and the results are listed in S1 table.

Question 6: The authors have discussed the variable definitions of DED in the limitations section, however in the baseline characteristics section of the results the authors should specify how different studies defined DED. This is important for readers to understand.

Response: Thanks for this suggestion, and the following sentence have already added in Results section: “The DES definition based on questionnaire were reported in 33 studies, 10 studies used TBUT, ST, or FSS defined DES, 3 studies applied ICD9 code and the remaining 2 studies used clinician-diagnosed defined DES.”

Question 7: The sentence “we noted that other races versus white race were associated with an increased risk of DES, which is significantly related to the climate type” should be deleted because there is not evidence to support the notion that DES in different races is attributable to climate type as opposed to other factors.

Response: Thanks for this suggestion, and this sentence have already removed in the revised manuscript.

**Reviewer #2:**

General comments: Thank you for addressing my question and improving the shortcoming section. I do not have any more questions or comments to the authors.

Response: We appreciate the reviewer given this kindly comments.
